# Jugiones A–D: Antibacterial Xanthone–Anthraquinone Heterodimers from Australian Soil-Derived *Penicillium shearii* CMB-STF067

**DOI:** 10.3390/antibiotics13010097

**Published:** 2024-01-18

**Authors:** Thulasi Sritharan, Angela A. Salim, Zeinab G. Khalil, Robert J. Capon

**Affiliations:** Institute for Molecular Bioscience, The University of Queensland, St. Lucia, QLD 4072, Australia; t.sritharan@uq.edu.au (T.S.); a.salim@uq.edu.au (A.A.S.); z.khalil@uq.edu.au (Z.G.K.)

**Keywords:** jugione, xanthone–anthraquinone, *Penicillium shearii*, cultivation profiling (MATRIX), molecular networking (GNPS), Gram-positive antibacterial, multidrug resistance (MDR)

## Abstract

The Australian roadside soil-derived fungus *Penicillium shearii* CMB-STF067 was prioritized for chemical investigation based on an SDA cultivation extract exhibiting both antibacterial properties and natural products with unprecedented molecular formulae (GNPS). Subsequent miniaturized 24-well plate cultivation profiling (MATRIX) identified red rice as optimal for the production of the target chemistry, with scaled-up cultivation, extraction and fractionation yielding four new xanthone–anthraquinone heterodimers, jugiones A–D (**1**–**4**), whose structures were assigned by detailed spectroscopic analysis and biosynthetic considerations. Of note, where **1**–**2** and **4** were active against the Gram-positive bacteria vancomycin-resistant *Enterococcus faecalis* (IC_50_ 2.6–3.9 μM) and multiple-drug-resistant clinical isolates of *Staphylococcus aureus* (IC_50_ 1.8–6.4 μM), and inactive against the Gram-negative bacteria *Escherichia coli* (IC_50_ > 30 μM), the closely related analog **3** exhibited no antibacterial properties (IC_50_ > 30 μM). Furthermore, where **1** was cytotoxic to human carcinoma (IC_50_ 9.0–9.8 μM) and fungal (IC_50_ 4.1 μM) cells, **2** and **4** displayed no such cytotoxicity (IC_50_ > 30 μM), revealing an informative structure activity relationship (SAR). We also extended the SAR study to other known compounds of this heterodimer class, which showed that the modification of ring G can reduce or eliminate the cytotoxicity while retaining the antibacterial activity.

## 1. Introduction

Soil microbes have been the source of many modern antibiotics in clinical use, including β-lactams, streptomycins, aminoglycosides and tetracyclines. With a global increase in multidrug-resistant pathogens challenging healthcare across the world, the need for new and more effective antibiotics is both urgent and compelling. As part of our ongoing search for new microbial natural products with antibiotic properties, we screened extracts obtained from agar plate cultivations of ×139 bacterial (on ISP2 medium) and ×254 fungal (on SDA or PDA media) isolates obtained from roadside soils collected in New South Wales (NSW), Australia, against Gram-positive and Gram-negative bacterial pathogens. This survey drew our attention to the extract prepared from an SDA cultivation of *Penicillium shearii* CMB-STF067, which exhibited promising Gram-positive antibacterial properties. Importantly, a Global Natural Products Social (GNPS) [1] molecular network comparison of this extract against an in-house library of ~2000 microbial extracts confirmed that CMB-STF067 was unique in producing a suite of metabolites with unprecedented molecular formulae (i.e., not attributable to any reported microbial natural product).

Capitalizing on the knowledge that the transcriptional status of natural-product biosynthetic gene clusters can be influenced by culture conditions (i.e., media composition, temperature, aeration…) [2,3,4], we employed a miniaturized 24-well plate approach to cultivation profiling known in lab as the MATRIX [5]. When integrated with UPLC-DAD and GNPS molecular networking, MATRIX analyses can be particularly effective and have proved pivotal to our prior discovery and reporting of many new natural-product structure classes (e.g., noonindoles [6], chrysosporazines [7], talarolides [8] and terreusides [9]). In this study, we employed both the standard MATRIX [5] and newly developed grain/pulse and cereal MATRIX [9] variations to probe the metabolite production capabilities of CMB-STF067.

This report provides an account of an optimized, scaled-up fermentation and chemical fractionation of CMB-STF067 to yield four new xanthone–anthraquinone heterodimers, jugiones A–D (**1**–**4**). The jugiones belong to a class of rare fungal metabolites and are the first examples of xanthone–anthraquinone heterodimers to be reported from the genus *Penicillium*. In addition to structure elucidation by detailed spectroscopic analysis, we carried out a structure activity relationship (SAR) assessment of the jugiones against vancomycin-resistant *Enterococcus faecalis* and multiple-drug-resistant isolates of *Staphylococcus aureus*, and against human colon and lung carcinoma and fungal cells. What follows is an account of these investigations.

## 2. Results and Discussion

**Figure 1:**
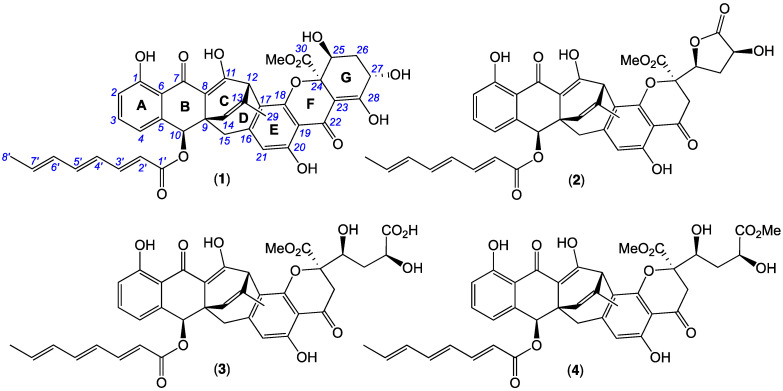
Jugiones A–D (**1–4**) from *Penicillium shearii* CMB-STF067.

MATRIX methodology comparing CMB-STF067 metabolite production in ×11 standard media compositions (Table S1) under solid phase (2.0 mL agar) as well as shaken and static broth (1.5 mL) conditions was further extended to include ×23 grain/pulse (Table S2) (grain MATRIX) and ×11 cereal (Table S3) (cereal MATRIX)-based solid media compositions, inclusive of uninoculated media controls. Subsequent in situ solvent (EtOAc) extraction followed by UPLC-DAD (Figures S5, S7 and S9) and UPLC-QTOF-MS/MS (GNPS) (Figures S6, S8 and S10) chemical profiling identified red rice as the preferred production media, with scale-up cultivation followed by solvent extraction and trituration, and gel and reversed-phase chromatography (Scheme S1) yielding the target chemistry, jugiones A–D (**1**–**4**) (Figure 1).

**Figure 2:**
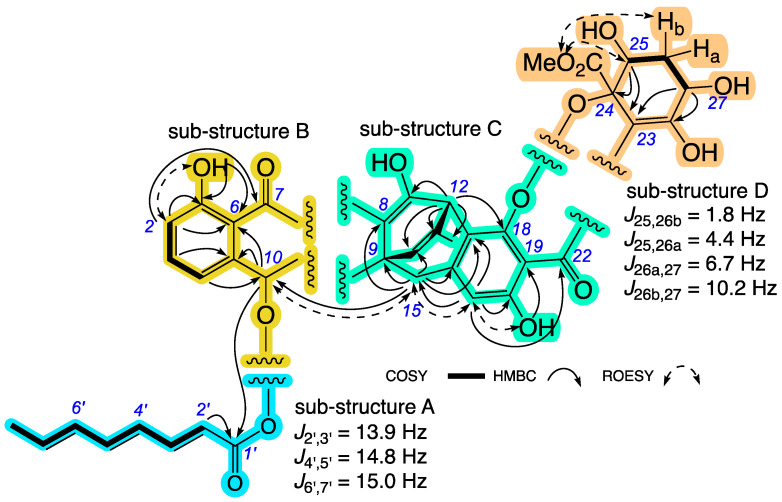
Selected NMR (CDCl_3_) correlations and *J* values for jugione A (**1**), with individual sub-structures A to D highlighted.

HRESI(+)MS measurement established a molecular formula for **1** (C_39_H_34_O_13_, ∆mmu +2.7) requiring ×23 double bond equivalents (DBEs), while analysis of the 1D and 2D NMR (CDCl_3_) (Table 1, Table 2 and Table S4, Figures S11–S16) data allowed assembly of four sub-structures A to D (Figure 2) accounting for ×21 DBE and necessitating two additional ring systems. Sub-structure A was identified as a conjugated triene octanoate with an all *E* configuration evident from diagnostic *J* values, while sub-structure B was attributed to a disubstituted phenol. An HMBC correlation from H-10 to C-1’, and from H-2 to C-7, allowed assembly of the consolidated sub-structure A–B. By contrast, sub-structures C and D featured more complex carbocycles, with an HMBC correlation from H-15 to C-10, and a ROESY correlation between H-15 and H-10, establishing a C-9 to C-10 linkage supportive of the consolidated sub-structure A–B–C. With the full suite of sub-structures A to D accounting for an oxygen atom in excess of the molecular formula necessitating a C-18 to C-24 ether bridge, the remaining disconnections and requirement for two additional rings required C-7 and C-22 ketone bridges to form rings B and F, to arrive at the complete planar structure for **1** as indicated (Figure 1).

**Figure 3:**
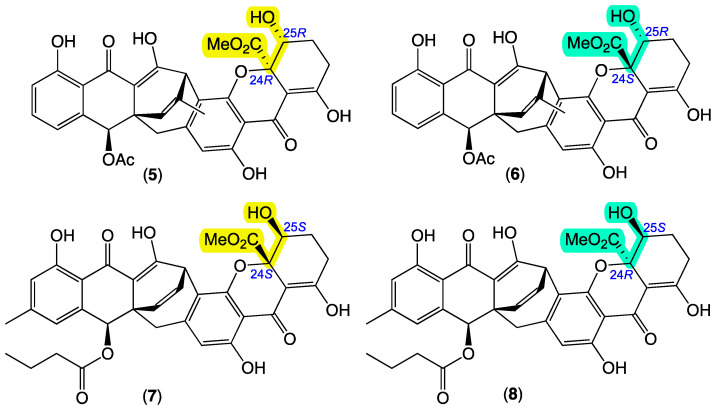
Known fungal natural products: JBIR-99 (**5**), engyodontochone B (**6**), and xanthoquinodins B10 (**7**) and B11 (**8**). Highlights (yellow and green) indicate relative configurational relationships.

In this regard, **1** shares a planar carbo/heterocyclic core framework (rings A to G) in common with JBIR-99 (**5**) first reported in 2010 [10] from an Okinawan marine sponge-derived fungus, *Tritirachium* sp. SpB081112MEf2, and subsequently re-isolated in 2021 [11] from the Indian Ocean marine seawater-derived fungus, *Meyerozyma guilliermondii* Y39, and assigned a relative configuration based on X-ray crystallographic analysis. Interestingly, a 2016 [12] re-isolation of **5** together with its isomer engyodontochone B (**6**) from a Croatian marine sponge-derived fungus, *Engyodontium album* LF069, employed ROESY and ECD correlations to assign absolute configurations to both **5** and **6** (Figure 3). Also noteworthy are the biosynthetically related xanthoquinodins B10 (**7**) and B11 (**8**), first reported in 2020 [13] from a Nepalese soil-derived fungus, *Jugulospora vestita* CBS 135.91, and assigned structures and absolute configurations based on spectroscopic and ECD analysis (Figure 3). Careful consideration of the spectroscopic data for **5**–**7**, which collectively encompass the full array of C-24/C-25 stereoisomers, reveals useful empirical rules. For example, **5** (24*R*,25*R*) and **7** (24*S*,25*S*), which feature α and β facial *cis* disposed 24-CO_2_Me and 25-OH moieties, respectively, are characterized by comparable NMR (CDCl_3_) chemical shifts for C-25 (δ_C_ 72.0 and 71.7, respectively), which differ from those of the alternate *trans* disposed isomers **6** (24*S*,25*R*) and **8** (24*R*,25*S*) (δ_C_ 66.9 and 66.9, respectively). Furthermore, the 24*R* isomers **5** and **8** possess comparable experimental ECD spectra (Figure S18), which differ from the equally comparable experimental ECD spectra reported for the 24*S* isomers **6** and **7** (Figure S19). Drawing on these NMR and ECD observations, as **1** possesses a ^13^C NMR (CDCl_3_) chemical shift for C-25 (δ_C_ 68.9) in common with **6** and **8**, and an ECD spectra (Figure S20) in common with **5** and **8**, we propose that **1** shares a 10*S*,9*R*,12*S*,24*R*,25*S* configuration in common with **8**. Finally, a ROESY correlation between 24-CO_2_CH_3_ and H_b_-26 in **1**, together with a *J*_26b,27_ of 10.2 Hz, is consistent with a large H_b_-26/H-27 dihedral angle and 27*S* configuration. This hypothesis was further validated by the consideration of predicted dihedral angles and *J* values about ring G in energy-minimized models of **1** and its unnatural 27*R* epimer (Figure S21). Based on all of the above, the structure inclusive of absolute configurations for jugione A (**1**) was assigned as shown.

**Figure 4:**
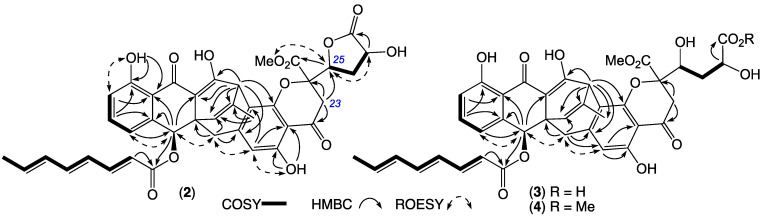
Selected 2D NMR correlations for jugione B (**2**) (CDCl_3_) and jugiones C–D (**3**–**4**) (methanol-*d*_4_).

**Figure 5:**
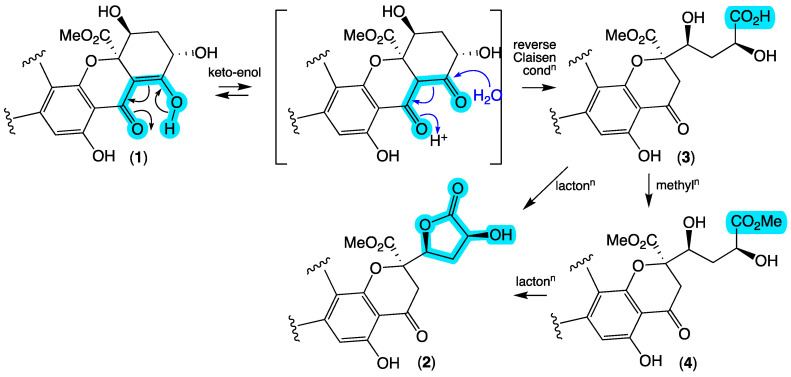
Plausible biomimetic relationship between jugiones A–D (**1**–**4**). Highlights (blue) indicate key functional groups and points of differentiation.

HRESI(+)MS measurement established a molecular formula for **2** (C_39_H_34_O_13_, ∆mmu +1.4) isomeric with **1**. Comparison of the 1D and 2D NMR (CDCl_3_) data for **2** (Table 1, Table 2 and Table S6, Figure 4 and Figures S22–S27) with **1** allowed the key differences to be attributed to a reverse Claisen condensation-like opening of ring G (Figure 5) with concomitant replacement of the sp^2^ quaternary C-23 in **1** (δ_C_ 99.8) with a diastereotopic H_2_-23/C-23 methylene in **2** (δ_H_ 3.18/3.02, AB_q_, *J* 17.0 Hz; δ_C_ 38.2) and associated formation of a sidechain butyrolactone moiety, as evident from deshielding of H-25 in **2** compared to **1** (∆δ_H_ +0.37). On the basis of the spectroscopic similarities between **1** and **2** including near-identical ECD spectra (Figure S44) and biogenetic considerations, the structure inclusive of absolute configuration for jugione B (**2**) was assigned as shown.

HRESI(+)MS measurements established a molecular formula for **3** (C_39_H_36_O_14_, ∆mmu +1.6) and its homolog (+CH_2_) **4** (C_40_H_38_O_14_, ∆mmu –0.5), with a comparison of the NMR (methanol-*d*_4_) data for **3** and **4** (Table 1, Table 2, Tables S9 and S10, Figure 4 and Figures S31–S36, S38–S42) with that for **2** (Table S7, Figures S29 and S30) allowing key differences to be attributed to the hydrolysis and methanolysis of the butyrolactone moiety, respectively. More specifically, the chemical shifts for the H-25 methine in **3** (δ_H_ 4.31, br d, *J* 10.9 Hz) and **4** (δ_H_ 4.33, br d, *J* 10.8 Hz) were shielded and consistent with a hydroxy methine, relative to the lactone methine in **2** (δ_H_ 5.00, dd, *J* 10.1 and 6.2 Hz). These observations, taken together with **3** and **4** possessing comparable ECD spectra to **1** and **2** (Figure S44) and biosynthetic considerations, supported the assignment of structures for jugiones C–D (**3**–**4**) as shown.

Alert to the possibility that chemically reactive natural products can form artifacts during handling and storage [14], careful analysis of fresh CMB-STF067 extracts prior to chemical fractionation detected **1**–**4** and confirmed their status as natural products. Supportive of this, no chemical stability issues were observed during the handling and storage of pure samples of **1**–**4**.

Jugiones belong to a rare class of xanthone–anthraquinone heterodimers whose biosynthesis has been proposed to originate from two C_16_ polyketide units [15]. Known fungal metabolites belonging to this family, including beticolins [16], cebetins [17], xanthoquinodins [13,15,18,19,20,21,22,23], JBIRs [10,11,12], engyodontochones [12], acremoxanthones [24,25,26,27] and acremonidins [28], can be grouped into four scaffolds (i–iv, Figure S46) based on the mode of dimerization between xanthone and anthraquinone monomers. Beticolins [16] (scaffold i) and cebetins [17] (scaffold ii) (Figure S47) produced by the fungal plant pathogen *Cercospora beticola* are the only examples with a chlorinated xanthone moiety and a C-13 to C-14 epoxide. Excluding beticolins and cebetins, all known compounds belonging to scaffolds (i) (e.g., JBIR-97/98 and engyodonthochone A) and (ii) (e.g., JBIR-99 and engyodonthochone B) have been isolated from marine-derived fungi [10,11,12], while those featuring scaffolds (iii) (e.g., xanthoquinodin A series and acremoxanthones A–B) and (iv) (e.g., xanthoquinodin B series and acremoxanthones C–E) have been isolated from soil-, insect- and plant-associated fungi [13,15,18,19,20,21,22,23,24,25,26,27,28]. The jugiones belonging to scaffold ii are the first xanthone–anthraquinone heterodimers to be reported from the Eurotiomycetes class—the soil-associated fungus *P. shearii* CMB-STF067 (family Aspergillaceae, order Eurotiales, class Eurotiomycetes). All reported heterodimers, with the exception of beticolins and cebetins produced by *C. beticola* (family Mycospharerellaceae, order Capnodiales, class Dothideomycetes), were produced by fungi from the Sordariomycetes class (albeit from various orders, families and genera).

The antibacterial activity against drug-sensitive *Staphylococcus aureus* ATCC 25923 that prompted our initial interest in CMB-STF067 was shown to be due to **1**–**2** and **4** (IC_50_ 1.8 to 6.6 μM), with the closely related carboxylic acid **3** being inactive (IC_50_ >30 μM) (Table 3 and Figure S45). Of note, this antibacterial activity extended to both drug-sensitive and resistant *Enterococcus faecalis* strains (IC_50_ 0.5 to 1.8 μM and 2.6 to 3.9 μM, respectively) and multiple-drug-resistant strains of *S. aureus* (IC_50_ 1.8 to 6.4 μM) (Table 3). Notwithstanding, one of the challenges associated with discovering new natural product antibiotics is the need for selectivity, particularly in favor of pathogens over host (human) cells. To address this challenge, we assessed the ability of **1**–**4** to inhibit the growth of eukaryote cells, namely human colon and lung carcinoma and fungal cells. Significantly, while **1** was cytotoxic to carcinoma (IC_50_ 9–10 μM) and fungal (IC_50_ 4.1 μM) cells, the γ-lactone- and *seco*-ring G analogs **2** and **4**, respectively, displayed no such cytotoxicity (IC_50_ >30 mM) and were selectively effective against Gram-positive bacteria (Table 3 and Figure S45).

Similar SARs have been observed for JBIR-97/98 and engyodontochones A and C (heterodimer scaffold i) and JBIR-99 and engyodontochones B, E and F (heterodimer scaffold ii) (Figure S48), where metabolites inclusive of ring G exhibit antibacterial, antifungal and cytotoxic properties, while those featuring a *seco*-ring G (including the γ-lactone) are antibacterial against Gram-positive bacteria but do not exhibit cytotoxicity towards eukaryote cells [12]. It has also been reported that xanthoquinodins A6-A8 (heterodimer scaffold iii) and xanthoquinodins B11, B14 and B15 (heterodimer scaffold iv) (Figure S48) with an intact ring G are antibacterial and cytotoxic to eukaryotic cells (fungi and human), while *seco*-ring G analogs (included the γ-lactone) are antibacterial but with reduced cytotoxicity to eukaryotic cells [13,21].

## 3. Materials and Methods

### 3.1. Collection and Isolation of CMB-STF067

The fungus CMB-STF067 was isolated from roadside soil collected in 2019 near Jugiong, NSW, Australia. The soil sample was transported to the laboratory in a sealed container at room temperature, after which, a portion (1 g) in sterile water (10 mL) was heated for 30 min at 55°C, with an aliquot (100 μL) serially diluted and applied to SDA and ISP-2 agar plates supplemented with both cycloheximide (100 μg/mL) and rifampicin (5 μg/mL). The plates were sealed with parafilm and incubated at 29 °C with periodic inspection over 4 weeks. The fungus CMB-STF067 (Figure S1) was manually recovered by colony picking from the SDA plate, and after recultivation on SDA medium, the pure isolate was cryopreserved at –80 °C in 15% aqueous glycerol.

### 3.2. Taxonomic Identification of CMB-STF067

Genomic DNA was extracted from the mycelia of an SD static broth culture of CMB-STF067 using the DNeasy Plant Mini Kit (QIAGEN) as per the manufacturer’s protocol. The 18S rRNA genes were amplified by PCR using the universal primers ITS 1 (5′-TCCGTAGGTGAACCTGCGG-3′) and ITS 4 (5ʹ TCCTCCGCTTATTGATATGC-3ʹ) purchased from Sigma-Aldrich (Merck, Darmstadt, Germany). The PCR mixture (50 μL) contained genomic DNA (2 μL, 20–40 ng), EmeraldAmpn GT PCR Master Mix (2× Premix, 25 μL), primer (0.2 μM, each) and H_2_O (up to 50 μL). PCR was performed using the following conditions: initial denaturation at 95 °C for 2 min, 40 cycles in series of 95 °C for 20 s (denaturation), 56 °C for 20 s (annealing) and 72 °C for 30 s (extension), followed by one cycle at 72 °C for 5 min. The PCR products were purified with a PCR purification kit (QIAGEN). Amplification products were examined by agarose gel electrophoresis. The DNA sequencing was performed by the Australian Genome Research Facility (AGRF) at The University of Queensland. A GenBank BLAST analysis (NCBI database) (Figure S2) on the resulting ITS gene sequence (accession number OR730993) and following a phylogenetic analysis revealed 100.0% identity with the fungal strain *Penicillium shearii* (Figure S3).

### 3.3. UPLC-DAD Profiling of MATRIX Extracts

Aliquots (1 μL, 5 mg/mL MeOH) of all MATRIX extracts prepared as noted above were analyzed by UPLC-DAD using the method outlined in the general experimental procedure (Figures S5, S7 and S9).

### 3.4. GNPS Molecular Network Profiling of MATRIX Extracts

Aliquots (1 μL, 0.05 mg/mL MeOH) of MATRIX extracts prepared as noted above were subjected to UPLC-QTOF analysis using the method outlined in the general experimental procedure. The resulting MS/MS Mass Hunter data files (.d) were converted to .mzXML file format using MS convert software version 3.0 [29] prior to being uploaded to the GNPS platform (gnps.ucsd.edu, accessed on 24 October 2023) [1] through FTP approaches (FileZilla). A molecular network analysis was performed using an online workflow at GNPS, setting the minimum cluster size at 2, cosine score 0.5 and the minimum number of fragments at 6. The spectral networks were imported into Cytoscape (version 3.9.1) [30] and visualized using a ball–stick layout where nodes represented parent masses and edge thickness corresponded to cosine score (Figures S4, S6, S8 and S10).

### 3.5. Scaled-Up Cultivation and Chemical Fractionation of CMB-STF067

The UPLC and GNPS profiling as outlined above prompted the optimized scaled-up cultivation of CMB-STF067 on red rice. To this end, cells recovered from a 7-day inhibitory mold agar (IMA, Table S1) cultivation of CMB-STF067 were used to inoculate flasks (2 × 2 L) containing red rice media (140 g, 200 mL distilled water), which were subsequently incubated at 27 °C for 21 days. After incubation, the red rice cultures were extracted with EtOAc (4 × 500 mL), and the organic phase was concentrated in vacuo at 40 °C to yield an extract (2.37 g) which was subjected to sequential trituration followed by concentration under N_2_ at 40 °C to yield an *n*-hexane (201 mg) and a combined CH_2_Cl_2_ and MeOH (1.80 g) soluble extract. The latter was subjected to gel chromatography (Sephadex LH-20, 2.5 × 85 cm, gravity isocratic elution with 50% CH_2_Cl_2_/MeOH) to yield fractions which were combined based on analytical HPLC-DAD-MS analysis. Fractions 24–25 (39.6 mg) were subjected to semi preparative HPLC (Agilent Zorbax SB-Phenyl 5 μm, 9.4 × 250 mm column, with a 3 mL/min gradient elution over 26 min from 50% H_2_O/MeCN to 30% H_2_O/MeCN inclusive of an isocratic 0.01% TFA/MeCN modifier) to afford jugione A (**1**) (*t*_R_ 22.8 min, 6.0 mg, 0.33%), jugione B (**2**) (*t*_R_ 23.6 min, 6.1 mg, 0.34%), jugione C (**3**) (*t*_R_ 17.6 min, 2.1 mg, 0.12%) and jugione D (**4**) (*t*_R_ 22.3 min, 1.5 mg, 0.08%), as summarized in Scheme S1. (Note: % yields determined on a mass-to-mass basis against the weight of the EtOAc extract.)

*jugione A* (***1***). Pale yellow powder; [α]_D_^26^ + 359.4 (*c* 0.05, MeOH); UV-vis (UPLC-DAD, MeCN/H_2_O) λ_max_ 308, 358 nm; 1D and 2D NMR (600 MHz, CDCl_3_); see Table 1, Table 2 & Table S4 and Figures S11–S16; HRESI(+)MS *m*/*z* 733.1924 [M + Na] ^+^ (calcd for C_39_H_34_O_13_Na, 733.1897).

*jugione B* (***2***). Pale yellow powder; [α]_D_^26^ +269.8 (*c* 0.05, MeOH); UV-vis (UPLC-DAD, MeCN/H_2_O) λ_max_ 308, 358 nm; 1D and 2D NMR (600 MHz, CDCl_3_); see Table 1, Table 2 & Table S6 and Figures S22–S27; 1D and 2D NMR (600 MHz, methanol-*d*_4_); see Table S7, Figures S29 and S30; HRESI(+)MS *m*/*z* 733.1911 [M + Na]^+^ (calcd for C_39_H_34_O_13_Na, 733.1897).

*jugione C* (***3***). Pale yellow powder; [α]_D_^24^ +350.8 (*c* 0.05, MeOH); UV-vis (UPLC-DAD, MeCN/H_2_O) λ_max_ 308, 358 nm; NMR (600 MHz, methanol-*d*_4_); see Table 1, Table 2 & Table S9 and Figures S31–S36; HRESI(+)MS *m*/*z* 751.2019 [M + Na]^+^ (calcd for C_39_H_36_O_14_Na, 751.2003).

*jugione D* (***4***). Pale yellow powder; [α]_D_^24^ + 355.9 (*c* 0.05, MeOH); UV-vis (UPLC-DAD, MeCN/H_2_O) λ_max_ 308, 358 nm; NMR (600 MHz, methanol-*d*_4_); see Table 1, Table 2 & Table S10 and Figures S38–S42; HRESI(+)MS *m*/*z* 765.2154 [M + Na]^+^ (calcd for C_40_H_38_O_14_Na, 765.2159).

### 3.6. Antibacterial Assay

LB agar plates inoculated with the bacterial isolate to be tested were incubated at 37 °C for 24 h, after which, several colonies were transferred to fresh sterile LB broth which was incubated at 37 °C for 24 h, and following the measurement of optical density, the cell density was adjusted to 5 × 10^5^ CFU/mL. Analytes (**1**–**4** and controls) were dissolved in DMSO and diluted with H_2_O to afford stock solutions (600 µM, 20% DMSO) which were serially diluted with 20% DMSO to yield analyte concentrations ranging from 600 to 0.2 µM. An aliquot (10 µL) of each analyte dilution was transferred to a 96-well microtiter plate along with freshly prepared bacterium broth (190 µL) to final concentrations of 30–0.01 µM in 1% DMSO. The resulting assay plates were incubated at 37 °C for 18 h and the optical density of each well was measured spectrophotometrically at 600 nm using the POLARstar Omega plate reader (BMG LABTECH, Offenburg, Germany). Antibacterial screening was carried out against Gram-positive *Staphylococcus aureus* ATCC 25923, clinical isolates of daptomycin-resistant *Staphylococcus aureus* 587701692:1*,* methicillin-resistant *Staphylococcus aureus* AUS-RBWH-MRSA-01/02, vancomycin-resistant *Enterococcus* AUS-RBWH-VRE-01*, Enterococcus faecalis* ACM 5184 and Gram-negative *Escherichia coli* ATCC11775. The positive control was rifampicin (10 µM in 1% DMSO) and the negative control was 1% DMSO in culture broth, together with extracts prepared from LB broth medium without bacterial inoculation. Each analysis was repeated two times and the data represented graphically, and IC_50_ and MIC values were calculated using GraphPad Prism version 10.0.1 (Figure S45).

### 3.7. Antifungal Assay

SD agar plates inoculated with *Candida albicans* ATCC 10231 were incubated at 27 °C for 48 h, after which, several colonies were transferred to fresh sterile SD broth (4 mL) which was incubated at 27 °C for 48 and, following the measurement of optical density, the cell density was adjusted to 5 × 10^5^ CFU/mL. An aliquot (10 µL) of analytes as prepared above for antibacterial assays was transferred to a 96-well microtiter plate and freshly prepared fungal broth (190 µL) was added to each well to give final concentrations of 30–0.01 µM in 1% DMSO. The resulting assay plates were incubated at 27 °C for 48 h and the optical density of each well was measured spectrophotometrically at 600 nm using the POLARstar Omega plate reader (BMG LABTECH, Offenburg, Germany). The positive control was amphotericin (10 µM in 1% DMSO) and the negative control was 1% DMSO, together with extracts prepared from SD broth without fungal inoculation. Each analysis was repeated two times and the data represented graphically, and IC_50_ and MIC values were calculated using GraphPad Prism version 10.0.1 (Figure S45).

### 3.8. Cytotoxic Assay

Aliquots (3000 cells/well in 190 µL of Roswell Park Memorial Institute medium supplemented with 10% fetal bovine serum) of human colorectal (SW620) and lung (NCI-H460) carcinoma cells were transferred to 96-well plates and incubated at 37 °C in 5% CO_2_ for 3 days. An aliquot (10 µL) of analytes as prepared above for antibacterial assays was transferred to a 96-well microtiter plate and incubated again for 24 h, after which, an aliquot (10 µL) of a solution of 3-(4,5-dimethylthiazol-2-yl)-2,5-diphenyltetrazolium bromide (MTT) in phosphate-buffered saline (5 mg/mL) was added to each well which were again incubated for 4 h. The media were then carefully removed (pipette) and the residue dissolved in DMSO (100 µL) by shaking at 50 rpm for 2 min. Finally, the absorbance of each well was measured spectrophotometrically at 600 nm using the POLARstar Omega plate reader (BMG LABTECH, Offenburg, Germany). The positive control was sodium dodecyl sulfate (SDS) and the negative control was 1% DMSO. Each analysis was repeated two times and the data represented graphically, and IC_50_ and MIC values were calculated using GraphPad Prism version 10.0.1 (Figure S45).

## 4. Conclusions

The jugiones are rare xanthone–anthraquinone heterodimers, which were only produced by one *Penicillium* strain (class Eurotiomycetes) in our *in-house* soil-derived microbe collection (~2000 isolates). Such heterodimers have only previously been reported from fungi belonging to the Sordariomycetes class isolated from various substrates (marine sponges, soil, insects and plants). An SAR assessment of jugiones A-D determined that **1** was antibacterial against vancomycin-resistant *Enterococcus faecalis* and multiple-drug-resistant isolates of *Staphylococcus aureus* and was cytotoxic to human carcinoma cells, while **2** and **4** retained antibacterial properties but were not cytotoxic, and **3** was neither antibacterial nor cytotoxic.

While most xanthone–anthraquinone heterodimers are cytotoxic towards eukaryotic cells, our investigations into the jugiones reveal that ring G modification can reduce cytotoxicity while retaining the antibacterial activity—as evidenced by jugiones B and D which exhibit antibacterial activity against both drug-susceptible and resistant Gram-positive bacteria but display no cytotoxicity against eukaryotic cells. Given the increase in multidrug-resistant bacteria in the community, jugiones and other related heterodimers could be viewed as antibacterial scaffolds with potential.

## Figures and Tables

**Table 1:** ^1^H NMR data for jugiones A–B (**1**–**2**) in CDCl_3_ and jugiones C–D (**3**–**4**) in methanol-*d*_4_.

	(1) δ_H_, Mult (*J* in Hz)	(2) δ_H_, Mult (*J* in Hz)	(3) δ_H_, Mult (*J* in Hz)	(4) δ_H_, Mult (*J* in Hz)
2	6.97, dd (8.6, 0.9)	6.97, dd (8.4, 0.7)	6.94, d (8.4)	6.95, d (8.3)
3	7.45, dd (7.5, 8.6)	7.44, dd (8.4, 7.3)	7.48, dd (8.4, 7.3)	7.49, dd (8.3, 7.2)
4	7.09, dd (7.5, 0.9)	7.09, dd (7.3, 0.7)	7.09, d (7.3)	7.09, d (7.2)
10	6.05, s	6.05, s	6.07, br s	6.08, s
12	4.52, d (1.5)	4.48, d (1.4)	4.74, s	4.71, s
14	5.68, dq (1.5, 1.5)	5.68, dq (1.4, 1.4)	5.68, dq (1.4, 1.4)	5.70, s
15a	2.77, d (17.8)	2.77, d (17.8)	2.83, d (17.8)	2.84, d (17.8)
15b	2.68, d (17.8)	2.67, d (17.8)	2.60, d (17.8)	2.61, d (17.8)
21	6.17, s	6.16, s	6.11, s	6.12, s
23a	-	3.18, d (17.0)	3.22, d (17.3)	a. 3.21, d (17.3)
23b	-	3.02, d (17.0)	3.10, d (17.3)	b. 3.08, d (17.3)
25	4.59, dd (4.4, 1.8)	4.96, dd (9.8, 6.2)	4.31, br d (10.9)	4.33, d (10.8)
26a	2.62, ddd (14.1, 6.7, 4.4)	2.74, ddd (13.0, 8.9, 6.2)	2.04, ddd (14.3, 10.9, 3.0)	1.99, dd (13.2, 10.8)
26b	1.98, ddd (14.1, 10.2, 1.8)	2.34, ddd (13.0, 9.8, 9.8)	1.85, dd (14.3, 9.8)	1.87, dd (13.2, 10.8)
27	4.81, dd (10.2, 6.7)	4.70, dd (9.8, 8.9)	4.25, dd (9.8, 3.0)	4.45, dd (10.8, 2.4)
29	1.86, d (1.5)	1.88, dd (1.4, 1.4)	1.91, br s	1.91, br s
2′	5.75, d (13.9)	5.74, d (15.2)	5.76, d (15.2)	5.75, d (15.2)
3′	7.24, dd (13.9, 11.2)	7.23, dd (15.2, 11.2)	7.22, dd (15.2, 11.3)	7.22, dd (15.2, 11.3)
4′	6.12, dd (14.8, 11.2)	6.12, dd (14.8, 11.2)	6.21, dd (14.9, 11.3)	6.21, dd (14.8, 11.3)
5′	6.52, dd (14.8, 10.8)	6.52, dd (14.8, 10.8)	6.58, dd (14.9, 10.7)	6.58, dd (14.8, 11.0)
6′	6.12, dd (15.0, 10.8)	6.13, dd (14.8, 10.8)	6.16, dd (14.9, 10.7)	6.16, dd (15.0, 11.0)
7′	5.95, dq (15.0, 6.8)	5.95, dq (14.8, 6.9)	5.98, dq (14.9, 6.8)	5.98, dq (15.0, 6.8)
8′	1.82, d (6.8)	1.82, d (6.9)	1.79, d (6.8)	1.79, d (6.8)
30-OMe	3.75, s	3.76, s	3.67, s	3.66, s
28-OMe	-	-	-	3.75, s
1-OH	11.58, s	11.65, s		
11-OH	14.21 ^A^, br s	14.11, s		
20-OH	11.05, s	11.31, s		
28-OH	14.06 ^A^, br s			-

^A^ assignments are interchangeable.

**Table 2:** ^13^C NMR data for jugiones A–D (**1**–**4**).

	(1) δ_C,_ Mult (CDCl_3_)	(2) δ_C,_ Mult (CDCl_3_)	(2) δ_C,_ Mult (methanol-*d*_4_)	(3) δ_C,_ Mult (methanol-*d*_4_)	(4) δ_C,_ Mult (methanol-*d*_4_)
1	161.9, C	161.9, C	162.9, C	162.8, C	163.1, C
2	119.5, CH	119.5, CH	120.1, CH	120.0, CH	120.1, CH
3	136.3, CH	136.2, CH	137.2, CH	137.0, CH	137.1, CH
4	122.2, CH	122.2, CH	123.1, CH	123.0, CH	123.1, CH
5	137.2, C	137.2, C	138.7, C	138.7, C	138.7, C
6	115.2, C	115.2, C	116.1, C	116.2, C	116.2, C
7	186.7, C	186.9, C	188.3, C	ND	ND
8	106.6, C	106.4, C	107.6, C	107.5 ^B^, C	107.5 ^B^, C
9	41.5, C	41.5, C	42.7, C	42.8, C	42.7, C
10	72.8, CH	72.8, CH	74.3, CH	74.5, CH	74.4, CH
11	185.8, C	185.8, C	186.4, C	187.4 ^B^, C	187.3 ^B^, C
12	43.4, CH	43.3, CH	44.3, CH	44.4, CH	44.4, CH
13	141.4, C	141.6, C	142.9, C	143.2, C	143.0, C
14	125.9, CH	125.8, CH	126.8, CH	126.6, CH	126.8, CH
15	35.9, CH_2_	36.0, CH_2_	36.4, CH_2_	36.6, CH_2_	36.5, CH_2_
16	148.7, C	149.6, C	150.4, C	150.0, C	150.1, C
17	115.3 ^A^, C	115.0, C	116.2, C	116.2 ^B^, C	116.1, C
18	154.1, C	155.2, C	156.9, C	157.6, C	157.4, C
19	105.4, C	105.9, C	106.8, C	106.9, C	106.9, C
20	160.4, C	160.2, C	161.1, C	161.0, C	161.1, C
21	115.2 ^A^, CH	114.4, CH	114.6, CH	114.1, CH	114.2, CH
22	188.0, C	193.9, C	196.1, C	198.1, C	197.9, C
23	99.8, C	38.2, C	39.6, CH_2_	40.6, CH_2_	40.3, CH_2_
24	84.3, C	84.2, C	85.4, C	89.2, C	89.2, C
25	68.9, CH	77.7, CH	78.8, CH	72.8, CH	71.7, CH
26	32.4, CH_2_	31.5, CH_2_	32.4, CH_2_	37.7, CH_2_	36.9, CH_2_
27	64.1, CH	67.7, CH	68.4, CH	69.5, CH	68.3, CH
28	176.8, C	175.4, C	177.6, C	180.3, C	176.6, C
29	21.0, CH_3_	21.0, CH_3_	20.8, CH_3_	20.7, CH_3_	20.7, CH_3_
30	170.5, C	168.8, C	170.6, C	171.9, C	171.8, C
1′	166.7, C	166.7, C	167.8, C	167.8, C	167.8, C
2′	119.2, CH	119.3, CH	119.9, CH	120.0, CH	120.0, CH
3′	146.5, CH	146.4, CH	147.6, CH	147.6, CH	147.6, CH
4′	127.5, CH	127.5, CH	128.6, CH	128.6, CH	128.6, CH
5′	142.2, CH	142.2, CH	143.4, CH	143.4, CH	143.4, CH
6′	131.4, CH	131.4, CH	132.5, CH	132.5, CH	132.5, CH
7′	136.1, CH	136.0, CH	136.7, CH	136.6, CH	136.7, CH
8′	18.8, CH_3_	18.8, CH_3_	18.6, CH_3_	18.6, CH_3_	18.6, CH_3_
30-OMe	54.0, CH_3_	54.2, CH_3_	54.0, CH_3_	53.4, CH_3_	53.5, CH_3_
28-OMe					52.6, CH_3_

^A^ resonances with the same superscript within a column are interchangeable, ^B^ detected by HMBC. ND: resonance is not detected.

**Table 3:** Biological properties (IC_50_ μM) of jugiones A–D (**1–4**).

Assay Type	(1)	(2)	(3)	(4)
Antibacterial–Gram-positive				
*Staphylococcus aureus* (ATCC 25923)	1.8	6.6	>30	3.7
*Staphylococcus aureus* ^A^ (581101692:1)	1.8	5.8	>30	4.3
*Staphylococcus aureus* ^B^ (AUS-RBWH-MRSA-01)	2.4	6.4	>30	3.7
*Enterococcus faecalis* ^C^ (AUS-RBWH-VRE-01)	2.6	3.9	>30	3.7
*Enterococcus faecalis* ^D^ (ACM 5184)	1.3	1.8	>30	0.5
Antibacterial–Gram-negative				
*Escherichia coli* (ATCC 11775)	>30	>30	>30	>30
Antifungal				
*Candida albicans* (ATCC 10231)	4.1	>30	>30	>30
Anticancer–cell cytotoxicity				
Human colon carcinoma (SW620)	9.8	>30	>30	>30
Human lung carcinoma (NCI-H460)	9.0	>30	>30	>30

Clinical isolates resistant to ^A^ daptomycin, ^B^ methicillin and ^C^ vancomycin. ^D^ Vancomycin-susceptible clinical isolate.

## Data Availability

Raw NMR data for **1**–**4** have been deposited in the Natural Product Magnetic Resonance Database Project (NP-MRD). The MS/MS data (.mzXML) for **1**–**4** can be accessed from https://massive.ucsd.edu/ProteoSAFe/dataset.jsp?task=a8234245cf1f4e8a82be997c44676646, accessed on 6 December 2023.

## Supplementary Materials

The following supporting information can be downloaded at: https://www.mdpi.com/article/10.3390/antibiotics13010097/s1, general experimental procedures; MATRIX methodology; GNPS analysis; MATRIX study of CMB-STF067; NMR spectroscopic data (tabulated data and spectra of **1***–***4**); ECD spectra; biological assay graphs; structures of known xanthone-anthroquinone dimers.
