# Supplementary material for: Jugiones A–D: Antibacterial Xanthone–Anthraquinone Heterodimers from Australian Soil-Derived Penicillium shearii CMB-STF067
Source: Antibiotics (Basel). 2024 Jan 18;13(1):97. doi: 10.3390/antibiotics13010097 (PMC10812710; doi:10.3390/antibiotics13010097)
Supplement: Supplementary file 1 [file antibiotics-2787250-supplementary.pdf › antibiotics-2787250-supplementary.pdf]

Supplementary Materials

Jugiones A-D: Antibacterial Xanthone-Anthraquinone Heterodimers from Australian Soil-Derived *Penicillium shearii* CMB-STF067

Thulasi Sritharan, Angela A. Salim, Zeinab Khalil and Robert J. Capon*

Institute for Molecular Bioscience, The University of Queensland, St. Lucia, QLD, 4072, Australia

Table of contents

1. Experimental **4**
1.1. Image of IMA plate culture of *Penicillium shearii* CMB-STF067 4
1.1.1. ITS gene sequence of CMB-STF067 4
1.1.2. Phylogenetic Tree 6
1.2. General experimental 7
1.3. Global Natural Product Social (GNPS) Molecular Networking 8
1.4. MATRIX Chemical Profiling 10
1.4.1. Standard MATRIX 10
1.4.2. Grain MATRIX 12
1.4.3. Cereal MATRIX 14
1.5. Fractionation of jugiones A–D 16
2. Spectroscopic characterization of jugiones A–D (1-4) **17**
2.1. Jugione A (1) 17
2.2. Jugione B (2) 25
2.3. Jugione C (3) 33
2.4. Jugione D (4) 38
3. Biological assay results for jugiones A–D (1-4) **44**

List of Schemes

Scheme S1. Isolation scheme for CMB-STF067 cultured in red rice grain and extracted by EtOAc (a) solvent trituration (b) Sephadex LH-20 (DCM/MeOH) chromatography, and (c) semi-preparative HPLC. 16

List of Tables

Table S1. List of media used in MATRIX cultivation profiling of CMB-STF067. 10
Table S2.: List of grains/pulses used for grain MATRIX profiling 12
Table S3: List of breakfast cereals used for cereal MATRIX 14
Table S4. NMR (600 MHz, $CDCl_3$) data for jugione A (**1**) 17
Table S5. Comparison of NMR data ($CDCl_3$) for JBIR-99 (**5**), engyodontochones B (**6**) and xanthoquinodins B10-B11 (**7-8**) with jugione A (**1**) 22
Table S6. 1D NMR (600 MHz, $CDCl_3$) data for jugione B (**2**) 25
Table S7. NMR (600 MHz, methanol-d_4) data for jugione B (**2**) 30
Table S8. Comparison of 1D NMR (methanol-d_4) data for engyodontochones D (**7**) with jugione B (**2**) 32
Table S9. NMR (600 MHz, methanol-d_4) data for jugione C (**3**) 33
Table S10. NMR (600 MHz, methanol-d_4) data for jugione D (**4**) 38

List of Figures

Figure S1. Fungal strain *Penicillium shearii* (CMB-STF067) growing on an IMA agar 4
Figure S2. BLAST search (closest match) of CMB-STF067 18S rRNA sequence. 5
Figure S3. Phylogenetic analysis of CMB-STF067 using PhyML Maximum Likelihood analysis of ITS DNA sequences using the optimal nucleotide substitution model determined by jModeltest2 using UGENE software. Phylogenetic relationship of CMB-STF067 with selected reference strains shown with their accession numbers indicated in red in colour. 6
Figure S4. GNPS molecular network of extracts obtained from (a) x176 NSW soil-derived fungi and (b) x1876 Capon lab microbial collection including CMB-STF067. Expanded molecular family for the jugiones, unique to CMB-STF067 9
Figure S5. UPLC-DAD (210 nm) traces of CMB-STF067 cultured in different MATRIX culture conditions, with media blanks and an internal calibrant (*). Red highlighted peaks: jugiones A-D. 11
Figure S6. GNPS molecular network of CMB-STF067 in the selected MATRIX media, with expanded view of the jugione molecular family 11

Figure S7. UPLC-DAD (210 nm) traces of CMB-STF067 cultured in different grain MATRIX culture conditions, with media blanks and an internal calibrant (*). Red highlighted peaks: jugiones A-D. 13
Figure S8. GNPS molecular network of the grain MATRIX cultivation profile of CMB-STF067 with an expanded view of the jugione molecular family. 14
Figure S9. UPLC-DAD (210 nm) traces of CMB-STF067 cultured in different cereal MATRIX culture conditions, with media blanks and an internal calibrant (*). Red highlighted peaks: jugiones A-D. 15
Figure S10. GNPS molecular network of the cereal MATRIX cultivation profile of CMB-STF067 with an expanded view of the jugione molecular family. 15
Figure S11. ^{1}H NMR (600 MHz, $CDCl_3$) spectrum of jugione A (**1**).......... 18
Figure S12. ^{13}C NMR (150 MHz, $CDCl_3$) spectrum of jugione A (**1**).......... 18
Figure S13. HSQC NMR (600 MHz, $CDCl_3$) spectrum of jugione A (**1**) 19
Figure S14. COSY NMR (600 MHz, $CDCl_3$) spectrum of jugione A (**1**) 19
Figure S15. HMBC NMR (600 MHz, $CDCl_3$) spectrum of jugione A (**1**) 20
Figure S16. ROESY NMR (600 MHz, $CDCl_3$) spectrum of jugione A (**1**).......... 20
Figure S17. HRMS spectrum and measurement for jugione A (**1**).......... 21
Figure S18. ECD spectra comparison of JBIR-99 (**5**) and xanthoquinodin B11 (**8**). The ECD spectra of these compounds are adapted from the literature.[1, 2].......... 23
Figure S19. ECD spectra comparison of engyodontochone B (**6**) and xanthoquinodin B10 (**7**). The ECD spectra of these compounds are adapted from there literature.[1, 2].......... 23
Figure S20. ECD spectra of jugione A (**1**) in MeOH (0.5 mg/mL). 24
Figure S21. Ring G in minimized models of (a) jugione A (**1**) and (b) its 27*R* epimer 24
Figure S22. ^{1}H NMR (600 MHz, $CDCl_3$) spectrum of jugione B (**2**).......... 26
Figure S23. ^{13}C NMR (150 MHz, $CDCl_3$) spectrum of jugione B (**2**).......... 26
Figure S24. HSQC NMR (600 MHz, $CDCl_3$) spectrum of jugione B (**2**).......... 27
Figure S25. COSY NMR (600 MHz, $CDCl_3$) spectrum of jugione B (**2**).......... 27
Figure S26. HMBC NMR (600 MHz, $CDCl_3$) spectrum of jugione B (**2**).......... 28
Figure S27. ROESY NMR (600 MHz, $CDCl_3$) spectrum of jugione B (**2**) 28
Figure S28. HRMS spectrum and measurement for jugione B (**2**) 29
Figure S29. ^{1}H NMR (600 MHz, methanol-d_4) spectrum of jugione B (**2**) 31
Figure S30. ^{13}C NMR (150 MHz, methanol-d_4) spectrum of jugione B (**2**) 31
Figure S31. ^{1}H NMR (600 MHz, methanol-d_4) spectrum of jugione C (**3**) 34
Figure S32. ^{13}C NMR (150 MHz, methanol-d_4) spectrum of jugione C (**3**) 34
Figure S33. HSQC NMR (600 MHz, methanol-d_4) spectrum of jugione C (**3**).......... 35
Figure S34. COSY NMR (600 MHz, methanol-d_4) spectrum of jugione C (**3**).......... 35
Figure S35. HMBC NMR (600 MHz, methanol-d_4) spectrum of jugione C (**3**).......... 36
Figure S36. ROESY NMR (600 MHz, methanol-d_4) spectrum of jugione C (**3**) 36
Figure S37. HRMS spectrum and measurement for jugione C (**3**) 37
Figure S38. ^{1}H NMR (600 MHz, methanol-d_4) spectrum of jugione D (**4**).......... 39
Figure S39. ^{13}C NMR (150 MHz, methanol-d_4) spectrum of jugione D (**4**) 39
Figure S40. HSQC NMR (600 MHz, methanol-d_4) spectrum of jugione D (**4**).......... 40
Figure S41. COSY NMR (600 MHz, methanol-d_4) spectrum of jugione D (**4**).......... 40
Figure S42. HMBC NMR (600 MHz, methanol-d_4) spectrum of jugione D (**4**) 41
Figure S43. HRMS spectrum and measurement for jugione D (**4**).......... 42
Figure S44. ECD spectra of jugiones B - C (**2**–**4**) in MeOH (0.5 mg/mL). 43
Figure S45. Antimicrobial and cytotoxicity activity of metabolites **1**- **4**. 44
Figure S46. Different scaffolds arising from the xanthone and anthroquinone dimerization………... 45
Figure S47. Structures of beticolin and cebetin……….46
Figure S48. Structures of xanthone-anthroquinone dimers……….46

1. Experimental

1.1. Image of IMA plate culture of *Penicillium shearii* CMB-STF067

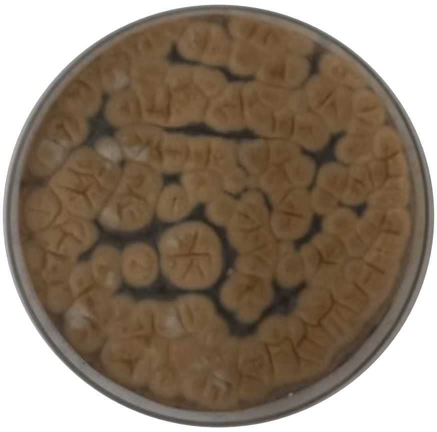

Figure S1. Fungal strain *Penicillium shearii* (CMB-STF067) growing on an IMA agar

1.1.1. ITS gene sequence of CMB-STF067

ACAGGTGGGNCCTACCTGATCCGAGGTCAACCTGAAAAATAGATTGAGGGGGTCGG
CAAGCGCCGGCCGGGCCTACAAGAGCGGGTGACAAAGCCCCATACGCTCGAGGAC
CGGACGCGGTGCCGCCGCTGCCTTTCGGGCCCGTCCCCCCGGGAGGGGGGGACGG
GGCCCAACACACAAGCCGTGCTTGAGGGCAGCAATGACGCTCGGACAGGCATGCCC
TCCGGAATACCAGAGGGCGCAATGTGCGTTCAAAGACTCGATGATTCACTGAATTC
TGCAATTCACATTATTTATCGCATTTCGCTGCGTTCTTCATCGATGCCGGAACCAAG
AGATCCGTTGTTGAAAGTTTTAACTAATTTAAATAGTTTCTCAGACTGCAACTTCAG
ACAGAGTTCACAGGTGTCTTCGGCGGGCGCGGGCCCGGGGGCGGATGCCCCCCGGC
GGCCGTGAGGCGGGCCCGCCGAAGCAACAAGGTTCGTTAAACACGGGTGGGAGGT
TGGACCCAGAGGGCCCTCACTCAGTAATGATCCTTCCGCAGGTTCNCTTTNCGGAA
GG

BLAST search result (closest match):

The strain CMB-STF067 had 100.0 % similarity with *Penicillium shearii* strain HBM7 isolated from *Psidium* sp. rhizosphere (sequence number in Genbank database – MN427870.1)

Description	Scientific Name	Max Score	Total Score	Query Cover	E value	Per. Ident	Acc. Len	Accession
Penicillium shearii strain HBM7 small subunit ribosomal RNA gene, partial sequence; internal transcribed spacer 1...	Penicillium shearii	996	996	95%	0.0	100.00%	585	MN427870.1
Penicillium shearii culture CBS:129467 strain CBS 129467 small subunit ribosomal RNA gene, partial sequence; i...	Penicillium shearii	996	996	95%	0.0	100.00%	585	MH865343.1
Penicillium shearii culture CBS:127358 strain CBS 127358 small subunit ribosomal RNA gene, partial sequence; i...	Penicillium shearii	996	996	95%	0.0	100.00%	586	MH864540.1
Penicillium shearii culture CBS:488.66 strain CBS 488.66 small subunit ribosomal RNA gene, partial sequence; int...	Penicillium shearii	996	996	95%	0.0	100.00%	586	MH858867.1
Penicillium shearii culture CBS:343.54 strain CBS 343.54 small subunit ribosomal RNA gene, partial sequence; int...	Penicillium shearii	996	996	95%	0.0	100.00%	898	MH857352.1
Penicillium shearii genomic DNA sequence contains 18S rRNA gene, ITS1, 5.8S rRNA gene, ITS2, 28S rRNA gene	Penicillium shearii	996	996	95%	0.0	100.00%	591	OW985647.1
Penicillium shearii genomic DNA sequence contains 18S rRNA gene, ITS1, 5.8S rRNA gene, ITS2, 28S rRNA gene	Penicillium shearii	996	996	95%	0.0	100.00%	593	OW985624.1
Penicillium shearii genomic DNA sequence contains 18S rRNA gene, ITS1, 5.8S rRNA gene, ITS2, 28S rRNA gene	Penicillium shearii	996	996	95%	0.0	100.00%	600	OW982845.1
Penicillium shearii genomic DNA sequence contains 18S rRNA gene, ITS1, 5.8S rRNA gene, ITS2, 28S rRNA gene	Penicillium shearii	996	996	95%	0.0	100.00%	584	OW982836.1
Penicillium shearii genomic DNA sequence contains 18S rRNA gene, ITS1, 5.8S rRNA gene, ITS2, 28S rRNA gene	Penicillium shearii	996	996	95%	0.0	100.00%	590	OW982835.1
Penicillium shearii genomic DNA sequence contains 18S rRNA gene, ITS1, 5.8S rRNA gene, ITS2, 28S rRNA gene	Penicillium shearii	996	996	95%	0.0	100.00%	591	OW982822.1
Penicillium sp. isolate CV00333 small subunit ribosomal RNA gene, partial sequence; internal transcribed spacer ...	Penicillium sp.	992	992	95%	0.0	100.00%	574	OR095933.1
Penicillium shearii culture CBS:578.70 strain CBS 578.70 small subunit ribosomal RNA gene, partial sequence; int...	Penicillium shearii	990	990	95%	0.0	99.81%	900	MH859858.1
Penicillium sp. SPB-2023a isolate CV00204 small subunit ribosomal RNA gene, partial sequence; internal transcri...	Penicillium sp. S...	990	990	95%	0.0	99.81%	573	OR096331.1
Penicillium shearii isolate V13-557 18S ribosomal RNA gene, partial sequence	Penicillium shearii	990	990	95%	0.0	100.00%	579	KJ439199.1
Penicillium sp. isolate UH.1552.166 small subunit ribosomal RNA gene, partial sequence; internal transcribed spa...	Penicillium sp.	990	990	95%	0.0	99.81%	602	MZ374584.1
Eupenicillium shearii 5.8S ribosomal RNA region	Penicillium shearii	985	985	95%	0.0	99.63%	602	AJ004893.1
Penicillium shearii isolate V26-548 18S ribosomal RNA gene, partial sequence	Penicillium shearii	983	983	95%	0.0	99.81%	581	KJ439208.1
Penicillium shearii strain SVWS2 small subunit ribosomal RNA gene, partial sequence; internal transcribed spacer...	Penicillium shearii	981	981	95%	0.0	99.45%	605	OP942451.1
Penicillium shearii isolate VJP14 internal transcribed spacer 1, partial sequence; 5.8S ribosomal RNA gene and in...	Penicillium shearii	981	981	95%	0.0	99.81%	576	OM946586.1
Eupenicillium cf. shearii Fun22C 18S ribosomal RNA gene, partial sequence	Penicillium cf. sh...	981	981	95%	0.0	99.81%	569	KF578127.1
uncultured fungus genomic DNA sequence contains 18S rRNA gene, ITS1, 5.8S rRNA gene, ITS2, 28S rRNA gen...	uncultured fungus	981	981	94%	0.0	100.00%	1453	LR993539.1
Penicillium shearii isolate HF12728 18S ribosomal RNA gene, partial sequence; internal transcribed spacer 1 and ...	Penicillium shearii	979	979	95%	0.0	99.63%	587	JQ863221.1
Penicillium shearii strain NRRL 715 internal transcribed spacer 1, 5.8S ribosomal RNA gene, and internal transcri...	Penicillium shearii	977	977	94%	0.0	100.00%	1143	AF033420.1
Penicillium shearii strain MR71 small subunit ribosomal RNA gene, partial sequence; internal transcribed spacer 1...	Penicillium shearii	976	976	93%	0.0	100.00%	550	KY320611.1
Penicillium shearii isolate AJP05 18S ribosomal RNA gene, partial sequence; internal transcribed spacer 1, 5.8S ri...	Penicillium shearii	976	976	95%	0.0	99.44%	565	KM873042.1
Penicillium shearii strain CMU18 18S ribosomal RNA gene, partial sequence; internal transcribed spacer 1, 5.8S ri...	Penicillium shearii	974	974	98%	0.0	98.55%	552	GQ924907.1
Penicillium sp. strain 637F5R-AM internal transcribed spacer 1, partial sequence; 5.8S ribosomal RNA gene and i...	Penicillium sp.	972	972	94%	0.0	99.81%	548	MG751269.1
Penicillium shearii isolate JB123 internal transcribed spacer 1, partial sequence; 5.8S ribosomal RNA gene and int...	Penicillium shearii	972	972	93%	0.0	100.00%	553	OQ332388.1
Penicillium shearii strain CV499 18S ribosomal RNA gene, partial sequence; internal transcribed spacer 1, 5.8S ri...	Penicillium shearii	972	972	94%	0.0	99.81%	560	JX140868.1
Penicillium shearii strain SS133-1 internal transcribed spacer 1, partial sequence; 5.8S ribosomal RNA gene and i...	Penicillium shearii	972	972	94%	0.0	99.81%	1129	AY232278.1

Figure S2. BLAST search (closest match) of CMB-STF067 18S rRNA sequence.

1.1.2. Phylogenetic Tree

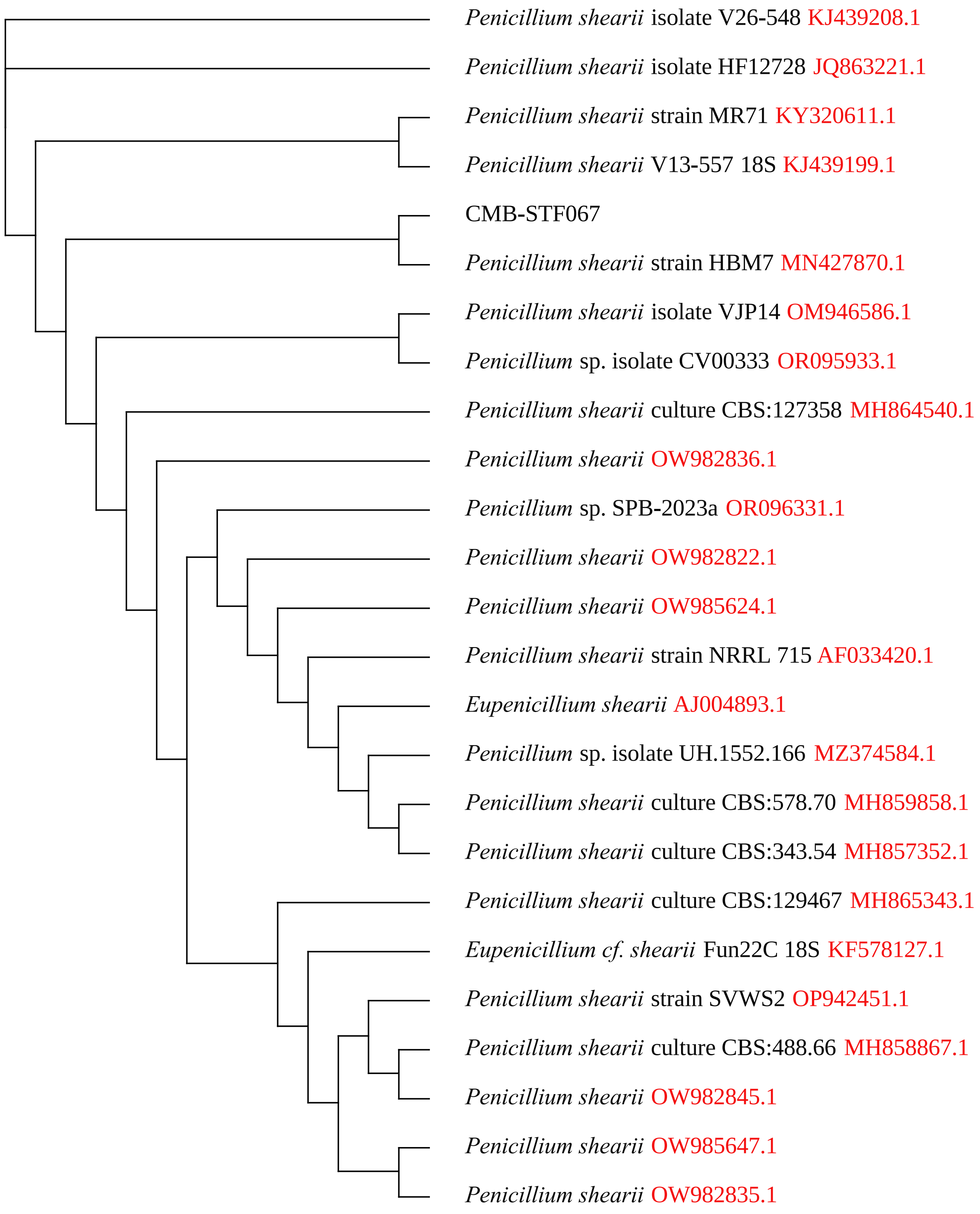

Figure S3. Phylogenetic analysis of CMB-STF067 using PhyML Maximum Likelihood analysis of ITS DNA sequences using the optimal nucleotide substitution model determined by jModeltest2 using UGENE software. Phylogenetic relationship of CMB-STF067 with selected reference strains shown with their accession numbers indicated in red in colour.

1.2. General experimental

Chemicals were purchased from Merck (Darmstadt, Germany) unless otherwise specified. Analytical-grade solvents were used for solvent extractions, while the HPLC grade chromatography solvents were supplied by Labscan (Bangkok, Thailand) and filtered/degassed through 0.45 μm polytetrafluoroethylene (PTFE) membrane prior to use. Water for HPLC was filtered through a Milli-Q® Direct water purification system prior to degassing and further filtration through a 0.45 μm Nylon membrane. Deuterated solvents were purchased from Cambridge Isotopes (Tewksbury, MA, USA). Microorganisms were manipulated under sterile conditions using a Laftech class II biological safety cabinet and incubated in either MMM Friocell incubators (Lomb Scientific, Taren Point, NSW, Aus) or an Innova 42R incubator shaker (John Morris, Chatswood, NSW, Aus). Twenty-four well microbioreactors, cover clamps, and sandwich covers were purchased from Applikon Biotechnology (Getinge AB, Sweden). Analytical UPLC chromatograms were obtained on Agilent 1290 infinity UPLC system (Agilent, Mulgrave, Vic, Aus) composed of 1290 infinity quaternary pump, thermostat, autosampler and diode array detector (DAD) (Agilent Zorbax C_8 1.8 μm, 2.1× 50 mm column, gradient elution at 0.417 mL/min over 2.50 min from 90% H_2O/MeCN to 100% MeCN with an isocratic 0.01% trifluoroacetic acid (TFA)/MeCN modifier). UPLC-QTOF MS/MS analyses were performed on UPLC-QTOF instrument (Agilent, Mulgrave, Vic, Aus) comprising an Agilent 1290 Infinity II UPLC (Zorbax C_8 1.8 μm, 2.1× 50 mm column, gradient elution at 0.417 mL/min over 2.50 min from 90% H_2O/MeCN to 100% MeCN with an isocratic 0.1% formic acid/MeCN modifier) coupled to an Agilent 6545 Q-TOF, mass range 100 – 1700 *m/z* (with collision energy 35 eV). Liquid chromatography-diode array-mass spectrometry (HPLC-DAD-MS) data were acquired on an Agilent 1260 series separation module equipped with an Agilent G6125B series LC/MSD mass detector and diode array detector (Agilent, Mulgrave, Vic, Aus). Preparative and semi-preparative HPLC were performed using Agilent 1100 series LC instruments (Agilent, Mulgrave, Vic, Aus) with corresponding detectors, fraction collectors and software inclusively. Optical rotations ($[\alpha]_D$) were obtained on a JASCO P-1010 polarimeter (Jasco Corporation, Tokyo, Japan) in a 100 × 2 mm cell at the specified temperature. Circular dichroism (CD) spectra were recorded on a JASCO J-810 spectropolarimeter (Jasco Corporation, Tokyo, Japan) in 1 mm quartz cells at room temperature with scanning speed of 100 nm/min and 0.5 nm step scan over the wavelength from 200–600 nm. NMR experiments were performed on Bruker Avance 600 MHz spectrometer (Bruker Pty Ltd, Preston, Vic, Aus), in MeOD or $CDCl_3$ with referencing to residual ^{1}H or ^{13}C resonances (MeOD, δ_H 3.31 and δ_C 49.0; $CDCl_3$, δ_H 7.26 and δ_C 77.23). High-resolution (HR) ESIMS measurements were obtained on a Bruker micrOTOF mass spectrometer (Bruker Pty Ltd, Preston, Vic, Aus) by direct infusion in MeCN at 3 μL/min using sodium formate clusters as an internal calibrant. Molecular modelling was performed using Chem3D 20.0 software (PerkinElmer, Inc., Waltham, MA, USA) with MM2 or MMFF94 energy minimization. The optical density of the microbial cultures was measured using GENESYS 30 Visible Spectrophotometer (Thermo Scientific, Seventeen Mile Rocks, Qld, Aus) with 1 cm quartz cell. Optical density (OD_{600}) was measured spectrophotometrically at 600 nm using POLARstar Omega plate reader (BMG LABTECH, Offenburg, Germany).

1.3. Global Natural Product Social (GNPS) Molecular Networking

(a)

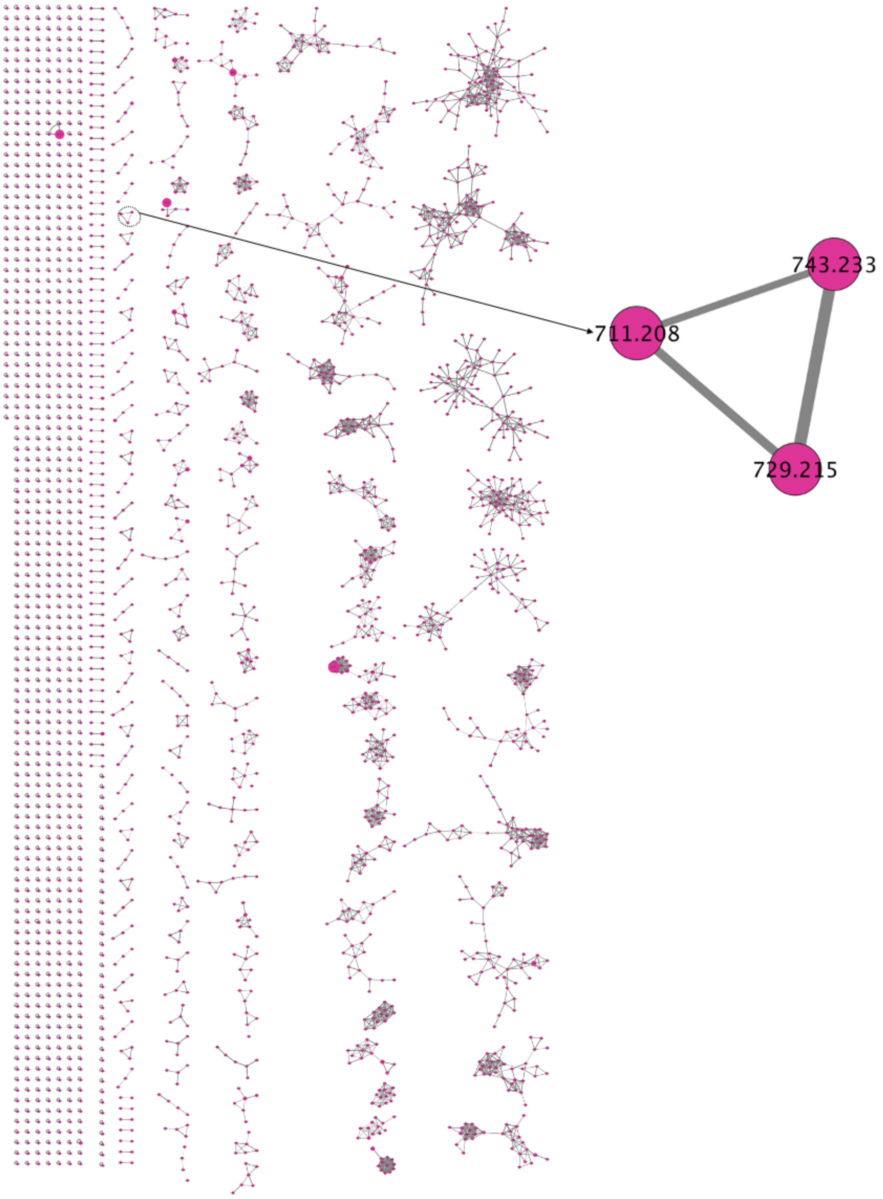

The MS/MS data (.mzXML) for 176 fungal extracts can be accessed from
https://massive.ucsd.edu/ProteoSAFe/dataset.jsp?task=c148b724b8c4499ab8faac1b3dff0399

GNPS molecular networking of 176 fungal extracts can be accessed from
https://gnps.ucsd.edu/ProteoSAFe/status.jsp?task=d18527df6be44fc498f3c700cd4d0643

(b)

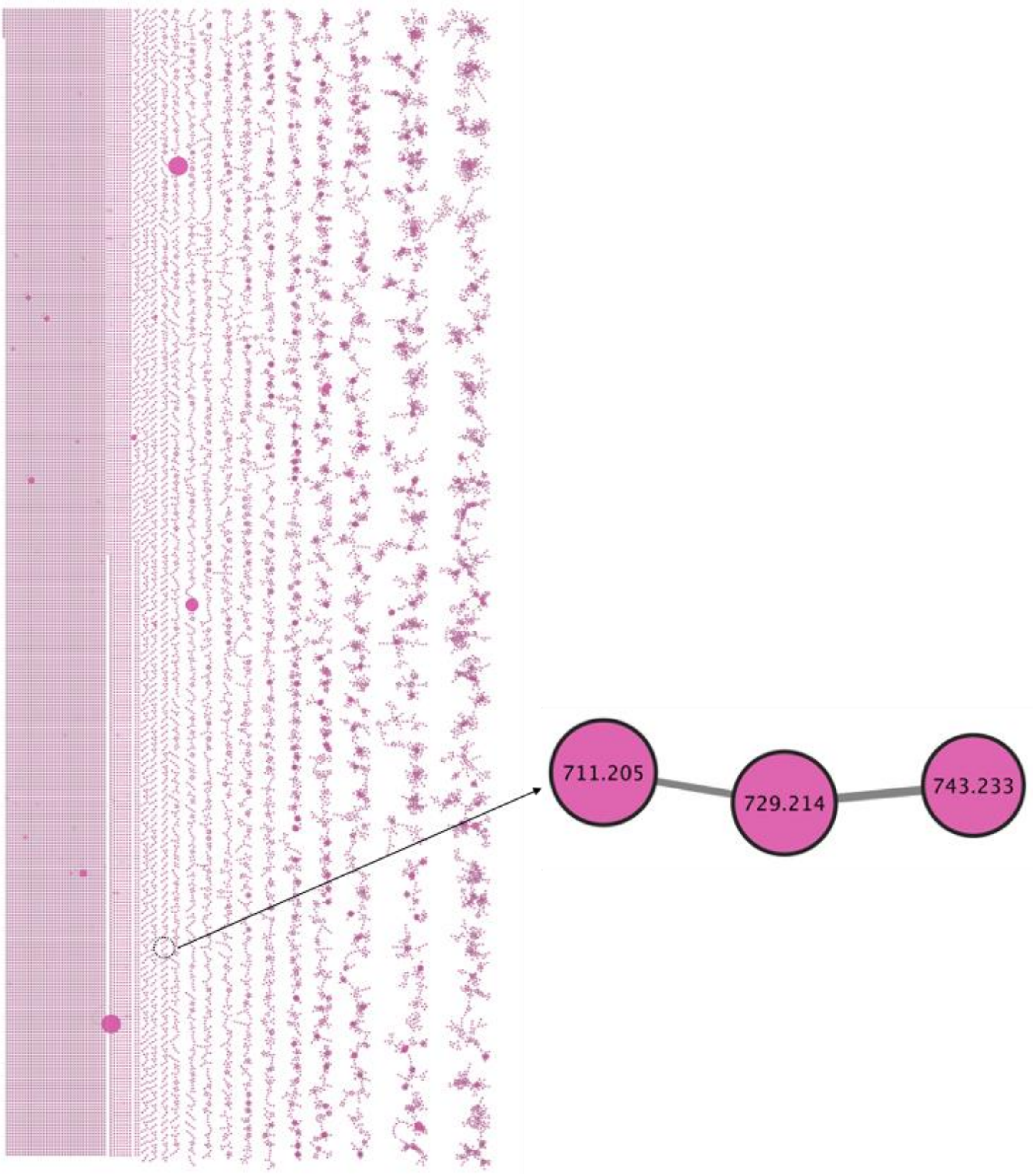

Figure S4. GNPS molecular network of extracts obtained from (a) x176 NSW soil-derived fungi and (b) x1876 Capon lab microbial collection including CMB-STF067. Expanded molecular family for the jugiones, unique to CMB-STF067.

1.4. MATRIX Chemical Profiling

1.4.1. Standard MATRIX

The fungus *Penicillium shearii* CMB-STF067 was subjected to cultivation profiling in a 24-well plate (MATRIX) system using ×11 different media compositions (Table S1) under solid agar (1.5 g), and static and shaken broth (1.5 mL) formats at 27 °C for 10 days. An additional set of control incubations were prepared from ×11 different media solid phase cultivations without inoculation with CMB-STF067. Individual MATRIX wells were extracted *in situ* with EtOAc (2 mL) with individual organic phases dried at 40 °C under a stream of N_2, re-suspended in MeOH (100 mL) containing the HPLC calibrant 1-decyloxy-2,4-dinitrobenzene (50 µg/mL), and centrifuged (13,000 rpm for 3 min) to generate supernatant analytes that were stored in sealed vials at –20 °C prior to UPLC-DAD and UPLC-QTOF analysis.

Table S1. List of media used in MATRIX cultivation profiling of CMB-STF067.

Media		Composition (per litre)
Solid *	Liquid	
PDA	PDB	potato extract (4.0 g), dextrose (20.0 g)
SDA	SDB	peptic digest of animal tissue (5.0 g), pancreatic digest of casein (5.0 g), dextrose (40.0 g)
ISP2	ISP2	yeast extract (4.0 g), malt extract (10.0 g), glucose (4.0 g)
M1	M1	peptone (2.0 g), yeast extract (4.0 g), starch (10.0 g)
M2	M2	mannitol (40.0 g), maltose (40.0 g), yeast extract (10.0 g), K_2HPO_4 (2.0 g), $MgSO_4.7H_2O$ (0.5 g), $FeSO_4.7H_2O$ (0.01 g)
GYA	GYB	yeast extract (4 g), malt extract (10 g), glucose (4 g) $CaCO_3$ (2 g), soluble starch (20 g)
CGA	CGB	glycerol (30 g), casein peptone (2 g), K_2HPO_4 (1 g), NaCl (Chem-Supply) (1 g), $MgSO_4.7H_2O$ (0.5 g), trace element solution (5 mL)*<br>*trace element solution: $CaCl_2.2H_2O$ (3 g), $FeC_6O_7H_5$ (1g), $MnSO_4$ (0.2 g), $ZnCl_2$ (0.1g), $CuSO_4.5H_2O$ (0.025 g), $Na_2B_4O_7.10H_2O$ (0.02 g), $CoCl_2$ (0.004 g), $Na_2MoO_4.2H_2O$ (0.01 g)
IMA	IMB	yeast extract (3 g), peptone (5 g), malt extract (3 g), glucose (10 g), sucrose (170 g)
YES	YES	sucrose (150 g), yeast extract (20 g), $MgSO_4.7H_2O$ (0.5 g), $ZnSO_4.7H_2O$ (0.01 g), $CuSO_4.5H_2O$ (0.005 g)
YEME	YEME	yeast extract (4 g), malt extract (10 g), glucose (4 g), mannitol (40 g)
ISP4	ISP4	soluble starch (10.0 g), $CaCO_3$ (2.0 g), $(NH_4)2SO_4$ (2.0 g), K_2HPO_4 (1.0 g), $MgSO_4.7H_2O$ (1.0 g), NaCl (1.0 g), $FeSO_4.7H_2O$ (1 mg), $MnCl_2.7H_2O$ (1.0 mg), $ZnSO_4.7H_2O$ (1.0 mg)

* All solid-based media contain agar @ 20 g/L

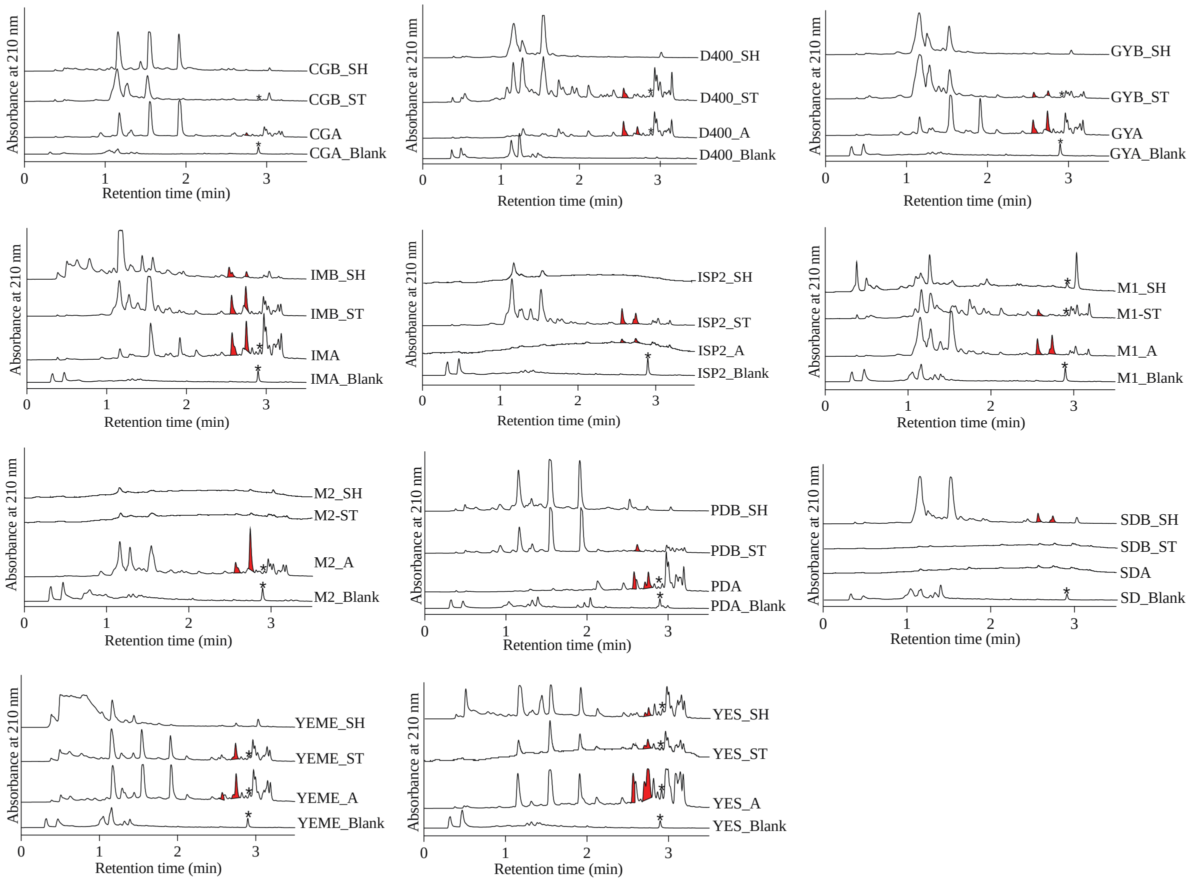

Figure S5. UPLC-DAD (210 nm) traces of CMB-STF067 cultured in different MATRIX culture conditions, with media blanks and an internal calibrant (*). Red highlighted peaks: jugiones A-D.

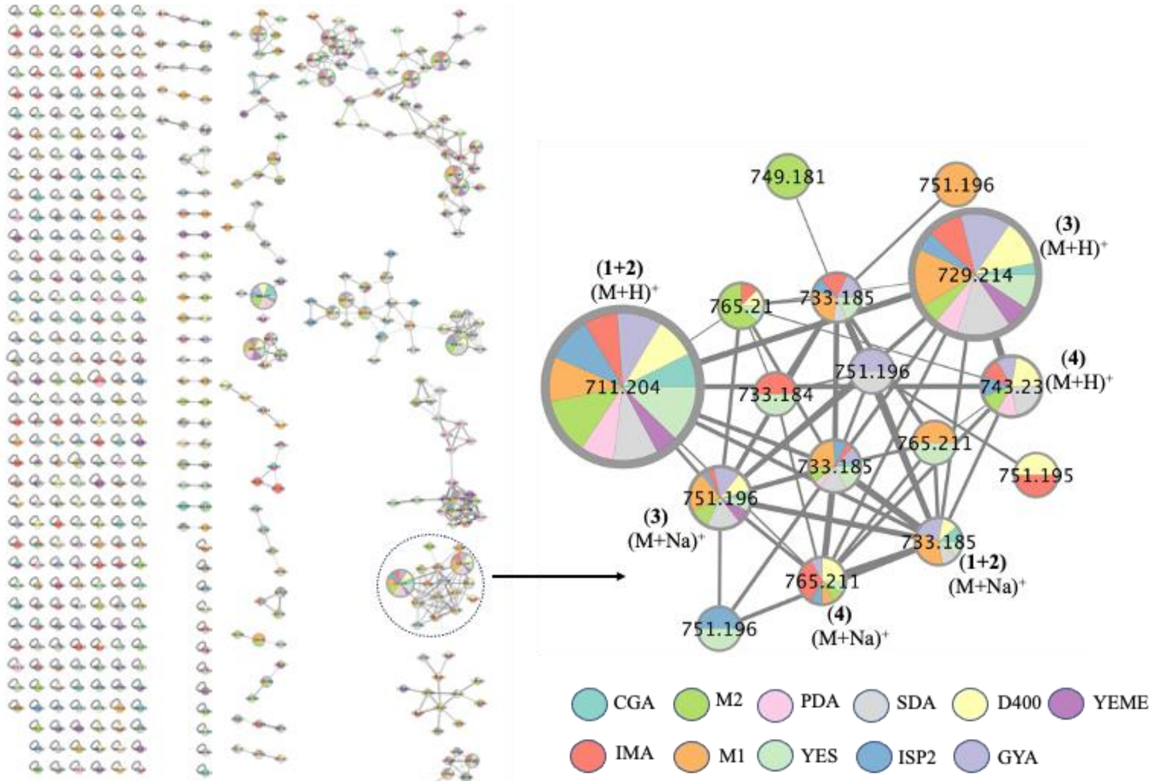

Figure S6. GNPS molecular network of CMB-STF067 in the selected MATRIX media, with expanded view of the jugione molecular family.

1.4.2. Grain MATRIX

The fungus *Penicillium shearii* CMB-STF067 was subjected to profiling in a 24-well plate (grain MATRIX) system on ×23 different grain/pulse solid phase (1.5 g) cultivations (Table S2) at 29 °C for 10 and 21 days. An additional set of control incubations were prepared from ×23 different grains/pulses solid phase cultivations without inoculation with CMB-STF067. Individual MATRIX wells were extracted with EtOAc (2 mL) for each 10 and 21 day cultivation, then dried at 40 °C under a stream of N_2, re-suspended in MeOH (100 mL) containing the HPLC calibrant caffeine (0.125 mg/mL), and centrifuged (13,000 rpm for 3 min) to generate supernatant analytes that were stored in sealed vials at –20 °C prior to UPLC-DAD and UPLC-QTOF analysis.

Table S2.: List of grains/pulses used for grain MATRIX profiling

Grains/pulses	Composition (per well)
brown rice	brown rice (1.0 g), peptone (0.0045 g), yeast extract (0.0045 g), monosodium glutamate (0.0015 g) and 1.5 mL distilled water
red rice	red rice (1.0 g), peptone (0.0045 g), yeast extract (0.0045 g), monosodium glutamate (0.0015 g) and 1.5 mL distilled water
black rice	black rice (1.0 g), peptone (0.0045 g), yeast extract (0.0045 g), monosodium glutamate (0.0015 g) and 1.5 mL distilled water
jasmine rice	jasmine rice (1.0 g), peptone (0.0045 g), yeast extract (0.0045 g), monosodium glutamate (0.0015 g) and 1.5 mL distilled water
Arborio rice (short grain)	Arborio rice (1.0 g), peptone (0.0045 g), yeast extract (0.0045 g), monosodium glutamate (0.0015 g) and 1.5 mL distilled water
Basmati rice	Basmati rice (1.0 g), peptone (0.0045 g), yeast extract (0.0045 g), monosodium glutamate (0.0015 g) and 1.5 mL distilled water
medium grain	medium rice (1.0 g), peptone (0.0045 g), yeast extract (0.0045 g), monosodium glutamate (0.0015 g) and 1.5 mL distilled water
barley	barley (1.0 g), peptone (0.0045 g), yeast extract (0.0045 g), monosodium glutamate (0.0015 g) and 1.5 mL distilled water
couscous (wheat)	couscous (1.0 g), peptone (0.0045 g), yeast extract (0.0045 g), monosodium glutamate (0.0015 g) and 1.5 mL distilled water
rice flour	rice flour (1.0 g), peptone (0.0045 g), yeast extract (0.0045 g), monosodium glutamate (0.0015 g) and 1.5 mL distilled water
noodles	noodles (1.0 g), peptone (0.0045 g), yeast extract (0.0045 g), monosodium glutamate (0.0015 g) and 1.5 mL distilled water
popping corn	popping corn (1.0 g), peptone (0.0045 g), yeast extract (0.0045 g), monosodium glutamate (0.0015 g) and 1.5 mL distilled water
mung bean with shell	mung bean (1.0 g), peptone (0.0045 g), yeast extract (0.0045 g), monosodium glutamate (0.0015 g) and 1.5 mL distilled water
mung bean without shell	mung bean (1.0 g), peptone (0.0045 g), yeast extract (0.0045 g), monosodium glutamate (0.0015 g) and 1.5 mL distilled water
black gram with shell	black gram (1.0 g), peptone (0.0045 g), yeast extract (0.0045 g), monosodium glutamate (0.0015 g) and 1.5 mL distilled water
black gram without shell	black gram (1.0 g), peptone (0.0045 g), yeast extract (0.0045 g), monosodium glutamate (0.0015 g) and 1.5 mL distilled water
sago	sago (1.0 g), peptone (0.0045 g), yeast extract (0.0045 g), monosodium glutamate (0.0015 g) and 1.5 mL distilled water
semolina (wheat)	semolina (1.0 g), peptone (0.0045 g), yeast extract (0.0045 g), monosodium glutamate (0.0015 g) and 1.5 mL distilled water
rice flakes	rice flakes (1.0 g), peptone (0.0045 g), yeast extract (0.0045 g), monosodium glutamate (0.0015 g) and 1.5 mL distilled water
polenta	polenta (1.0 g), peptone (0.0045 g), yeast extract (0.0045 g), monosodium glutamate (0.0015 g) and 1.5 mL distilled water
wholemeal flour	whole meal flour (1.0 g), peptone (0.0045 g), yeast extract (0.0045 g), monosodium glutamate (0.0015 g) and 1.5 mL distilled water
Sri Lanka red rice	Sri Lankan red rice (1.0 g), peptone (0.0045 g), yeast extract (0.0045 g), monosodium glutamate (0.0015 g) and 1.5 mL distilled water
lentils	lentils (1.0 g), peptone (0.0045 g), yeast extract (0.0045 g), monosodium glutamate (0.0015 g) and 1.5 mL distilled water

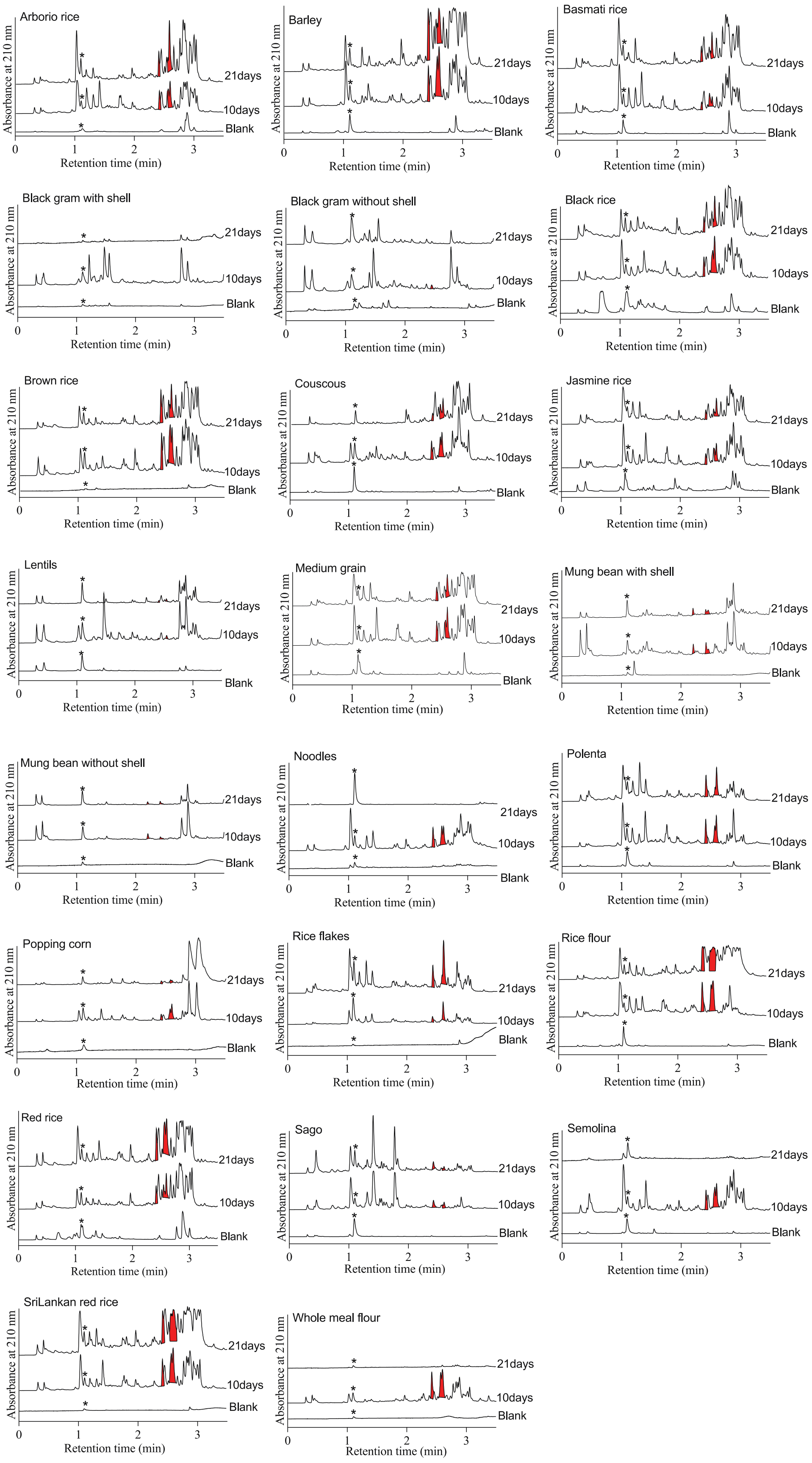

Figure S7. UPLC-DAD (210 nm) traces of CMB-STF067 cultured in different grain MATRIX culture conditions, with media blanks and an internal calibrant (*). Red highlighted peaks: jugiones A-D.

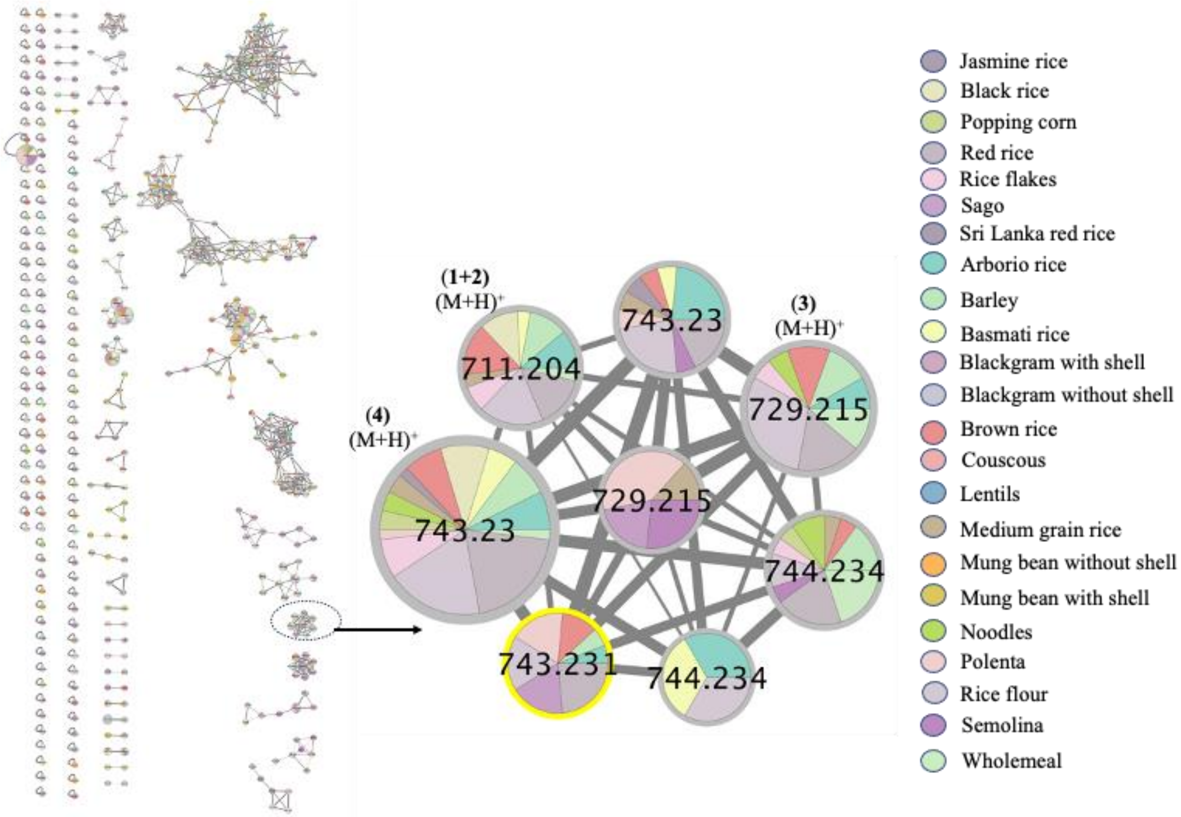

Figure S8. GNPS molecular network of the grain MATRIX cultivation profile of CMB-STF067 with an expanded view of the jugione molecular family.

1.4.3. Cereal MATRIX

The fungus *Penicillium shearii* CMB-STF067 was subjected to profiling in a 24-well plate (cereal MATRIX) system on ×11 different cereal solid phase (1.5 g) cultivations (Table S3) at 29 °C for 10 and 21 days. An additional set of control incubations were prepared from ×11 different cereal solid phase cultivations without innoculation with CMB-SWF012. Individual MATRIX wells were extracted with EtOAc (2 mL) for each 10 and 21 day cultivation, then dried at 40 °C under a stream of N_2, re-suspended in MeOH (100 mL) containing the HPLC calibrant caffeine (0.125 mg/mL), and centrifuged (13,000 rpm for 3 min) to generate supernatant analytes that were stored in sealed vials at –20 °C prior to UPLC-DAD and UPLC-QTOF analysis.

Table S3: List of breakfast cereals used for cereal MATRIX

Breakfast cereals	Composition (per well)
Corn flakes	Corn flakes (1.5 g), sterile water (2 mL)
Rice puffs	Rice puff (1.5 g), sterile water (2 mL)
Weet-Bix	Weet-Bix (1.5 g), sterile water (2 mL)
Wheat biscuits (Digestive)	Wheat biscuits (1.5 g), sterile water (2 mL)
Rice cakes	Rice cakes (1.5 g), sterile water (2 mL)
Corn thin	Corn thin (1.5 g), sterile water (2 mL)
Cheerios (mixed cereal)	Cheerios (1.5 g), sterile water (2 mL)
Special K	Special K (1.5 g), sterile water (2 mL)
Cruskit (Wafer)	Cruskit (1.5 g), sterile water (2 mL)
Rice cracker	Rice cracker (1.5 g), sterile water (2 mL)
Deb potato	Deb potato (1.5 g), sterile water (2 mL)

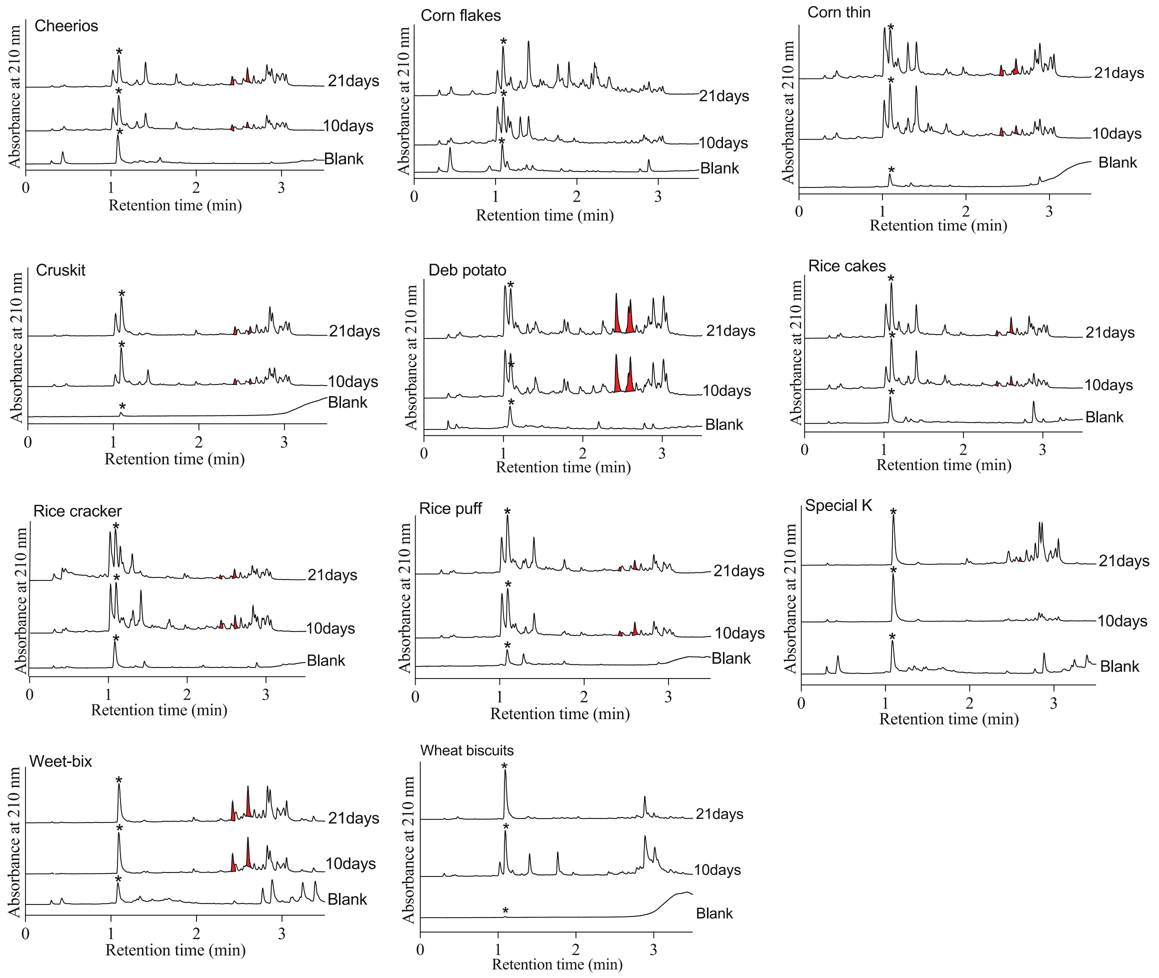

Figure S9. UPLC-DAD (210 nm) traces of CMB-STF067 cultured in different cereal MATRIX culture conditions, with media blanks and an internal calibrant (*). Red highlighted peaks: jugiones A-D.

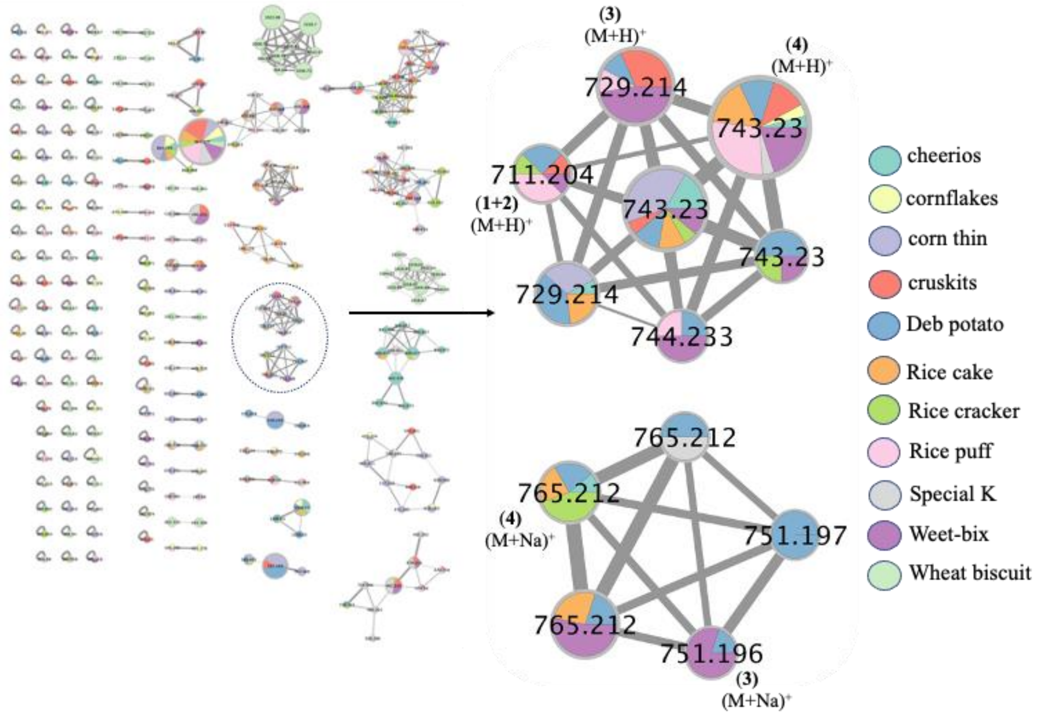

Figure S10. GNPS molecular network of the cereal MATRIX cultivation profile of CMB-STF067 with an expanded view of the jugione molecular family.

1.5. Fractionation of jugiones A–D

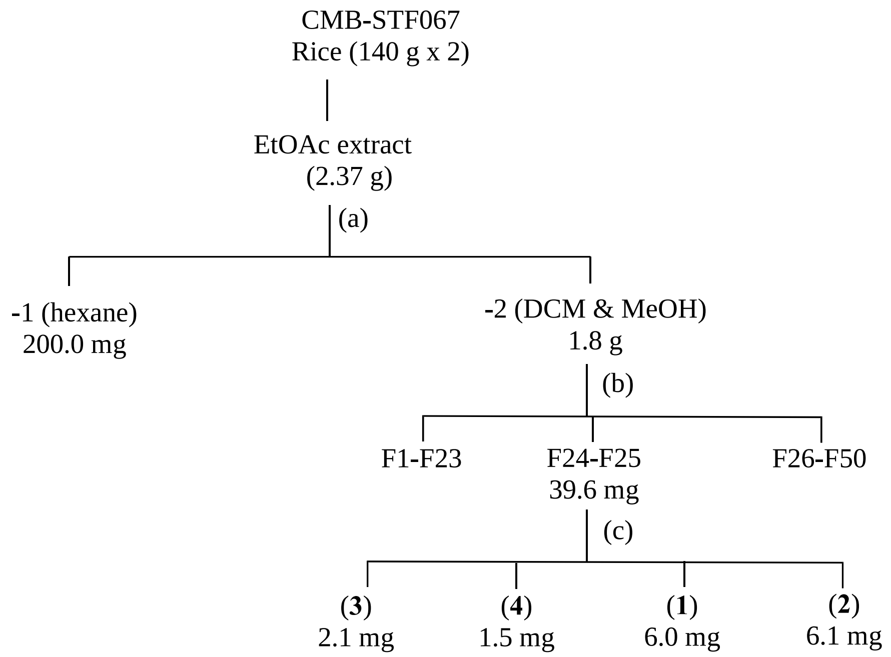

Scheme S1. Isolation scheme for CMB-STF067 cultured in red rice and extracted by EtOAc. (a) solvent trituration (b) Sephadex LH-20 (DCM/MeOH) chromatography, and (c) semi-preparative HPLC.

2. Spectroscopic characterization of jugiones A–D (1-4)

2.1. Jugione A (1)

Table S4. NMR (600 MHz, $CDCl_3$) data for jugione A (**1**)

Position	δ_C	δ_H, mult, (*J* in Hz)	COSY	HMBC	ROESY
1	161.9	-			
2	119.5	6.97, dd (*8.6, 0.9*)	3	1, 4, 6, 7	3, 1-OH
3	136.3	7.45, dd (*7.5, 8.6*)	2, 4	1, 5	2, 4
4	122.2	7.09, dd (*7.5, 0.9*)	3	2, 6, 7, 10	3, 10
5	137.2	-			
6	115.2[A]	-			
7	186.7	-			
8	106.6	-			
9	41.5	-			
10	72.8	6.05, s	-	4, 5, 6, 8, 9, 15, 1′	4, 14, 15a,15b
11	185.8	-			
12	43.4	4.52, d (*1.5*)	14	8, 11, 13, 14, 16, 17, 18, 29	29
13	141.4	-			
14	125.9	5.68, dq (*1.5, 1.5*)	12, 29	8, 9, 10, 12, 29	10, 15a, 29
15	35.9	*a.* 2.77, d (*17.8*)	15b	8, 9, 10, 14, 16, 17, 21	10, 14, 21
		b. 2.68, d (*17.8*)	15a	8, 9, 10, 14, 16, 17, 21	10, 21
16	148.7	-			
17	115.3[A]	-			
18	154.1	-			
19	105.4	-			
20	160.4	-			
21	115.2[A]	6.17, s	-	15, 17, 19, 20, 22	15a, 15b, 20-OH
22	188.0	-			
23	99.8	-			
24	84.3	-			
25	68.9	4.59, dd (*4.4, 1.8*)	26a, 26b	23, 24, 27	26a, 26b, OMe
26	32.4	*a.* 2.62, ddd (*14.1, 6.7, 4.4*)	25, 26b,	24, 25, 27	25, 27
		b. 1.98, ddd (*14.1, 10.2, 1.8*)	25, 26a,	27	25, OMe
27	64.1	4.81, dd (*10.2, 6.7*)	26a, 26b	23, 26, 28	26a
28	176.8				
29	21.0	1.86, d (*1.5*)	14	12, 13, 14	12, 14
30	170.5	-			
1′	166.7	-			
2′	119.2	5.75, d (*13.9*)	3′	1′, 4′	
3′	146.5	7.24, dd (*13.9, 11.2*)	2′, 4′	1′, 5′	4′, 5′
4′	127.5	6.12, dd (*14.8, 11.2*)	3′, 5′	6′	3′
5′	142.2	6.52, dd (*14.8, 10.8*)	4′, 6′	3′, 6′, 7′	3′
6′	131.4	6.12, dd (*15.0, 10.8*)	5′, 7′, 8′	4′, 8′	8′
7′	136.1	5.95, dq (*15.0, 6.8*)	6′, 8′	5′, 8′	8′
8′	18.8	1.82, d (*6.8*)	6′, 7′	6′, 7′	6′, 7′
OMe	54.0	3.75, s		30	25, 26b
1-OH	-	11.58, s		1, 2, 6	2
11-OH	-	14.21[B], br s			
20-OH	-	11.05, s		19, 20, 21	21
28-OH	-	14.06[B], br s			

[A–B] assignments within the same column are interchangeable.

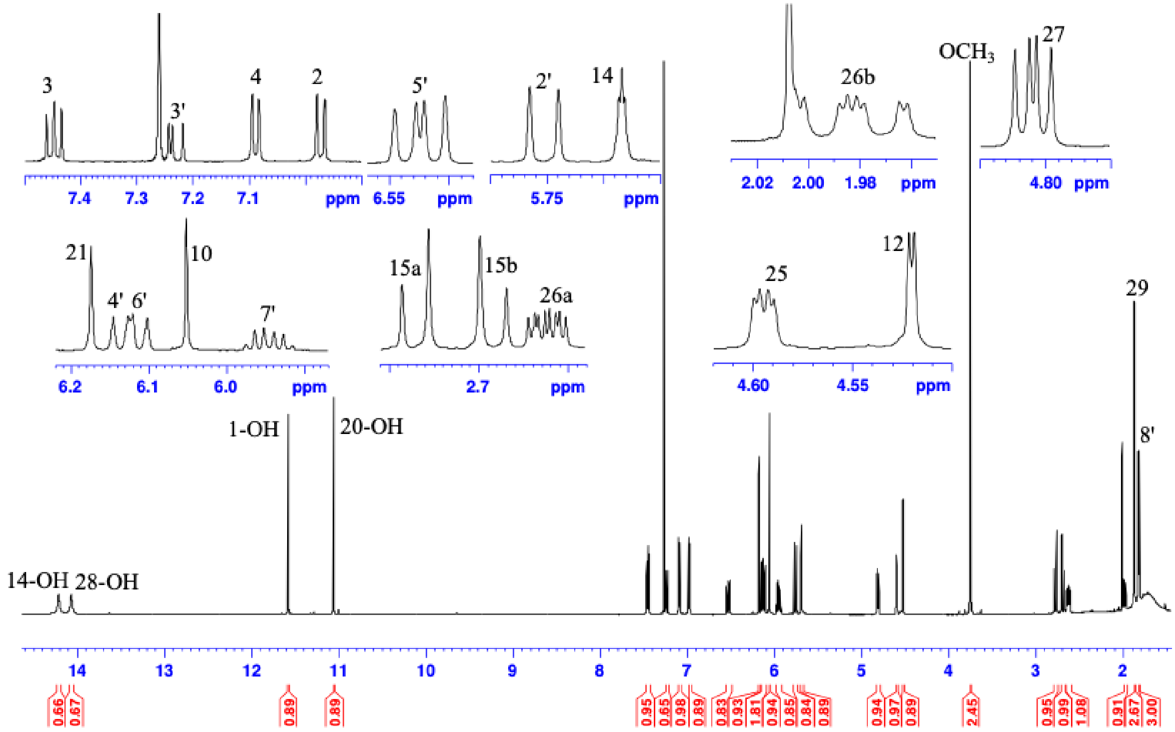

Figure S11. ^{1}H NMR (600 MHz, $CDCl_3$) spectrum of jugione A (**1**)

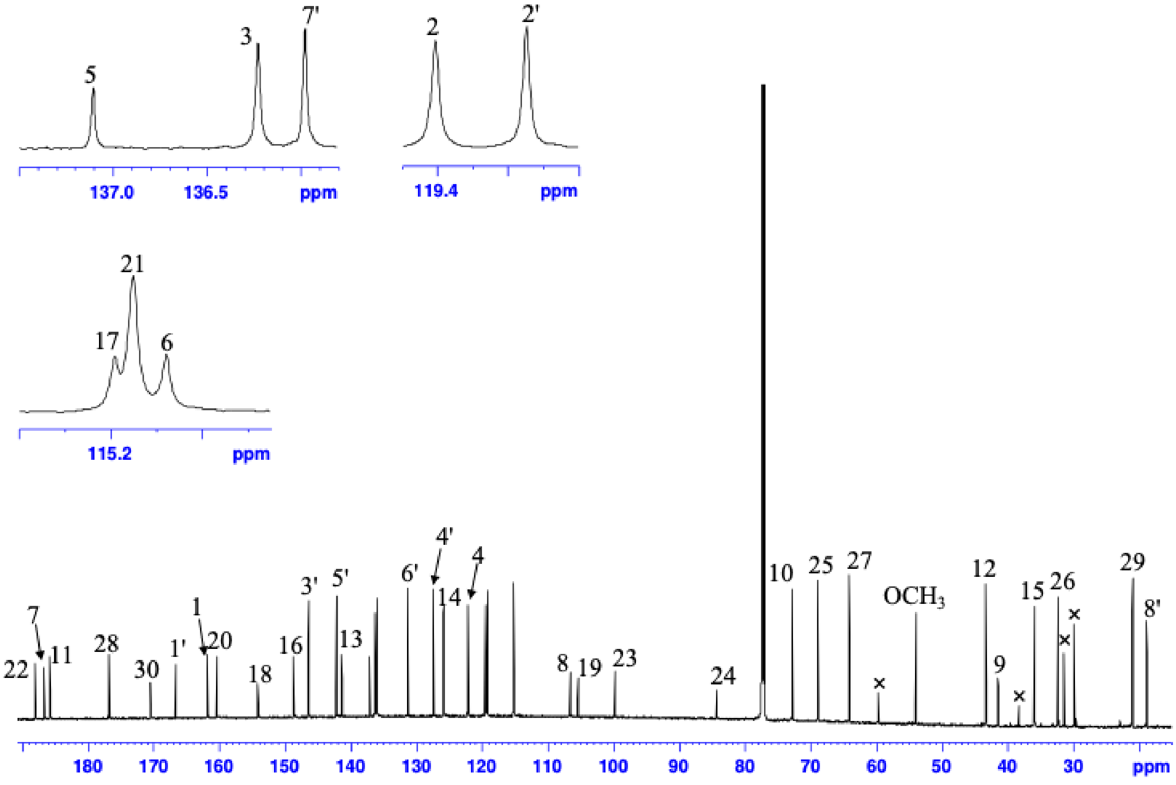

Figure S12. ^{13}C NMR (150 MHz, $CDCl_3$) spectrum of jugione A (**1**)

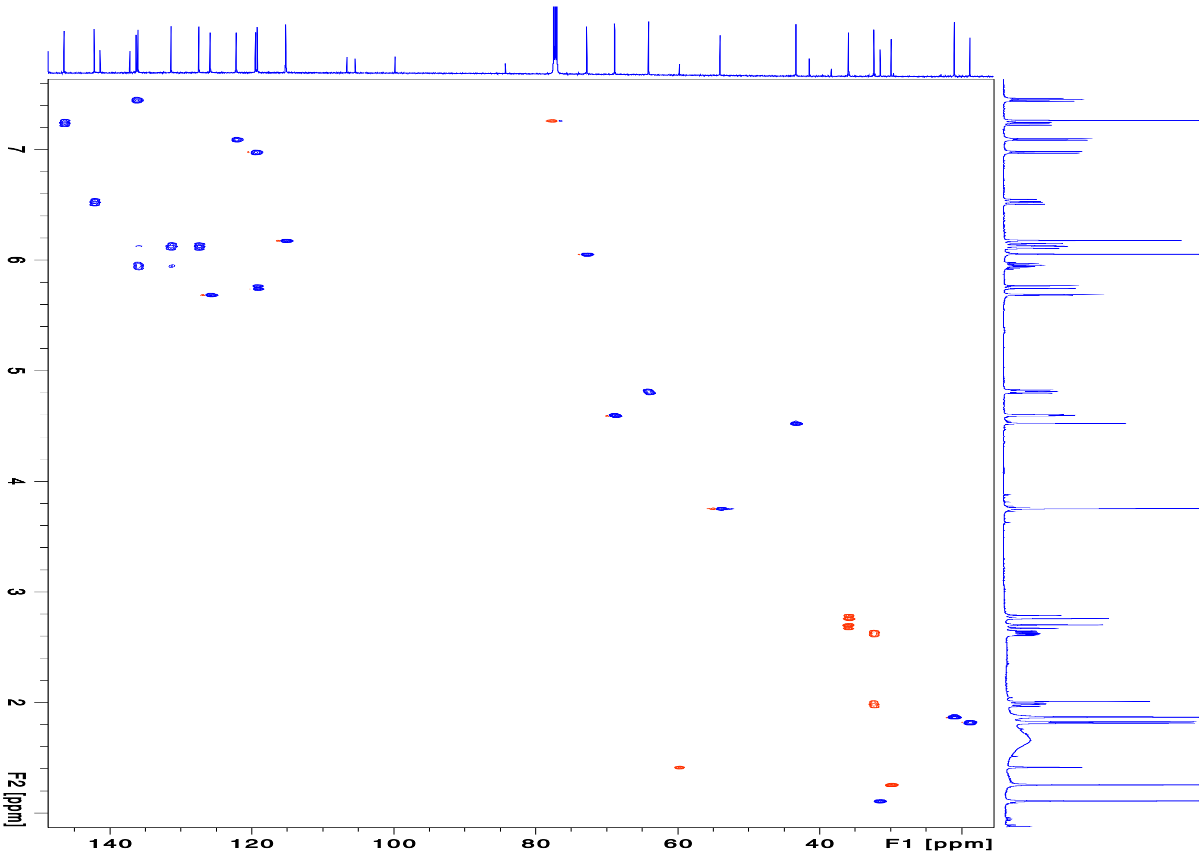

Figure S13. HSQC NMR (600 MHz, $CDCl_3$) spectrum of jugione A (**1**)

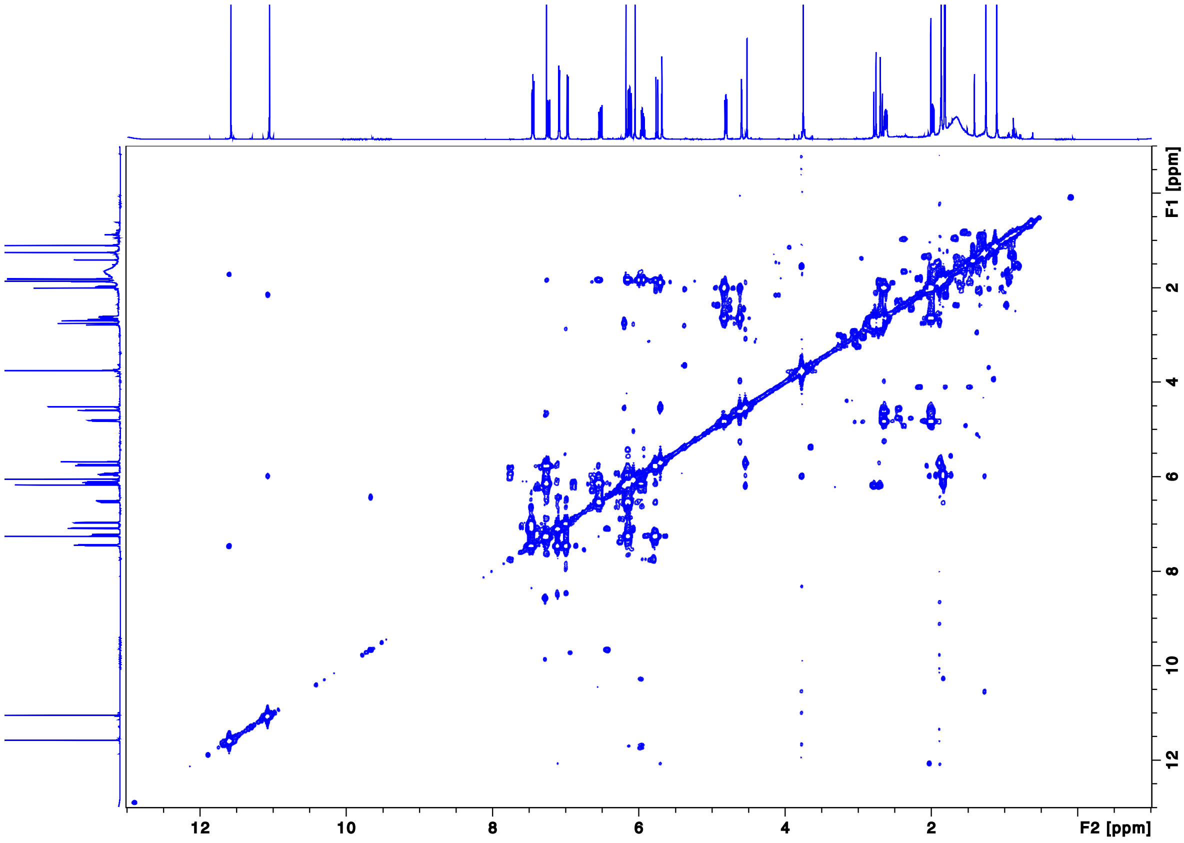

Figure S14. COSY NMR (600 MHz, $CDCl_3$) spectrum of jugione A (**1**)

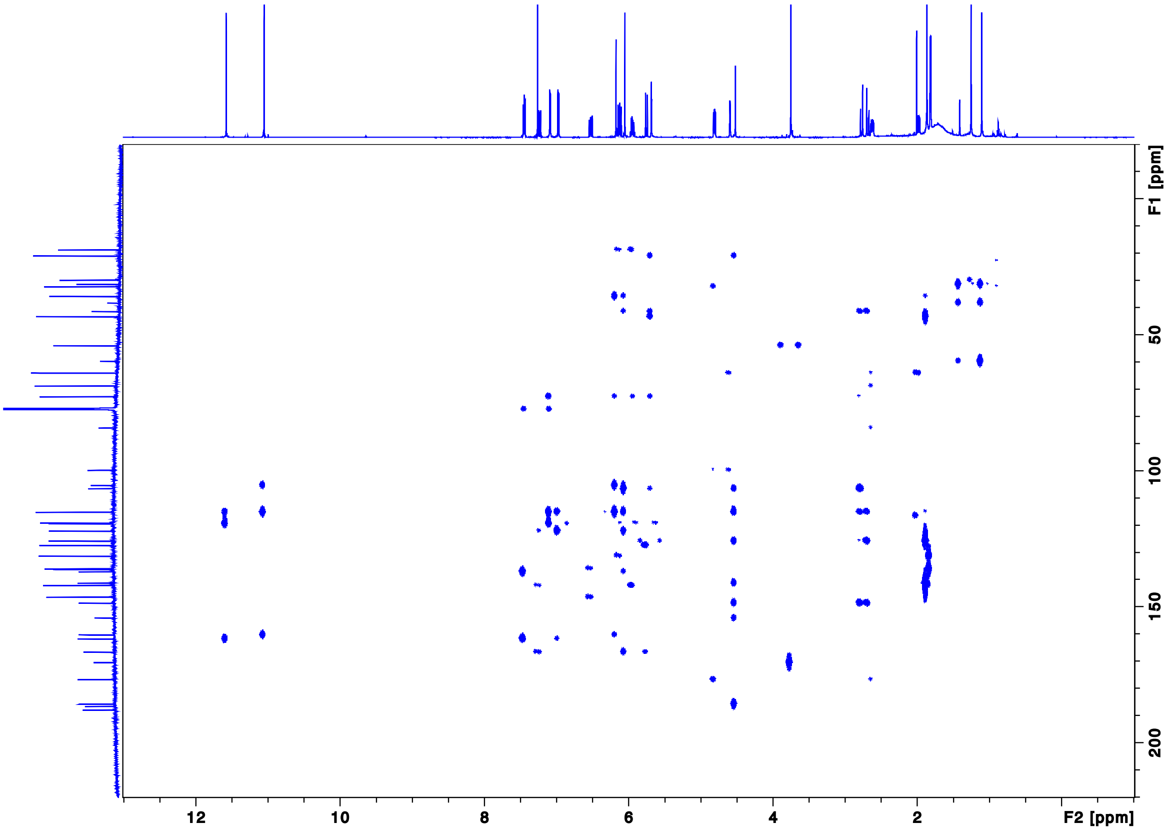

Figure S15. HMBC NMR (600 MHz, $CDCl_3$) spectrum of jugione A (**1**)

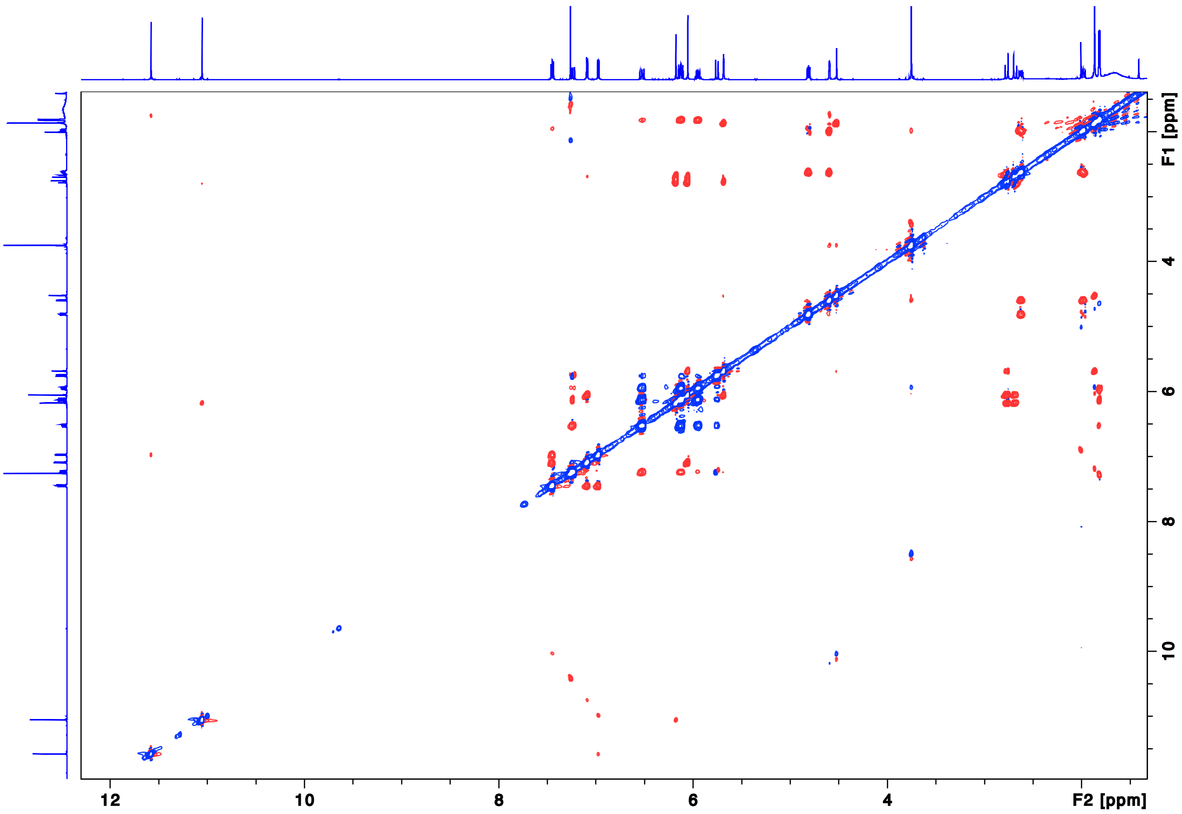

Figure S16. ROESY NMR (600 MHz, $CDCl_3$) spectrum of jugione A (**1**)

Mass Spectrum Molecular Formula Report

Analysis Info

Analysis Name	D:\Data\Thulasi\CMB-STF067_M2_SPE24_21_711.d	Acquisition Date	5/16/2023 11:47:14 AM
Method	tune-medhigh_AP.m	Operator	a.salim
Sample Name	CMB-STF067_M2_SPE24_21_711	Instrument / Ser#	micrOTOF 213750.00232
Comment			

Acquisition Parameter

Source Type	ESI	Ion Polarity	Positive	Set Nebulizer	0.5 Bar
Focus	Not active			Set Dry Heater	180 °C
Scan Begin	100 m/z	Set Capillary	4500 V	Set Dry Gas	5.0 l/min
Scan End	1500 m/z	Set End Plate Offset	-500 V	Set Divert Valve	Source

Generate Molecular Formula Parameter

Formula, min.		
Formula, max.		
Measured m/z	Tolerance	Charge
Check Valence	Minimum	Maximum
Nirogen Rule	Electron Configuration	
Filter H/C Ratio	Minimum	Maximum
Estimate Carbon		

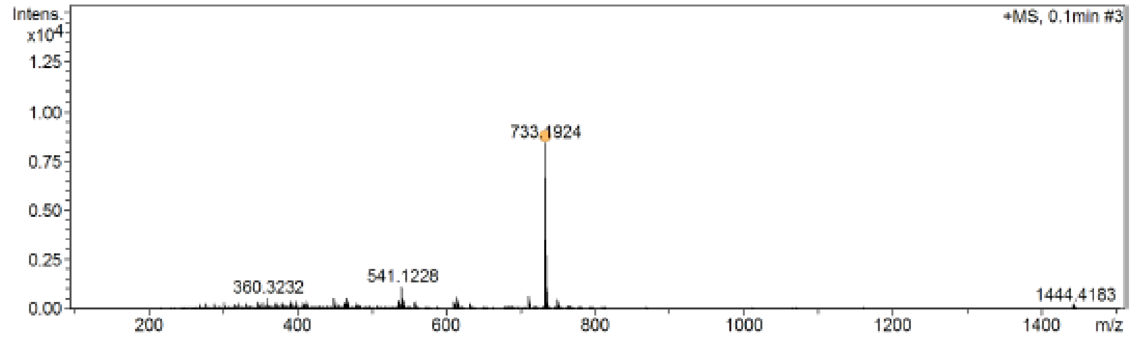

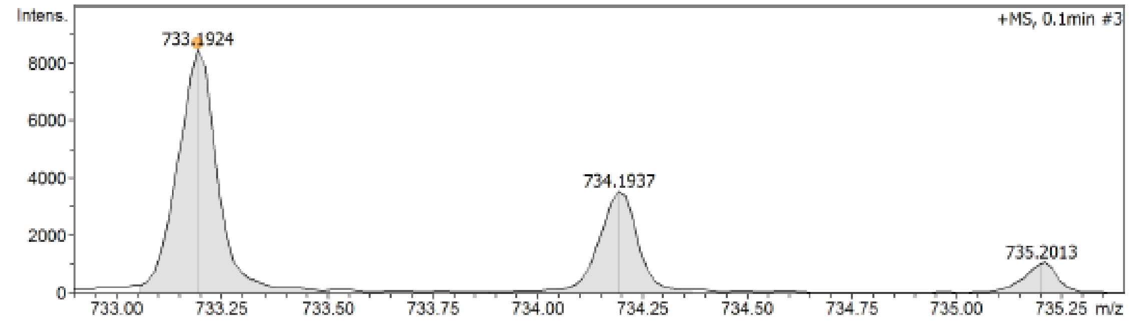

Meas. m/z	#	Ion Formula	m/z	err [ppm]	mSigma	# Sigma	Score	rdb	e⁻ Conf	N-Rule
733.1924	1	C39H34NaO13	733.1892	-4.5	8.0	1	21.51	22.5	even	ok
	2	C35H30N6NaO11	733.1865	8.1	12.5	2	0.56	23.5	even	ok
	3	C36H26N10NaO7	733.1878	6.3	13.6	3	3.95	28.5	even	ok
	4	C35H26N12NaO6	733.1990	-9.0	14.4	4	0.18	28.5	even	ok
	5	C34H30N8NaO10	733.1977	-7.2	14.9	5	1.59	23.5	even	ok
	6	C40H30N4NaO9	733.1905	2.7	17.4	6	58.86	27.5	even	ok
	7	C32H22N16NaO5	733.1851	-10.0	18.5	7	0.04	29.5	even	ok
	8	C33H18N20NaO	733.1865	8.2	19.5	8	0.48	34.5	even	ok

Bruker Compass DataAnalysis 4.1 printed: 5/16/2023 12:18:47 PM Page 1 of 2

Figure S17. HRMS spectrum and measurement for jugione A (**1**)

Table S5. Comparison of NMR data ($CDCl_3$) for JBIR-99 (**5**), engyodontochone B (**6**) and xanthoquinodins B10-B11 (**7**-**8**) with jugione A (**1**)

Pos.	JBIR-99 (**5**)[12]		engyodontochone B (**6**)[12]		xanthoquinodin B10 (**7**)[13]		xanthoquinodin B11 (**8**)[13]		jugione A (**1**)	
	δ_C	δ_H, mult, (*J* in Hz)	δ_C	δ_H, mult, (*J* in Hz)	δ_C	δ_H, mult, (*J* in Hz)	δ_C	δ_H, mult, (*J* in Hz)	δ_C	δ_H, mult, (*J* in Hz)
1	161.5		161.3		161.6		161.7		161.9	-
2	119.4	6.98, d (*8.4*)	119.4	6.96, d (*8.3*)	119.3	6.80, s	119.3	6.80, s	119.5	6.98, dd (*8.6, 0.9*)
3	135.9	7.45, t (*8.4*)	135.8	7.44, t (*8.3*)	147.7		147.8		136.3	7.45, dd (*7.5, 8.6*)
4	121.9	7.06, d (*8.4*)	122.0	7.05, d (*8.3*)	123.2	6.90, s	123.2	6.90, s	122.2	7.09, dd (*7.5, 0.9*)
5	136.7		136.6		136.5		136.6		137.2	-
6	114.9		114.6		112.4		112.6		115.2	-
7	185.8		184.5		185.3		185.2		186.7	-
8	106.4		105.8		105.6		105.7		106.6	-
9	41.2		41.1		41.6		41.4		41.5	-
10	73.1	5.96, s	73.0	5.95, s	72.7	5.98, s	72.7	5.98, s	72.8	6.05, s
11	186.4		186.9		186.1		186.4		185.8	-
12	43.3	4.55, d (*1.5*)	43.6	4.54, d (*1.5*)	38.6	4.81, dd (*6.7,0.7*)	37.9	4.76, dd (*6.7, 1.0*)	43.4	4.52, d (*1.5*)
13	141.8		141.4		131.8	6.48, dd (*8.4, 6.7*)	131.6	6.41, dd (*8.4, 6.7*)	141.4	-
14	125.2	5.64, s	125.6	5.70, s	132.3	6.08, dd (*8.4, 0.7*)	132.7	6.07, dd (*8.4, 1.0*)	125.9	5.68, dd (*1.5, 1.5*)
15	35.7	2.75, d (*17.7*)<br>2.67, d (*17.7*)	35.6	2.78, d (*17.6*)<br>2.62, d (*17.6*)	35.0	2.79, d (*18.0*)<br>2.67, d (*18.0*)	35.0	2.78, d (*17.9*)<br>2.68, d (*17.9*)	35.9	2.77, d (*17.8*)<br>2.68, d (*17.8*)
16	147.9		147.6		147.6		147.3		148.7	-
17	114.7		114.6		115.3		115.2		115.3	-
18	154.9		154.2		154.8		153.6		154.1	-
19	105.2		105.1		105.4		105.3		105.4	-
20	160.2		160.3		160.1		160.2		160.4	-
21	114.2	6.12, s	114.4	6.13, s	114.2	6.13, s	114.7	6.15, s	115.2	6.17, s
22	186.8		187.6		186.7		186.9		188.0	-
23	101.3		100.1		101.6		100.2		99.8	-
24	85.1		84.5		85.3		84.5		84.3	-
25	72.0	3.39, dd (*12.3, 4.8*)	66.9	4.43, dd (*3.8, 2.0*)	71.7	4.46, dd (*12.3, 5.0*)	66.9	4.49, dd (*4.0, 2.0*)	68.9	4.60, dd (*4.4, 1.7*)
26	24.1	2.23, m<br>2.12, m	23.3	2.18, m<br>1.94, m	23.9	2.21, m<br>2.10, m	22.9	2,18, m<br>2.00, m	32.4	2.62, m<br>1.98, m
27	27.7	2.68, m	24.5	2.40, dd (*16.2, 5.5*)<br>2.82, ddd (*15.7, 9.4, 5.5*)	27.7	2.69, m	24.4	2.84, m<br>2.44, m	64.1	4.81, dd (*10.3, 6.7*)
28	178.5		179.9		178.5		180.2		176.7	-
29	20.8	1.90, s	20.6	2.00, s	22.1	2.38, s	22.1	2.38, s	21.0	1.86, d (*1.4*)
30	170.2		170.5		169.8		171.0		170.5	-
OMe	53.4	3.72, s	53.5	3.72, s	53.3	3.73, s	53.6	3.74, s	54.0	3.75, s
1′	170.4		171.0		173.1		173.0		166.7	-
2′	21.2	2.00, s	21.2	2.00, s	36.2	2.23, m	36.2	2.22, m	119.2	5.75, d (*15.2*)
3′	-		-		18.4	1.58, m	18.4	1.57, m	146.5	7.24, dd (*13.9, 11.2*)
4′	-		-		13.5	0.86, t (*0.74*)	13.5	0.86, t (*0.74*)	127.5	6.12
5′	-		-		-	-	-	-	142.2	6.52, dd (*14.9, 10.8*)
6′	-		-		-	-	-	-	131.4	6.12
7′	-		-		-	-	-	-	136.1	5.95, dq (*15.2, 7.1*)
8′	-		-		-	-	-	-	18.8	1.82, brd (*6.8*)
1-OH	-		-		-	11.40	-	11.55	-	11.58, s
11-OH	-		-		-	-	-	-	-	14.21, s
20-OH	-		-		-	11.05	-	11.24	-	11.05, s
28-OH	-		-		-	13.95	-	14.10	-	14.07, s
25-OH	-		-		-	14.26	-	-	-	

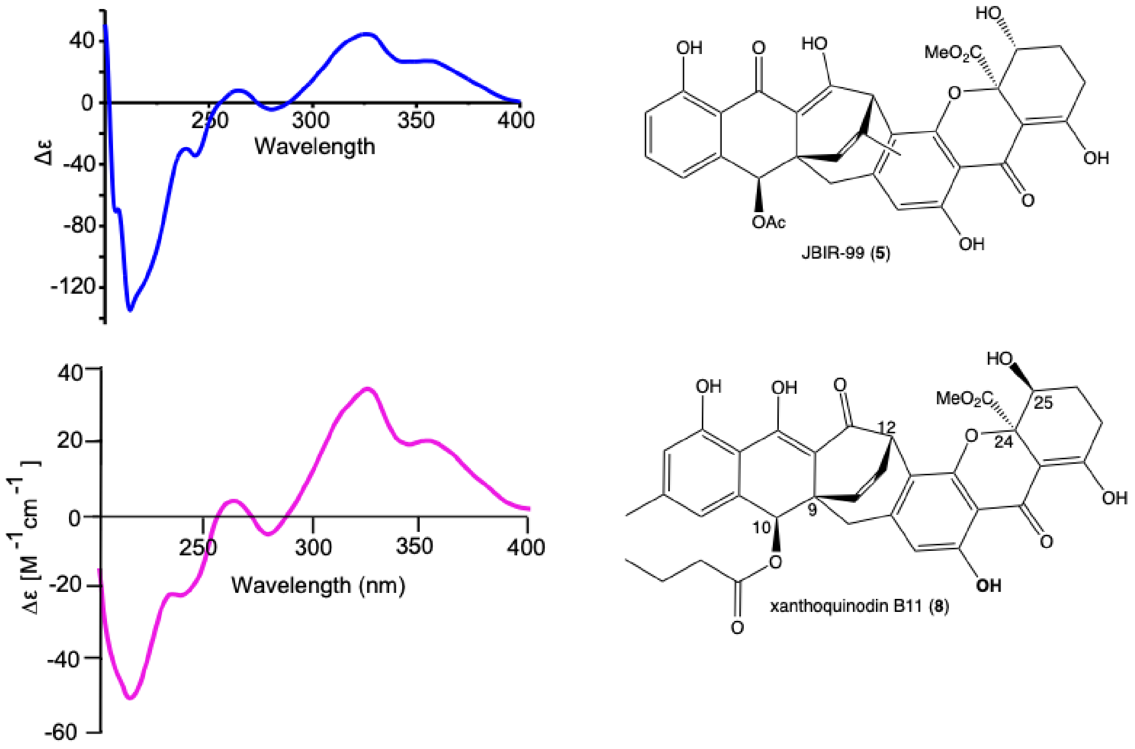

Figure S18. ECD spectra comparison of JBIR-99 (**5**) and xanthoquinodin B11 (**8**). The ECD spectra of these compounds are adapted from the literatures.[12, 13]

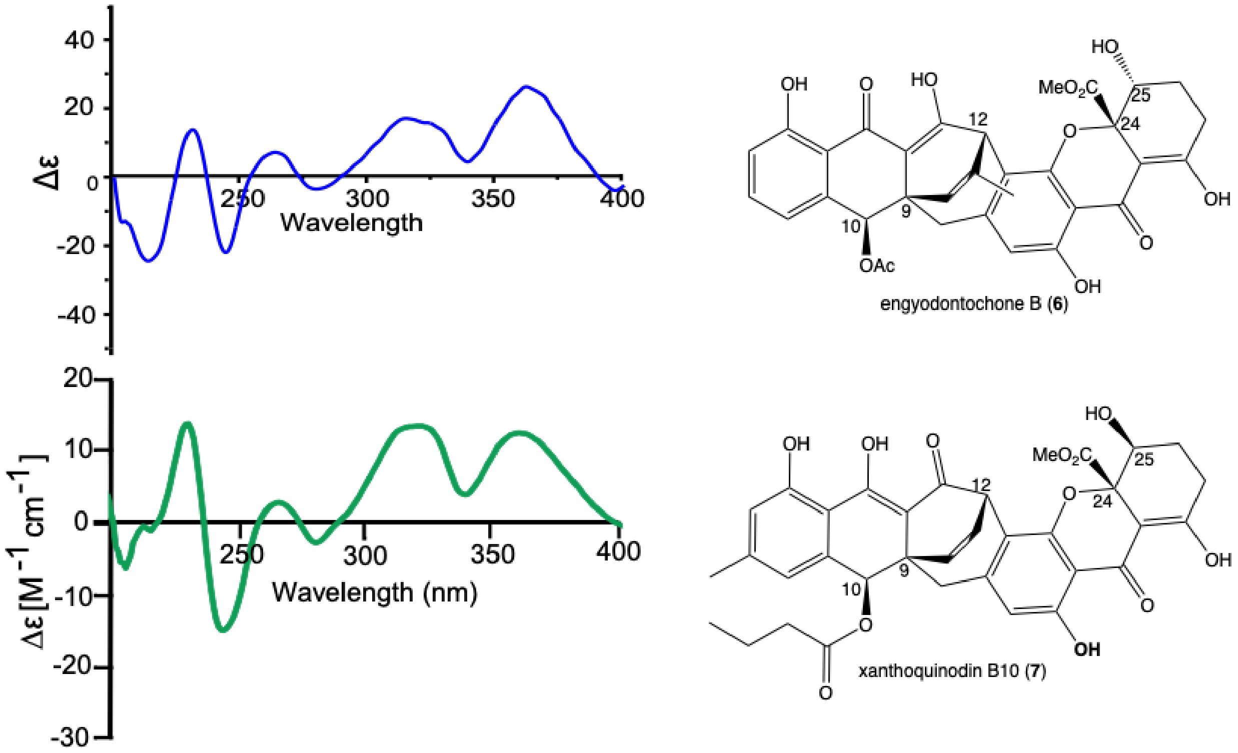

Figure S19. ECD spectra comparison of engyodontochone B (**6**) and xanthoquinodin B10 (**7**). The ECD spectra of these compounds are adapted from the literature.[12, 13]

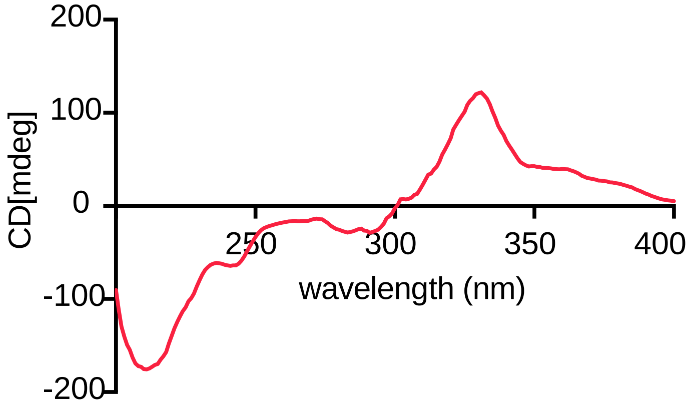

Figure S20. ECD spectra of jugione A (**1**) in MeOH (0.5 mg/mL).

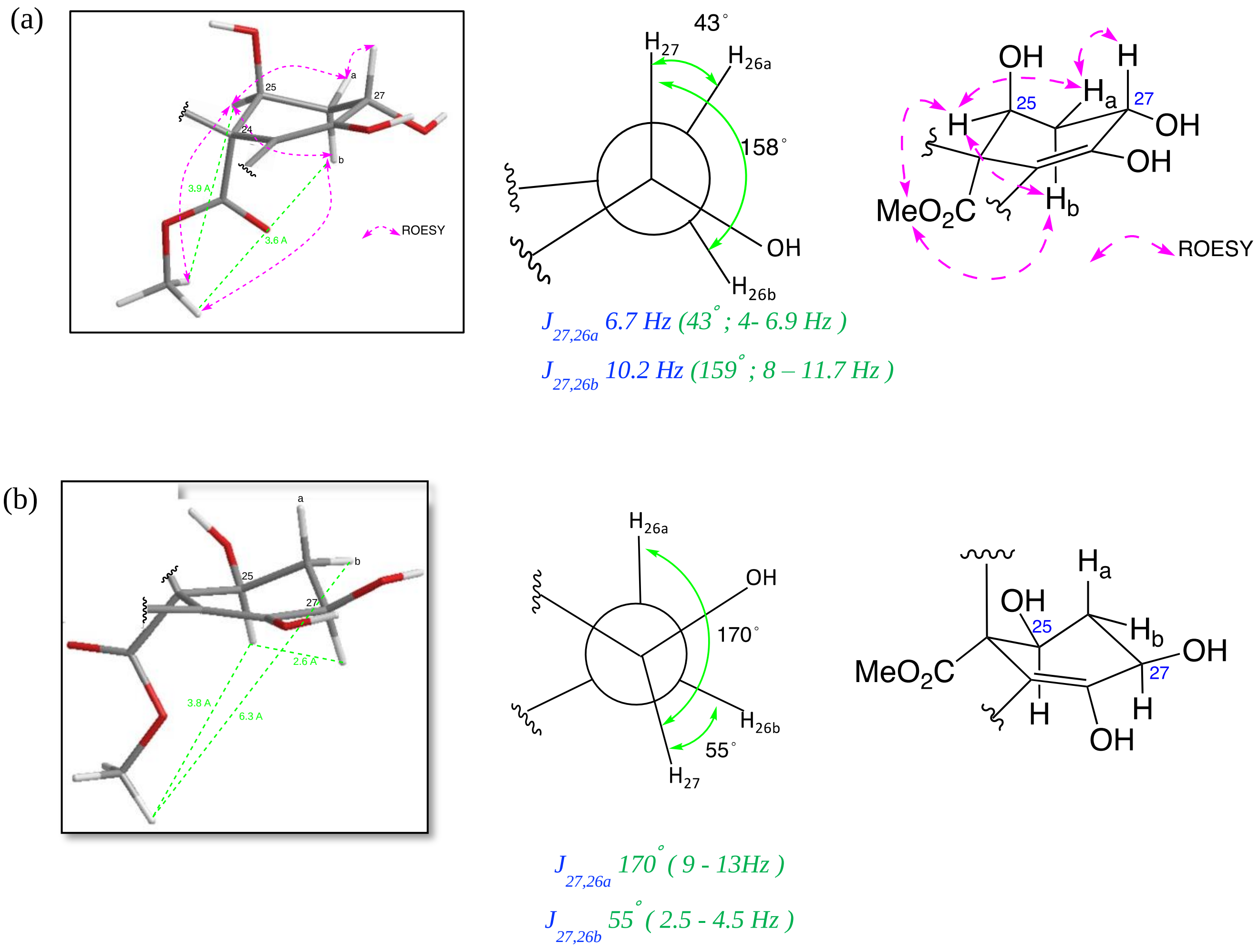

Figure S21. Ring G in minimized models of (a) jugione A (**1**) and (b) its 27*R* epimer .

2.2. Jugione B (2)

Table S6. 1D NMR (600 MHz, $CDCl_3$) data for jugione B (**2**)

Position	δ_C	δ_H, mult, (*J* in Hz)	COSY	HMBC	ROESY
1	161.9	-			
2	119.5	6.97, dd (*8.4, 0.7*)	3	1, 4, 6, 7	1-OH
3	136.2	7.44, dd (*8.4, 7.3*)	2, 4	1, 5	
4	122.2	7.09, dd (*7.3, 0.7*)	3	2, 6, 7, 10	10
5	137.2	-			
6	115.2	-			
7	186.9	-			
8	106.4	-			
9	41.5	-			
10	72.8	6.05, s	-	4, 5, 6, 8, 9, 15, 1′	4, 14, 15a, 15b
11	185.8				
12	43.3	4.48, d (*1.4*)	14	8, 11, 13, 14, 16, 17, 18,	29
13	141.6				
14	125.8	5.68, dq (*1.4, 1.4*)	12, 29	8, 9, 10, 12, 29	10, 15a, 29
15	36.0	*a.* 2.77, d (*17.8*)	15b	8, 9, 10, 14, 16, 21	10, 14, 21
		b. 2.67, d (*17.8*)	15a	9, 14, 16, 20, 21	10, 21
16	149.6	-			
17	115.0	-	-		
18	155.2	-			
19	105.9	-			
20	160.2	-			
21	114.4	6.16, s	-	15, 17, 19, 20	15a, 15b, 20-OH
22	193.9	-			
23	38.2	*a.* 3.18, d (*17.0*)	23b	22, 24, 25, 30	25, 26b
		b. 3.02, d (*17.0*)	23a	19, 22, 30	25, 26b
24	84.2	-			
25	77.7	4.96, dd (*9.8, 6.2*)	26a, 26b	23, 26, 30	23a, 23b, 26a, 27, OMe
26	31.5	*a.* 2.74, ddd (*13.0, 8.9,* [illegible]	25, 26b,	28	25
		b. 2.34, ddd (*13.0, 9.8,*	25, 26a,	24, 25, 27, 28	
27	67.7	4.70, dd (*9.8, 8.9*)	26a, 26b	26, 28	25
28	175.4				
29	21.0	1.88, dd (*1.4, 1.4*)	14	12, 13, 14	12, 14
30	168.8	-			
OMe	54.2	3.76, s		30	25
1′	166.7	-			
2′	119.3	5.74, d (*15.2*)	3′	1′, 4′	4′
3′	146.4	7.23, dd (*15.2, 11.2*)	2′, 4′	1′, 5′	5′
4′	127.5	6.12, dd (*14.8, 11.2*)	3′, 5′	6′	2′
5′	142.2	6.52, dd (*14.8, 10.8*)	4′, 6′	3′, 7′	3′, 7′
6′	131.4	6.13, dd (*14.8, 10.8*)	5′, 7′	4′, 8′	8′
7′	136.0	5.95, dq (*14.8, 6.9*)	6′, 8′	5′, 8′	5′, 8′
8′	18.8	1.82, d (*6.9*)	7′	6′	6′, 7′
1-OH	-	11.65, s		1, 2, 6	2
11-OH	-	14.11, s		8,11, 12	
20-OH	-	11.31, s		19, 20, 21	21

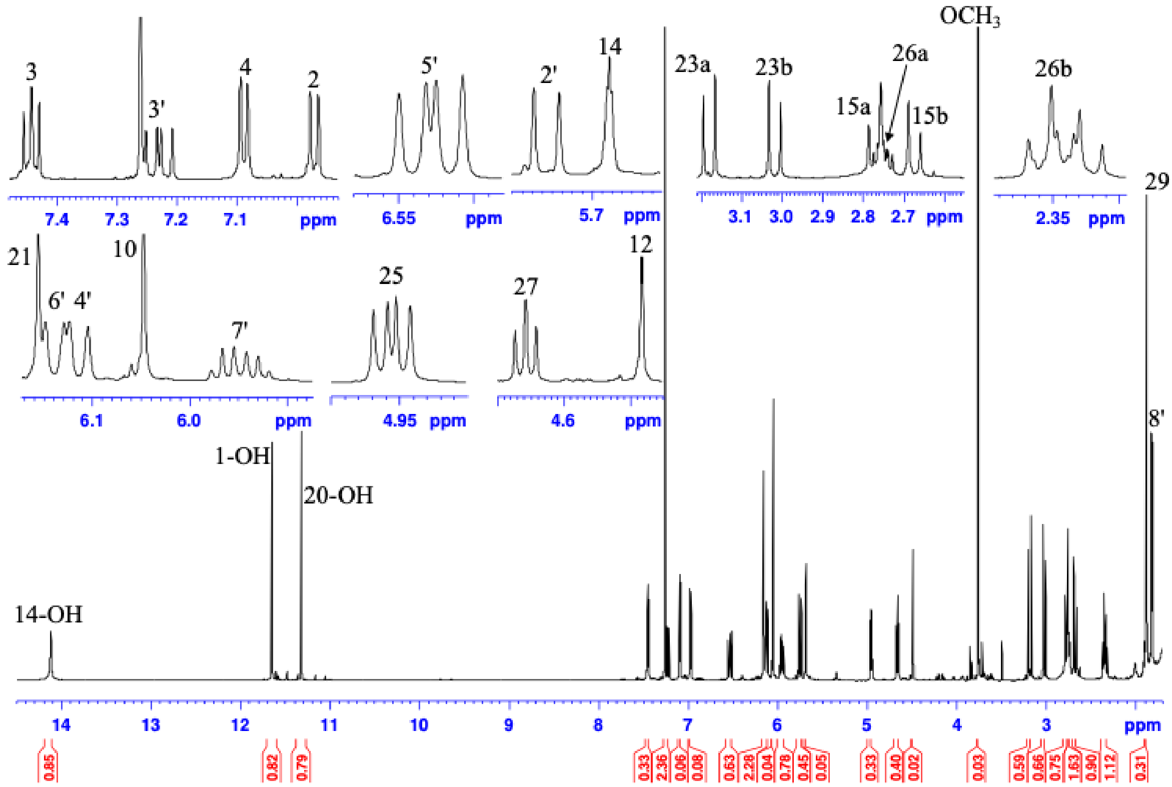

Figure S22. ^{1}H NMR (600 MHz, $CDCl_3$) spectrum of jugione B (**2**)

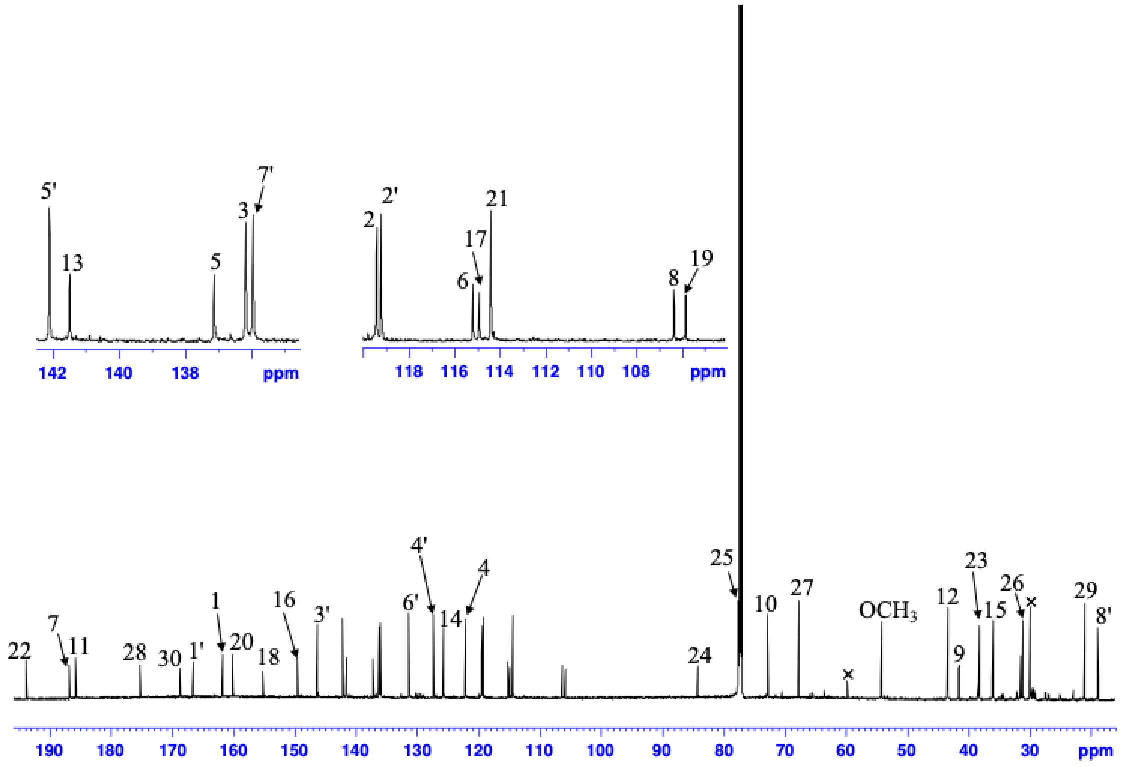

Figure S23. ^{13}C NMR (150 MHz, $CDCl_3$) spectrum of jugione B (**2**)

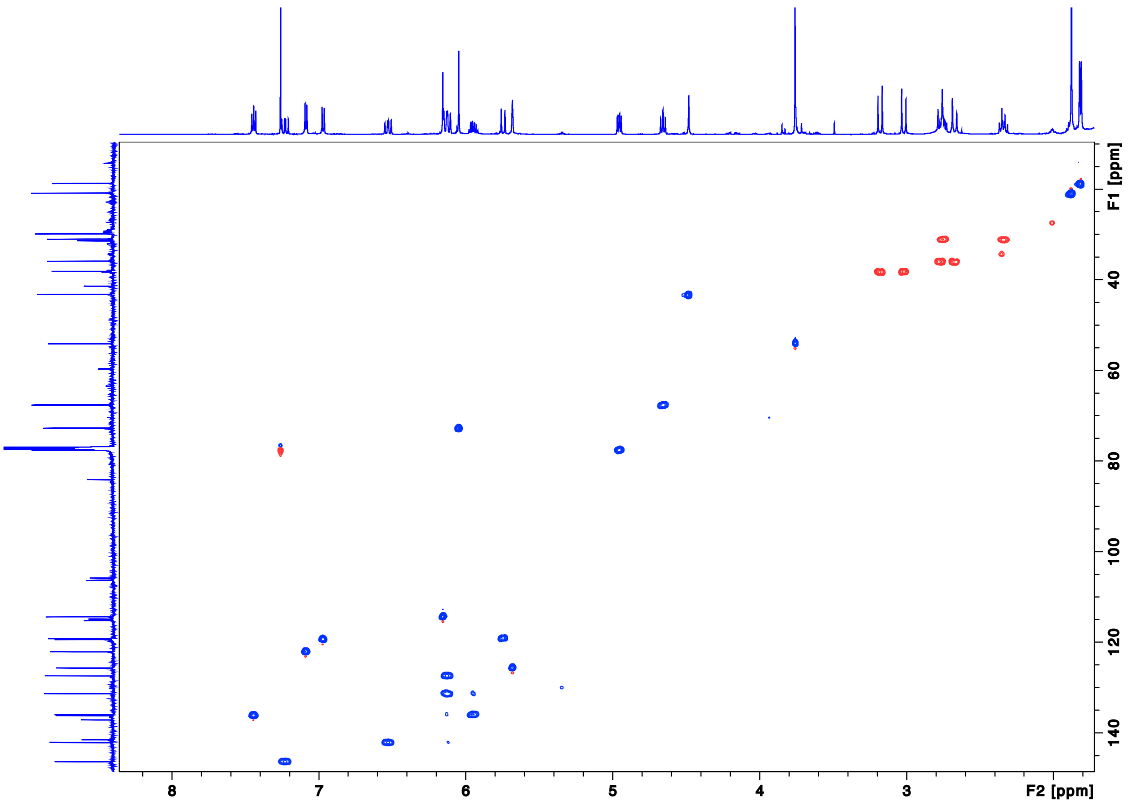

Figure S24. HSQC NMR (600 MHz, $CDCl_3$) spectrum of jugione B (**2**)

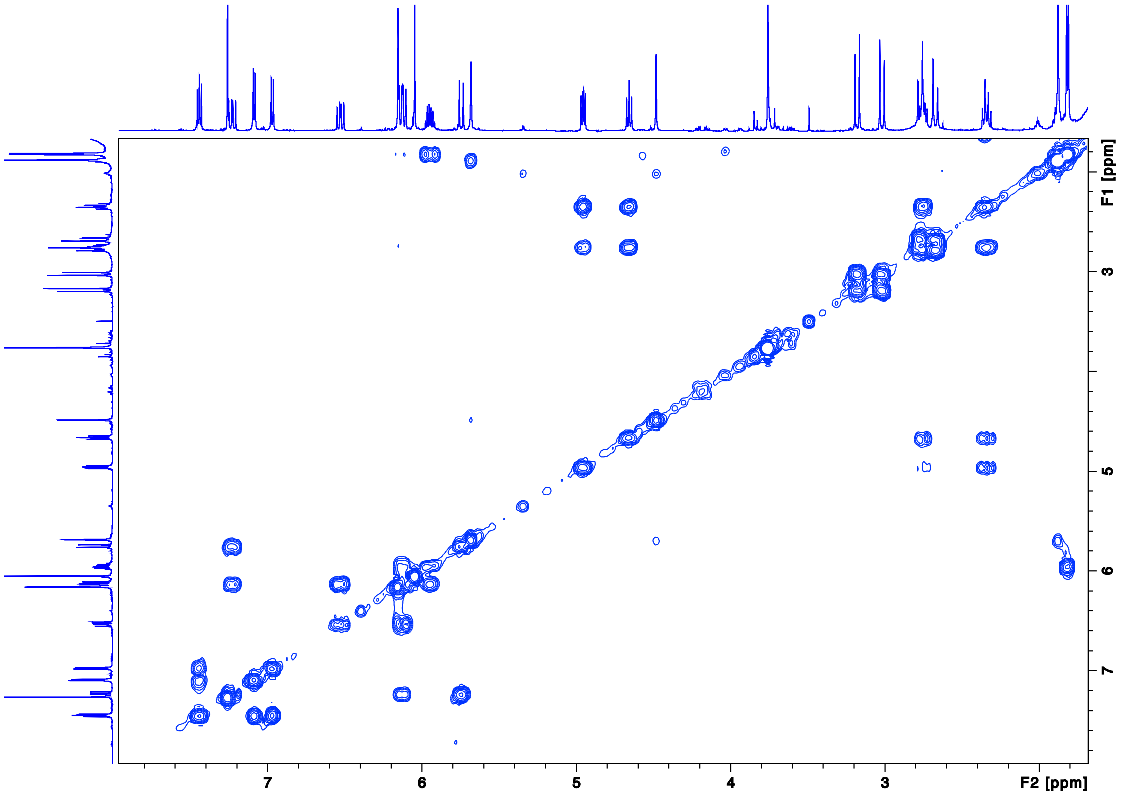

Figure S25. COSY NMR (600 MHz, $CDCl_3$) spectrum of jugione B (**2**)

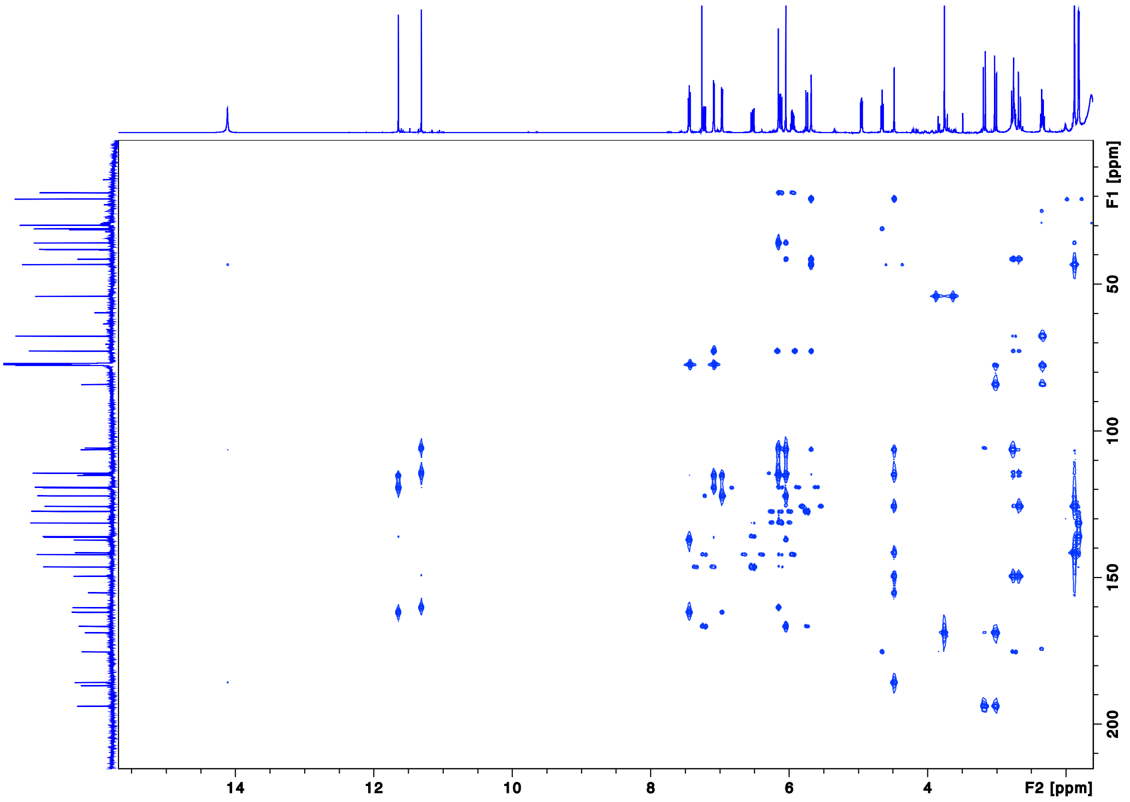

Figure S26. HMBC NMR (600 MHz, $CDCl_3$) spectrum of jugione B (**2**)

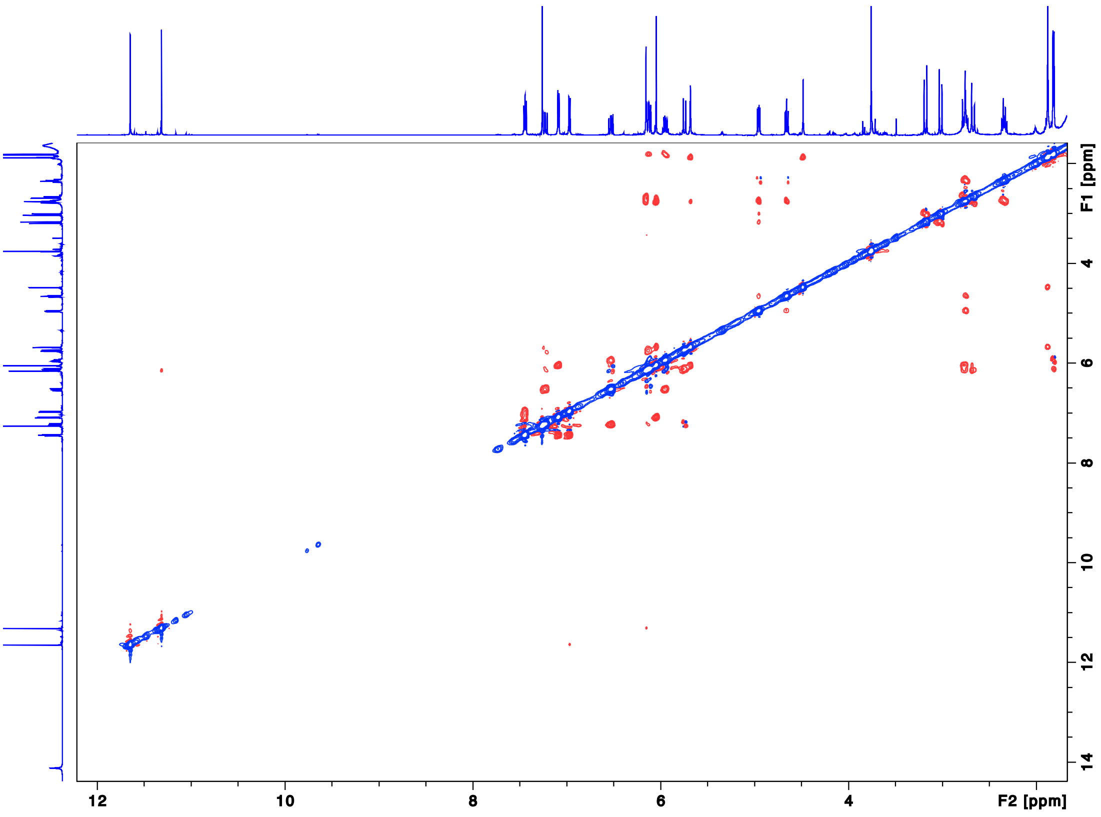

Figure S27. ROESY NMR (600 MHz, $CDCl_3$) spectrum of jugione B (**2**)

Mass Spectrum Molecular Formula Report

Analysis Info

Analysis Name	D:\Data\Thulasi\CMB-STF067_P23_27_5_1.d	Acquisition Date	2/20/2023 4:41:31 PM
Method	tune-medhigh_AP.m	Operator	a.salim
Sample Name	CMB-STF067_P23_27_5_1	Instrument / Ser#	micrOTOF 213750.00232
Comment			

Acquisition Parameter

Source Type	ESI	Ion Polarity	Positive	Set Nebulizer	0.5 Bar
Focus	Not active			Set Dry Heater	180 °C
Scan Begin	100 m/z	Set Capillary	4500 V	Set Dry Gas	5.0 l/min
Scan End	1500 m/z	Set End Plate Offset	-500 V	Set Divert Valve	Source

Generate Molecular Formula Parameter

Formula, min.		
Formula, max.		
Measured m/z	Tolerance	Charge
Check Valence	Minimum	Maximum
Nirogen Rule	Electron Configuration	
Filter H/C Ratio	Minimum	Maximum
Estimate Carbon		

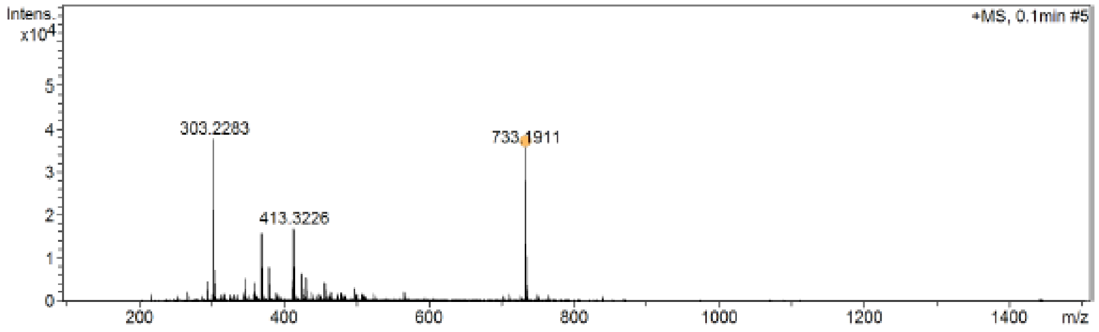

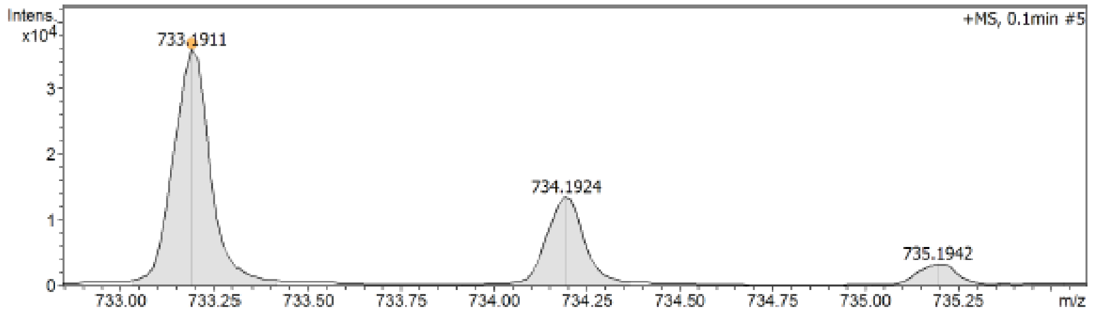

Meas. m/z	#	Ion Formula	m/z	err [ppm]	mSigma	# Sigma	Score	rdb	e¯ Conf	N-Rule
733.1911	1	C30H26N14NaO8	733.1950	5.3	5.8	1	9.32	24.5	even	ok
	2	C32H38NaO18	733.1950	5.3	9.3	2	8.65	13.5	even	ok
	3	C29H30N10NaO12	733.1937	3.5	9.7	3	33.97	19.5	even	ok
	4	C27H18N24NaO2	733.1937	3.5	11.6	4	33.11	30.5	even	ok
	5	C28H34N6NaO16	733.1923	-1.7	20.7	5	73.64	14.5	even	ok
	6	C39H34NaO13	733.1892	2.7	26.8	6	48.80	22.5	even	ok
	7	C36H26N10NaO7	733.1878	4.5	28.7	7	11.20	28.5	even	ok
	8	C27H38N2NaO20	733.1910	0.1	32.2	8	100.00	9.5	even	ok

Bruker Compass DataAnalysis 4.1 printed: 2/20/2023 4:44:46 PM Page 1 of 2

Figure S28. HRMS spectrum and measurement for jugione B (**2**)

Table S7. NMR (600 MHz, methanol-d_4) data for jugione B

Position	δ_C	δ_H, mult, (*J* in Hz)	COSY	HMBC
1	163.1	-		
2	120.2	6.97, d (*8.2*)	3	
3	137.3	7.51, dd (*8.2, 7.1*)	2, 4	
4	123.3	7.11, d (*7.1*)	3	
5	138.9	-		
6	116.1	-		
7	188.3	-		
8	107.6	-		
9	42.7	-		
10	74.3	6.09, s		
11	186.4	-		
12	44.3	4.57, s	14	
13	142.9	-		
14	126.8	5.70, s	12, 29	9, 12, 29
15	36.4	*a.* 2.86, d (*18.0*)	15b	9, 14, 16, 17
		b. 2.63, d (*18.0*)	15a	9, 14, 16, 17
16	150.4	-		
17	116.2	-		
18	156.9	-		
19	106.8	-		
20	161.1	-		
21	114.6	6.18, s		
22	196.1	-		
23	39.6	*a.* 3.22, d (*17.1*)	23b	22, 24, 25, 30
		b. 3.12, d (*17.1*)	23a	22
24	85.4	-		
25	78.8	5.00, dd (*10.1, 6.2*)	26a, 26b	
26	32.4	*a.* 2.79, ddd (*14.0, 8.7, 6.2*)	25, 26b, 27	25
		b. 2.31, ddd (*14.0, 10.1, 9.6*)	25, 26a, 27	24, 25, 27
27	68.4	4.69, dd (*9.6, 8.7*)	26a, 26b	26, 28
28	177.6	-		
29	20.8	1.90, s	14	12, 13, 14
30	170.6	-		
30-OMe	54.0	3.72, s		30
1′	167.8	-		
2′	119.9	5.76, d (*15.2*)	3′	1′, 4′
3′	147.6	7.23, dd (*15.5, 11.5*)	2′, 4′	1′, 5′
4′	128.6	6.22, dd (*14.7, 11.5*)	3′, 5′	6′
5′	143.4	6.59, dd (*14.7, 11.1*)	4′, 6′	3′, 7′
6′	132.5	6.14, dd (*15.0, 11.1*)	5′, 7′	4′, 8′
7′	136.7	5.99, dq (*15.0, 6.8*)	6′, 8′	5′, 8′
8′	18.6	1.80, d (*6.8*)	7′	6′

[A] overlapping signals

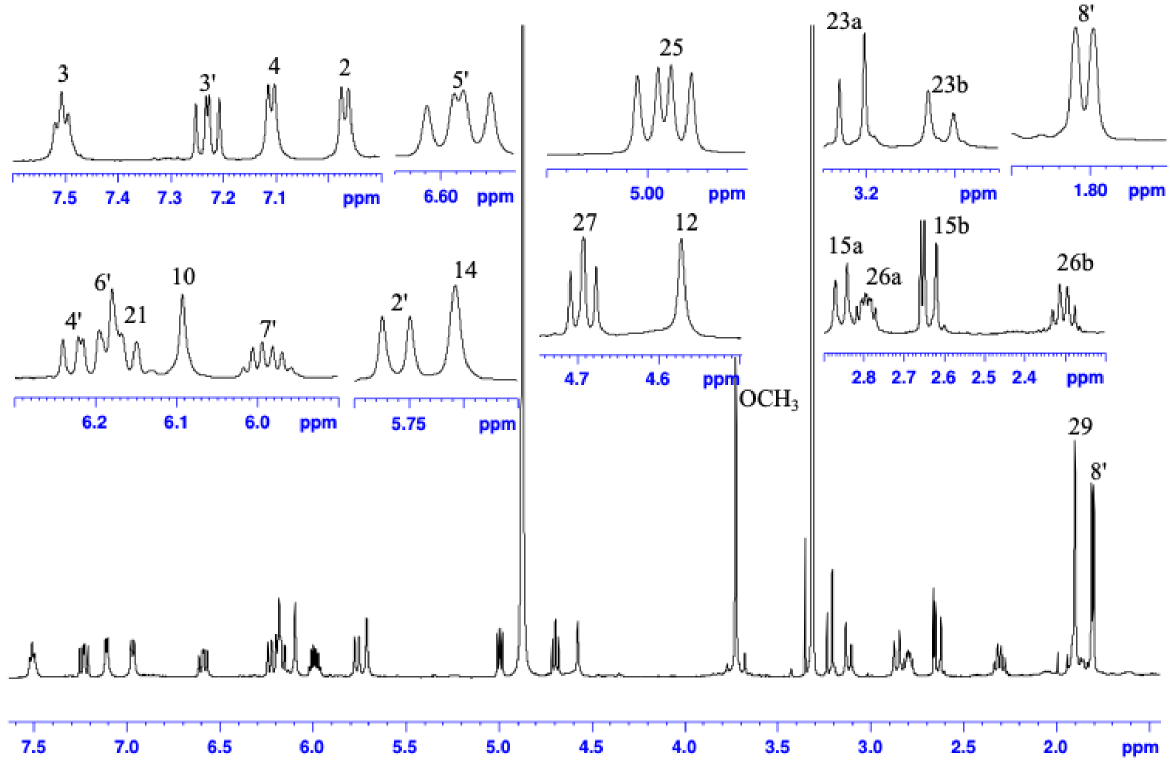

Figure S29. ^{1}H NMR (600 MHz, methanol-d_4) spectrum of jugione B (**2**)

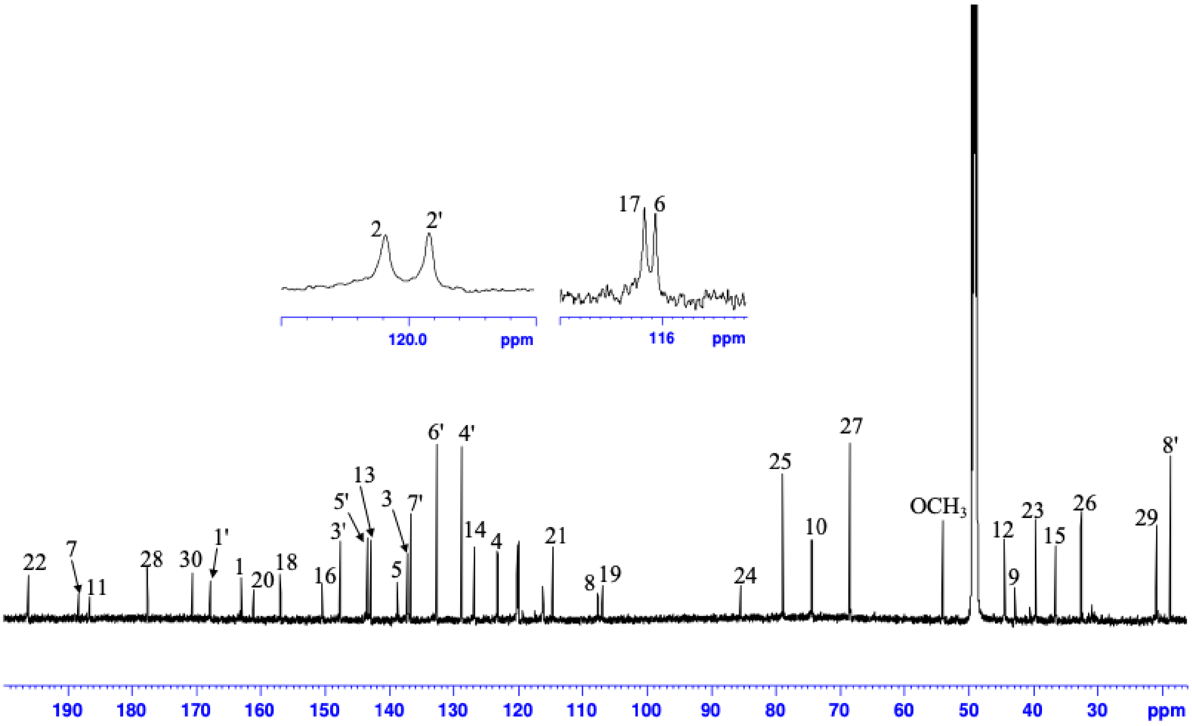

Figure S30. ^{13}C NMR (150 MHz, methanol-d_4) spectrum of jugione B (**2**)

Table S8. Comparison of 1D NMR (methanol-d_4) data for engyodontochones D (**7**) with jugione B (**2**)

Pos.	engyodontochone D (**7**)[12]		jugione B (**2**)	
	δ_C	δ_H, mult, (*J* in Hz)	δ_C	δ_H, mult, (*J* in Hz)
1	162.7	-	162.9	-
2	120.1	6.98, d (*8.3*)	120.1	6.97, d (*8.2*)
3	137.1	7.52, t (*8.3*)	137.2	7.51, dd (*8.2*, *7.1*)
4	123.2	7.11, d (*8.3*)	123.1	7.11, d (*7.1*)
5	138.5	-	138.7	-
6	115.0	-	116.1	-
7	187.3	-	188.3	-
8	107.2	-	106.8	-
9	41.7	-	42.7	-
10	74.6	NI	74.3	6.09, s
11	187.3	-	186.4	-
12	44.9	4.37, d (*1.5*)	44.4	4.57, s
13	143.0	-	142.9	-
14	126.5	5.78, t (*1.5*)	126.8	5.70, s
15	36.3	*a.* 2.89, d (*17.8*)<br>*b.* 2.58, d (*17.8*)	36.4	*a.* 2.86, d (*18.0*)<br>*b.* 2.63, d (*18.0*
16	150.0	-	150.4	
17	115.8	-	116.2	-
18	157.1	-	156.9	-
19	106.9	-	107.7	-
20	161.1	-	161.1	-
21	114.3	6.15, s	114.6	6.18, s
22	195.8	-	196.1	-
23	40.2	*a.* 3.68, overlap,<br>*b.* 3.07, d (*17.3*)	39.6	*a.* 3.22, d (*17.1*)<br>*b.* 3.12, d (*17.1*)
24	87.0	-	85.4	-
25	83.0	5.11, dd (*8.6, 3.3*)	78.8	5.00, dd (*10.1*, *6.2*)
26	23.8	2.66 (2H), m	32.4	*a.* 2.79, ddd (*14.0*, *8.7*, *6.2*)<br>*b.* 2.31, ddd (*14.0*, *10.1*, *9.6*)
27	28.6	2.74 (2H), m	68.4	4.69, dd (*9.6*, *8.7*)
28	179.1	-	177.5	-
29	20.6	1.99, s	20.8	1.90, s
30	170.4	-	170.6	-
OMe	53.9	3.78, s	54.0	3.72, s
1′	171.8	-	167.8	-
2′	20.9	1.99, s	119.9	5.76, d (*15.2*)
3′	-	-	147.7	7.23, dd (*15.5*, *11.5*)
4′	-	-	128.4	6.22, dd (*14.7*, *11.5*)
5′	-	-	143.4	6.59, dd (*14.7*, *11.1*)
6′	-	-	132.6	6.14, dd (*15.0*, *11.1*)
7′	-	-	136.7	5.99, dq (*15.0*, *6.8*)
8′	-	-	18.6	1.80, d (*6.8*)
1-OH	-	-	-	-
11-OH	-	-	-	-
20-OH	-	-	-	-

NI - not indicated

2.3. Jugione C (3)

Table S9. NMR (600 MHz, methanol-d_4) data for jugione C (**3**)

Position	δ_C	δ_H, mult, (*J* in Hz)	COSY	HMBC	ROESY
1	162.8	-			
2	120.0	6.94, d (*8.4*)	3	4, 6	3
3	137.0	7.48, dd (*8.4, 7.3*)	2, 4	1, 5	2, 4
4	123.0	7.09, d (*7.3*)	3	2, 6	3, 10
5	138.7	-			
6	116.2	-			
7	ND	-			
8	107.5[A]	-			
9	42.8	-			
10	74.5	6.07, br s	-	1′, 4, 5, 6, 8, 9, 15	4, 14, 15a
11	187.4[A]	-			
12	44.4	4.74, s		8, 11, 13, 14, 16, 17, 18, 29	29
13	143.2	-			
14	126.6	5.68, dq (*1.4, 1.4*)	29	8, 9, 10, 12, 29	29
15	36.6	*a.* 2.83, d (*17.8*)	15b	8, 9, 16, 17, 21	10, 21
		b. 2.60, d (*17.8*)	15a	9, 14, 16, 17, 21	21
16	150.0	-			
17	116.2[A]	-	-		
18	157.6	-			
19	106.9	-			
20	161.0	-			
21	114.1	6.11, s	-	15, 17, 19, 20	15a, 15b
22	198.1	-			
23	40.6	*a.* 3.22, d (*17.3*)	23b	22, 24, 25, 30	
		b. 3.10, d (*17.3*)	23a	22	25
24	89.2	-			
25	72.8	4.31, br d (*10.9*)	26a	26, 27	23b, 26b
26	37.7	*a.* 2.04, ddd (*14.3, 10.9, 3.0*)	25, 26b, 27		27
		b. 1.85, dd (*14.3, 9.8*)	25, 26a, 27		25
27	69.5	4.25, dd (*9.8, 3.0*)	26a, 26b	25, 26, 28	26a
28	180.3	-			
29	20.7	1.91, br s	14	12, 13, 14	12, 14
30	171.9	-			
OMe	53.4	3.67, s		30	
1′	167.8	-			
2′	120.0	5.76, d (*15.2*)	3′	1′, 4′	4′
3′	147.6	7.22, dd (*15.2, 11.3*)	2′, 4′	1′, 5′	5′
4′	128.6	6.21, dd (*14.9, 11.3*)	3′, 5′	6′	2′
5′	143.4	6.58, dd (*14.9, 10.7*)	4′, 6′	3′, 7′	3′, 7′
6′	132.5	6.16, dd (*14.9, 10.7*)	5′, 7′	4′, 8′	8′
7′	136.6	5.98, dq (*14.9, 6.8*)	6′, 8′	5′, 8′	5′
8′	18.6	1.79, d (*6.8*)	6′, 7′	6′, 7′	6′

[A] detected by HMBC

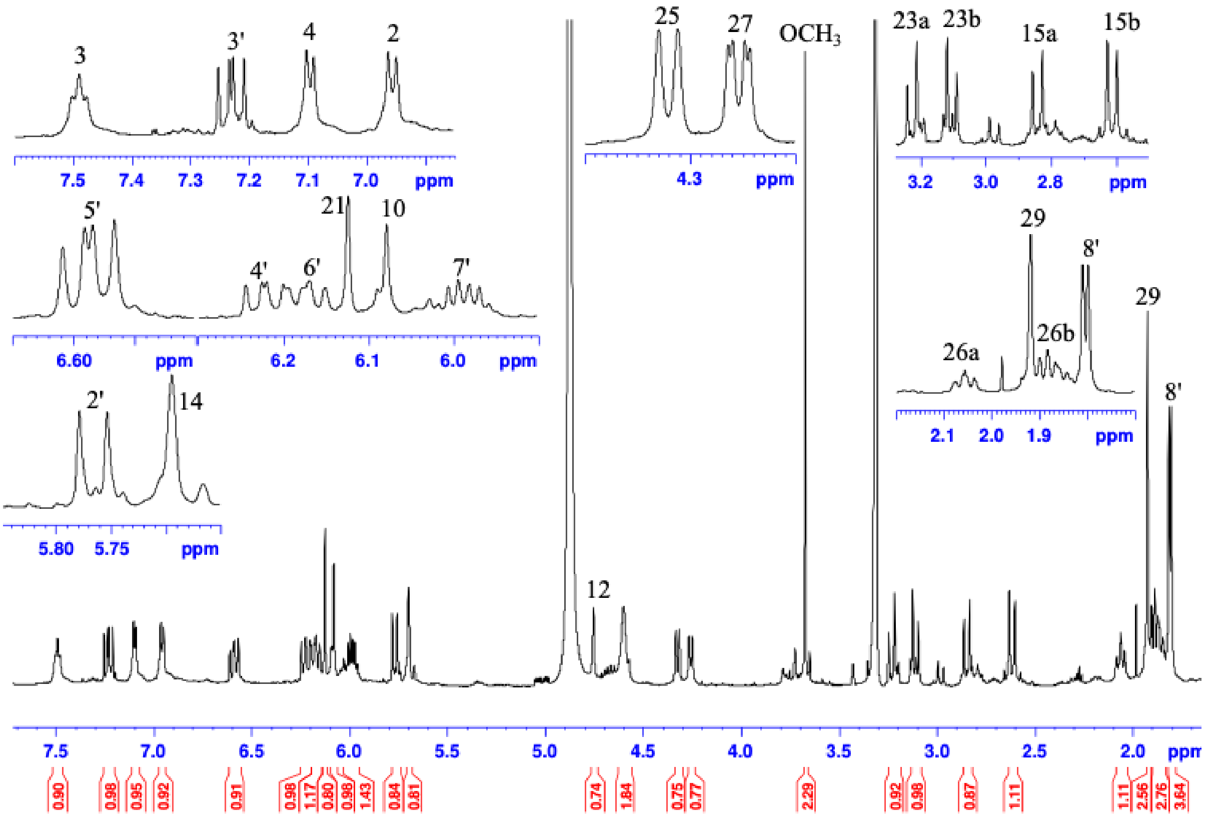

Figure S31. ^{1}H NMR (600 MHz, methanol-d_4) spectrum of jugione C (**3**)

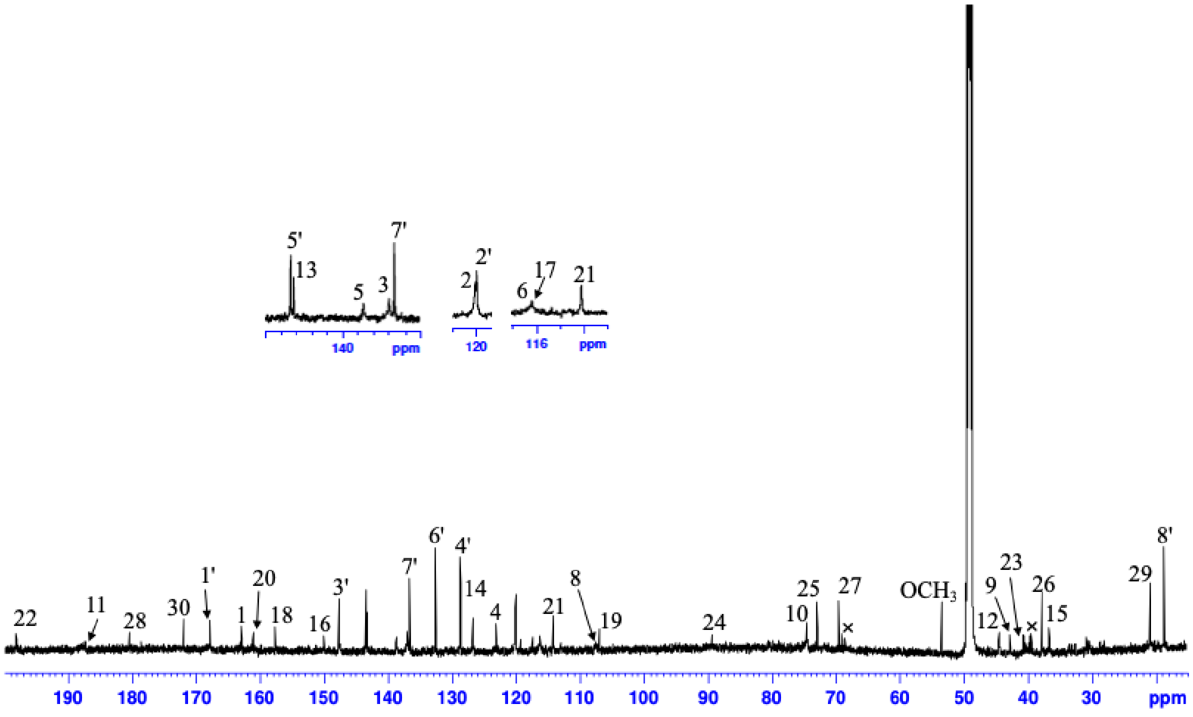

Figure S32. ^{13}C NMR (150 MHz, methanol-d_4) spectrum of jugione C (**3**)

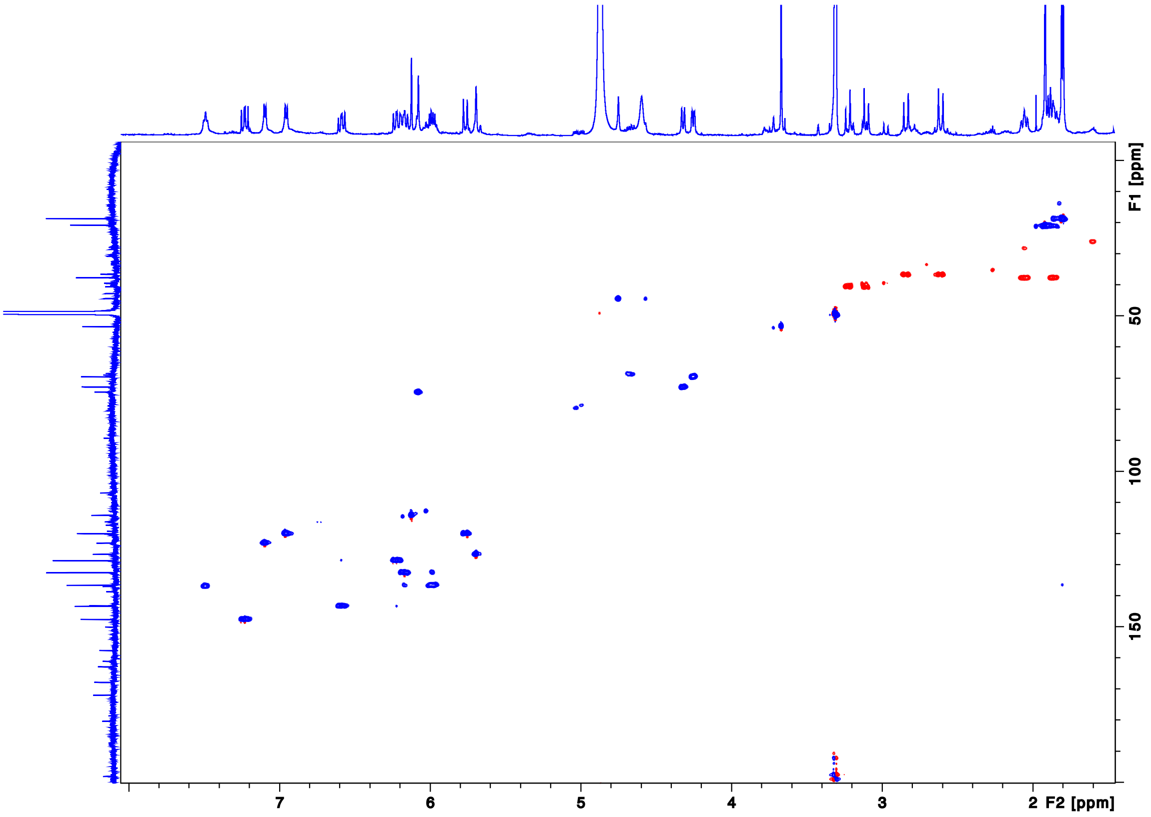

Figure S33. HSQC NMR (600 MHz, methanol-d_4) spectrum of jugione C (**3**)

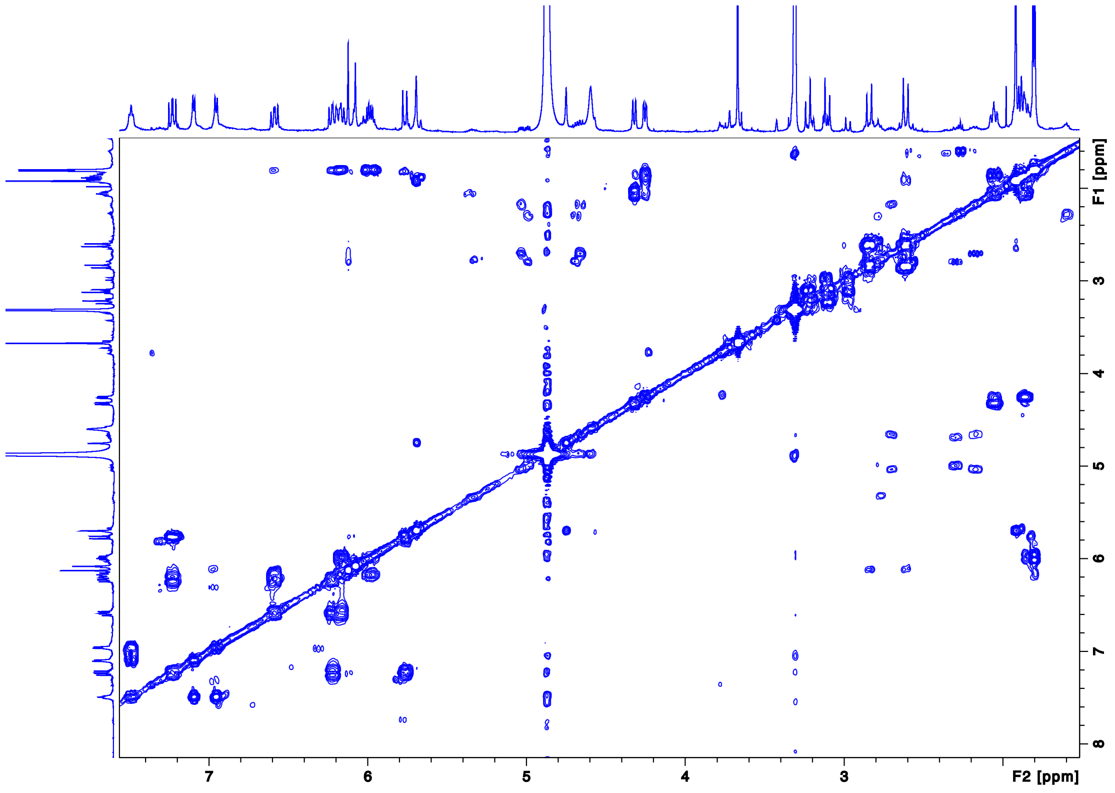

Figure S34. COSY NMR (600 MHz, methanol-d_4) spectrum of jugione C (**3**)

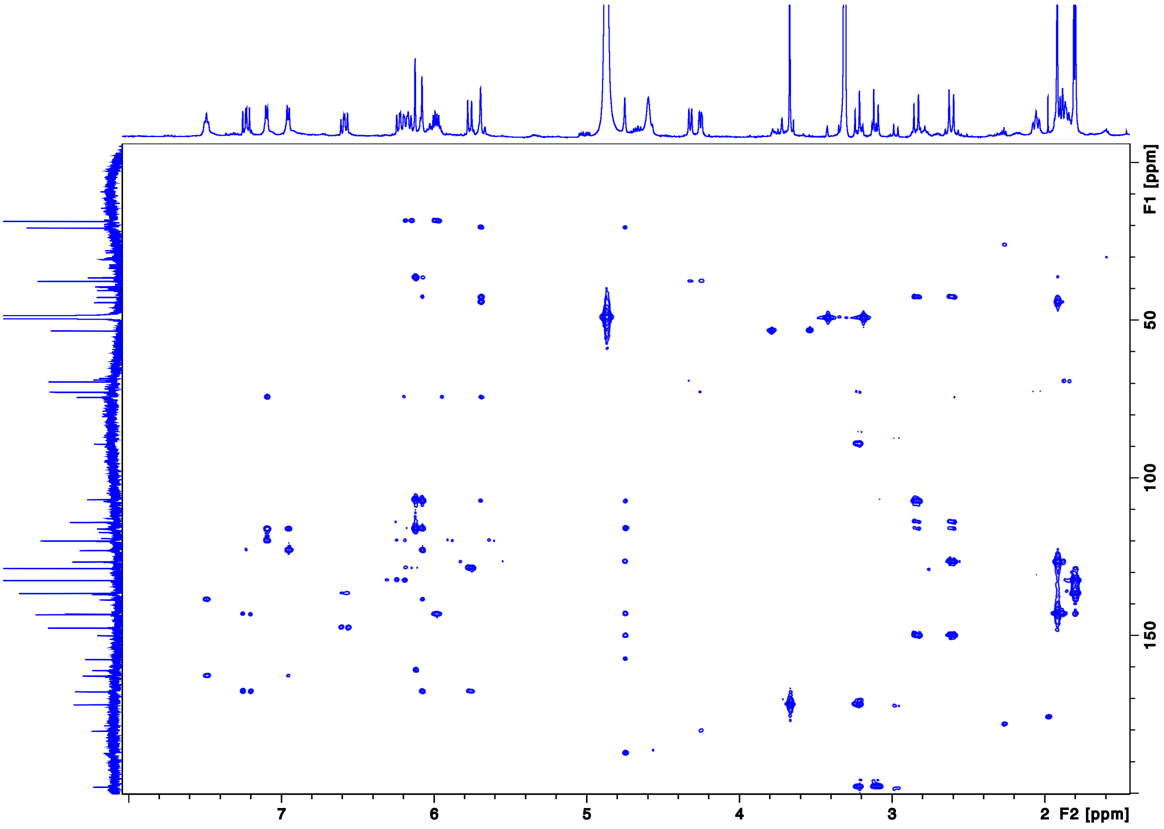

Figure S35. HMBC NMR (600 MHz, methanol-d_4) spectrum of jugione C (**3**)

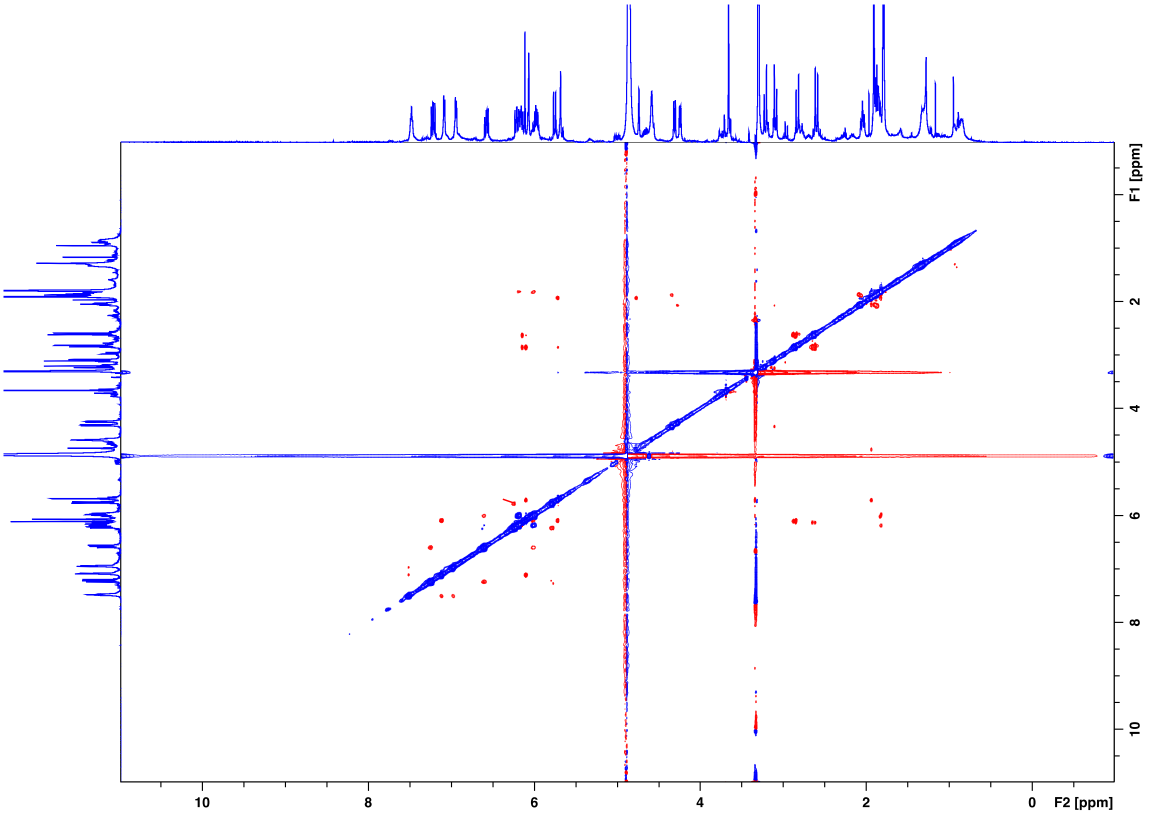

Figure S36. ROESY NMR (600 MHz, methanol-d_4) spectrum of jugione C (**3**)

Mass Spectrum Molecular Formula Report

Analysis Info

Analysis Name	D:\Data\Thulasi\CMB-STF067_P23_27_2_729.d	Acquisition Date	2/20/2023 4:22:38 PM
Method	tune-medhigh_AP.m	Operator	a.salim
Sample Name	CMB-STF067_P23_27_2_729	Instrument / Ser#	micrOTOF 213750.00232
Comment			

Acquisition Parameter

Source Type	ESI	Ion Polarity	Positive	Set Nebulizer	0.5 Bar
Focus	Not active			Set Dry Heater	180 °C
Scan Begin	100 m/z	Set Capillary	4500 V	Set Dry Gas	5.0 l/min
Scan End	1500 m/z	Set End Plate Offset	-500 V	Set Divert Valve	Source

Generate Molecular Formula Parameter

Formula, min.		
Formula, max.		
Measured m/z	Tolerance	Charge
Check Valence	Minimum	Maximum
Nirogen Rule	Electron Configuration	
Filter H/C Ratio	Minimum	Maximum
Estimate Carbon		

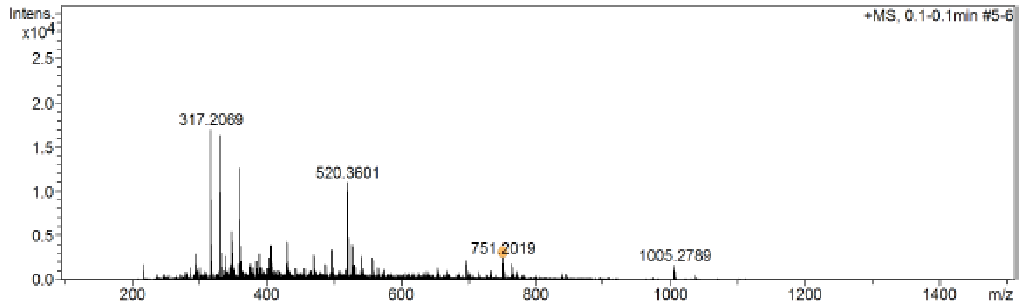

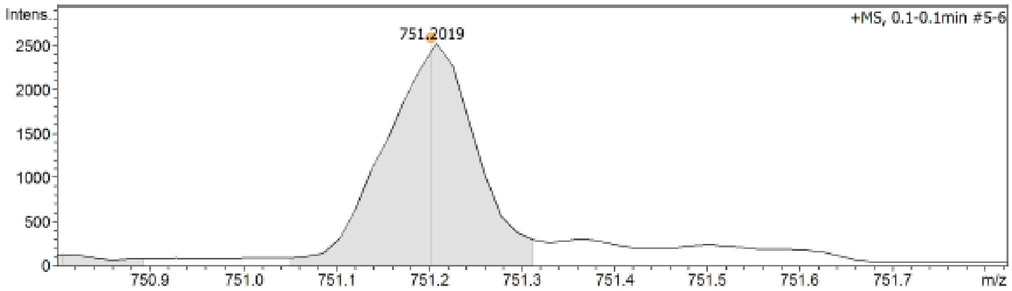

Meas. m/z	#	Ion Formula	m/z	err [ppm]	mSigma	# Sigma	Score	rdb	e¯ Conf	N-Rule
751.2019	1	C39H36NaO14	751.1997	2.8	30.0	1	48.56	21.5	even	ok
	2	C32H40NaO19	751.2056	5.0	35.6	2	8.96	12.5	even	ok
	3	C40H32N4NaO10	751.2011	1.1	36.2	3	100.00	26.5	even	ok
	4	C36H28N10NaO8	751.1984	4.6	37.5	4	11.48	27.5	even	ok
	5	C30H28N14NaO9	751.2056	5.0	40.8	5	7.88	23.5	even	ok
	6	C37H24N14NaO4	751.1997	2.8	43.8	6	33.43	32.5	even	ok
	7	C29H32N10NaO13	751.2043	3.2	44.3	7	26.67	18.5	even	ok
	8	C41H28N8NaO6	751.2024	0.7	49.1	8	78.61	31.5	even	ok

Bruker Compass DataAnalysis 4.1 printed: 2/20/2023 4:29:23 PM Page 1 of 2

Figure S37. HRMS spectrum and measurement for jugione C (**3**)

2.4. Jugione D (4)

Table S10. NMR (600 MHz, methanol-d_4) data for jugione D (**4**)

Position	δ_C	δ_H, mult, (*J* in Hz)	COSY	HMBC	ROESY
1	163.1	-			
2	120.1	6.95, d (*8.3*)	3	4, 6	3
3	137.1	7.49, dd (*8.3*, *7.2*)	2, 4	1, 5	2, 4
4	123.1	7.09, d (*7.2*)	3	2, 6, 10	3, 10
5	138.7	-			
6	116.2	-			
7	ND	-			
8	107.5[A]	-			
9	42.7	-			
10	74.4	6.08, s	-	4, 6, 8, 1′	4, 14, 15a, 15b
11	187.3[A]	-			
12	44.4	4.71, s	14	8, 11, 13, 14, 16, 17, 18, 29	29
13	143.0	-			
14	126.8	5.70, s	12, 29	8, 9, 10, 12, 29	10
15	36.5	*a.* 2.84, d (*17.8*)	15b	8, 9, 16, 17, 21	10, 21
		b. 2.61, d (*17.8*)	15a	9, 14, 16, 17, 21	10, 21
16	150.1	-			
17	116.1	-	-		
18	157.4	-			
19	106.9				
20	161.1	-			
21	114.2	6.12, s	-	15, 17, 19, 20	15a, 15b
22	197.9	-			
23	40.3	*a.* 3.21, d (*17.3*)	23b	22, 24, 25, 30	25, 26b
		b. 3.08, d (*17.3*)	23a	22	25, 26a
24	89.2	-			
25	71.7	4.33, d (*10.8*)	26a, 26b	24, 26	23a, 23b, 27
26	36.9	*a.* 1.99, dd (*13.2*, *10.8*)	25, 26b, 27		23b, 27
		b. 1.87, dd (*13.2*, *10.8*)	25, 26a, 27		23a
27	68.3	4.45, dd (*10.8*, *2.4*)	26a, 26b	25, 26, 28	25, 26a
28	176.6				
29	20.7	1.91, br s	14	12, 13, 14	12, 14
30	171.8	-			
28-OMe	52.6	3.75, s		28	
30-OMe	53.5	3.66, s		30	
1′	167.8	-			
2′	120.0	5.75, d (*15.2*)	3′	1′, 4′	4′, 6′
3′	147.6	7.22, dd (*15.2*, *11.3*)	2′, 4′	1′, 5′	5′
4′	128.6	6.21, dd (*14.8*, *11.3*)	3′, 5′	2′, 6′	2′
5′	143.4	6.58, dd (*14.8*, *11.0*)	4′, 6′	3′, 7′	3′, 7′
6′	132.5	6.16, dd (*15.0*, *11.0*)	5′, 7′	4′, 8′	8′
c	136.7	5.98, dq (*15.0*, *6.8*)	6′, 8′	5′, 8′	5′
8′	18.6	1.79, d (*6.8*)	6′, 7′	6′	6′

[A] Detected by HMBC

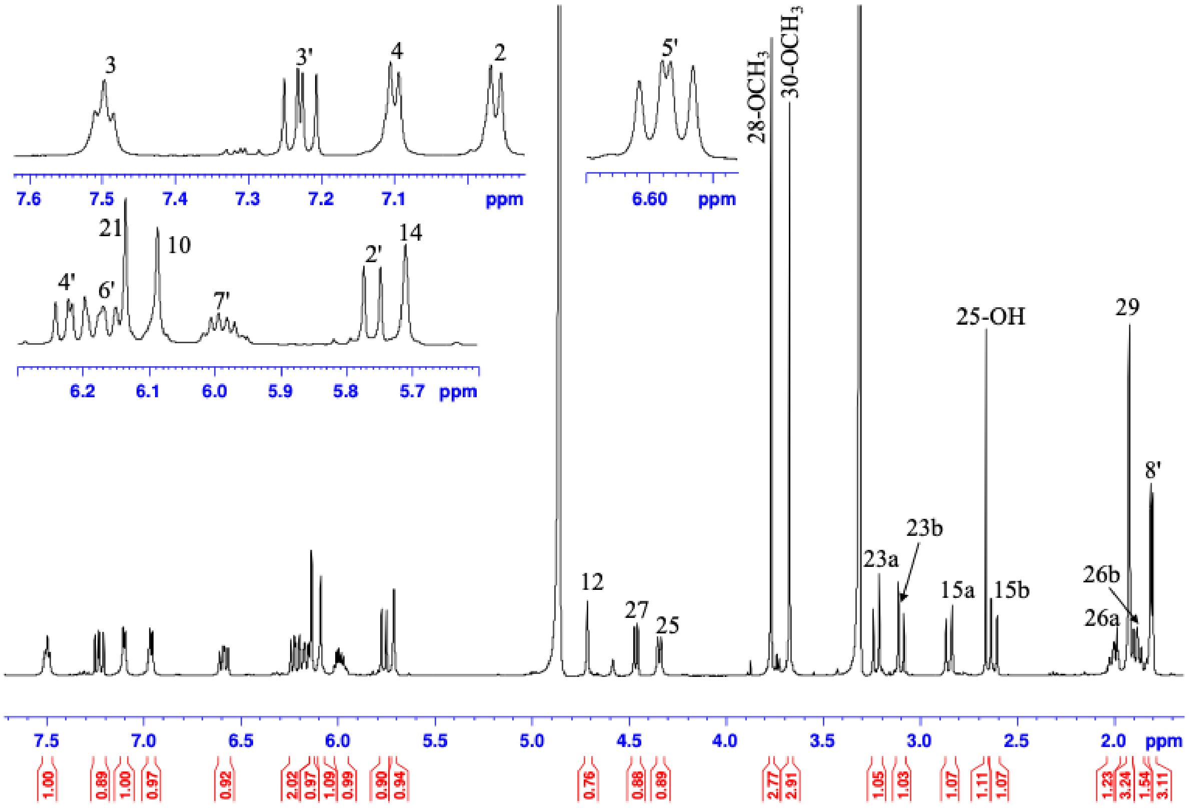

Figure S38. ^{1}H NMR (600 MHz, methanol-d_4) spectrum of jugione D (**4**)

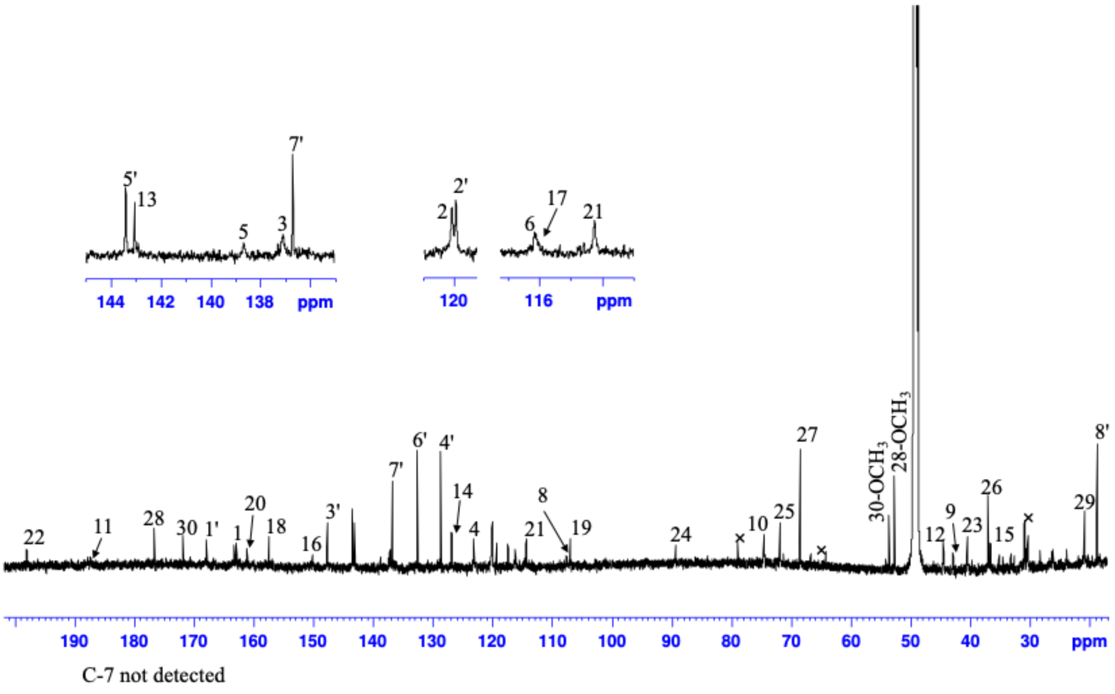

Figure S39. ^{13}C NMR (150 MHz, methanol-d_4) spectrum of jugione D (**4**)

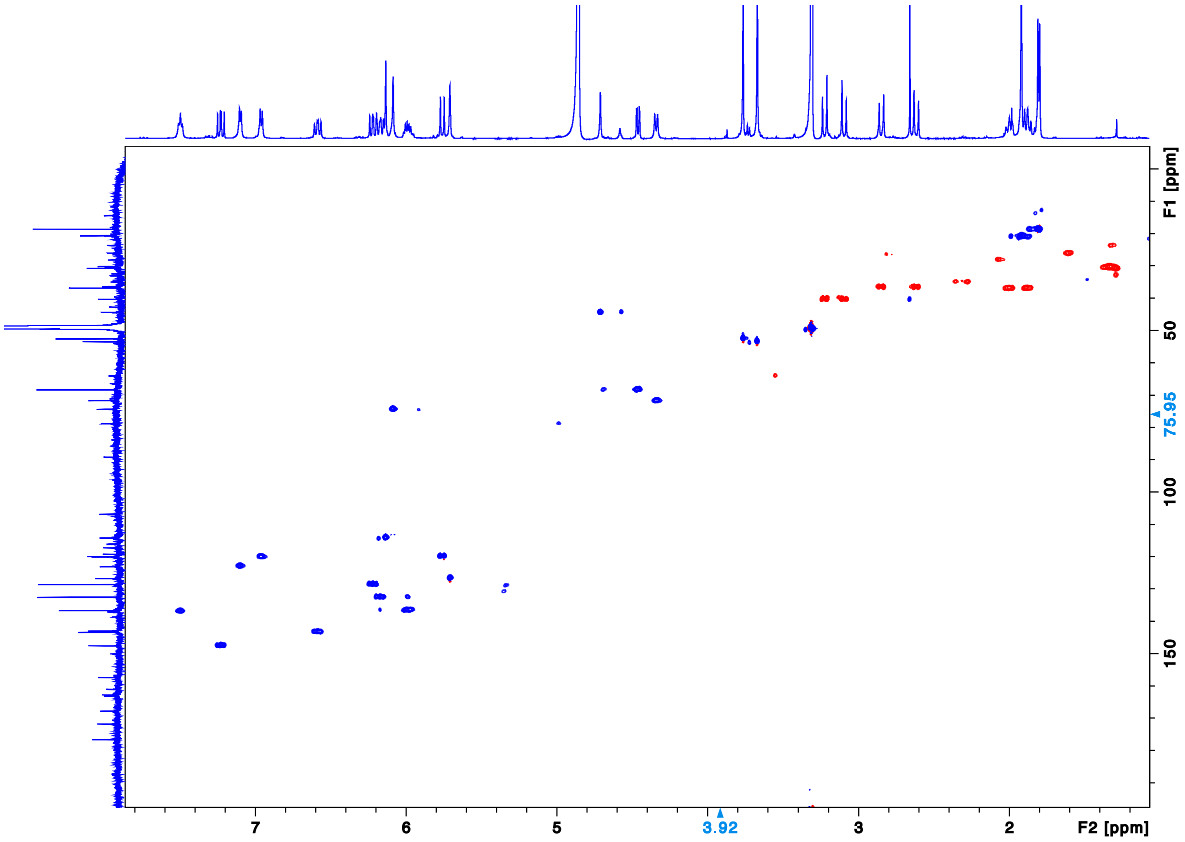

Figure S40. HSQC NMR (600 MHz, methanol-d_4) spectrum of jugione D (**4**)

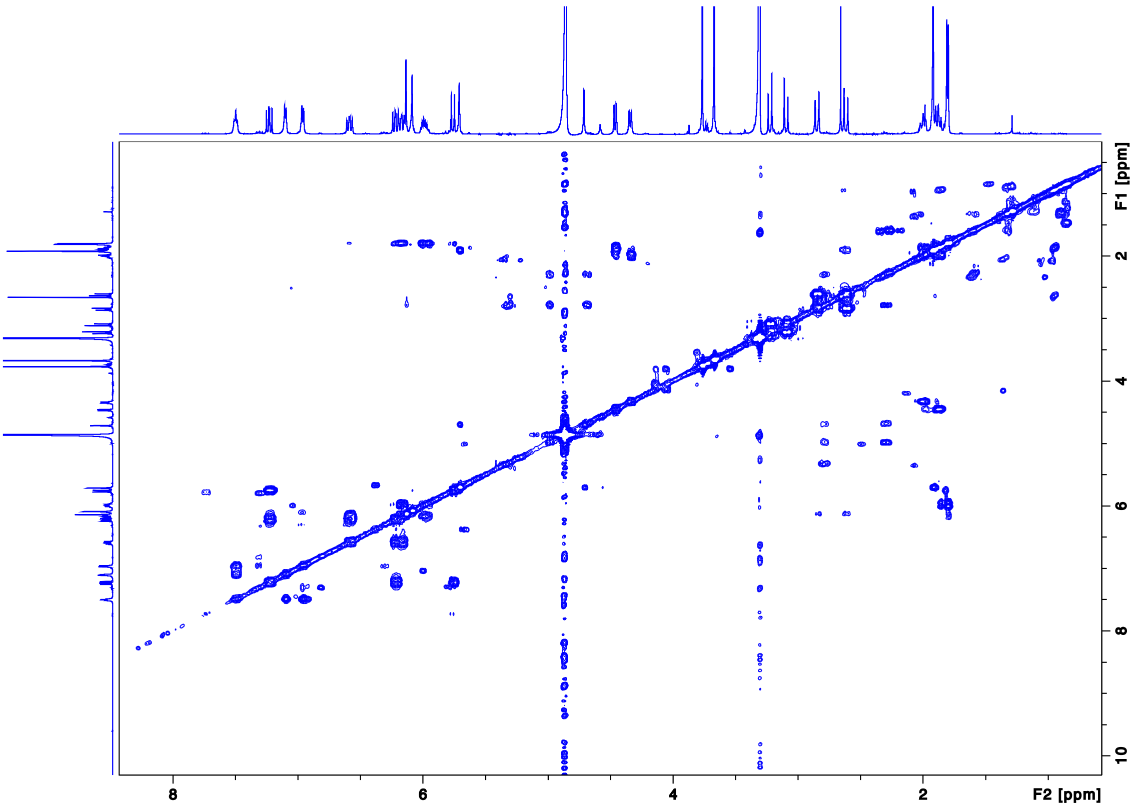

Figure S41. COSY NMR (600 MHz, methanol-d_4) spectrum of jugione D (**4**)

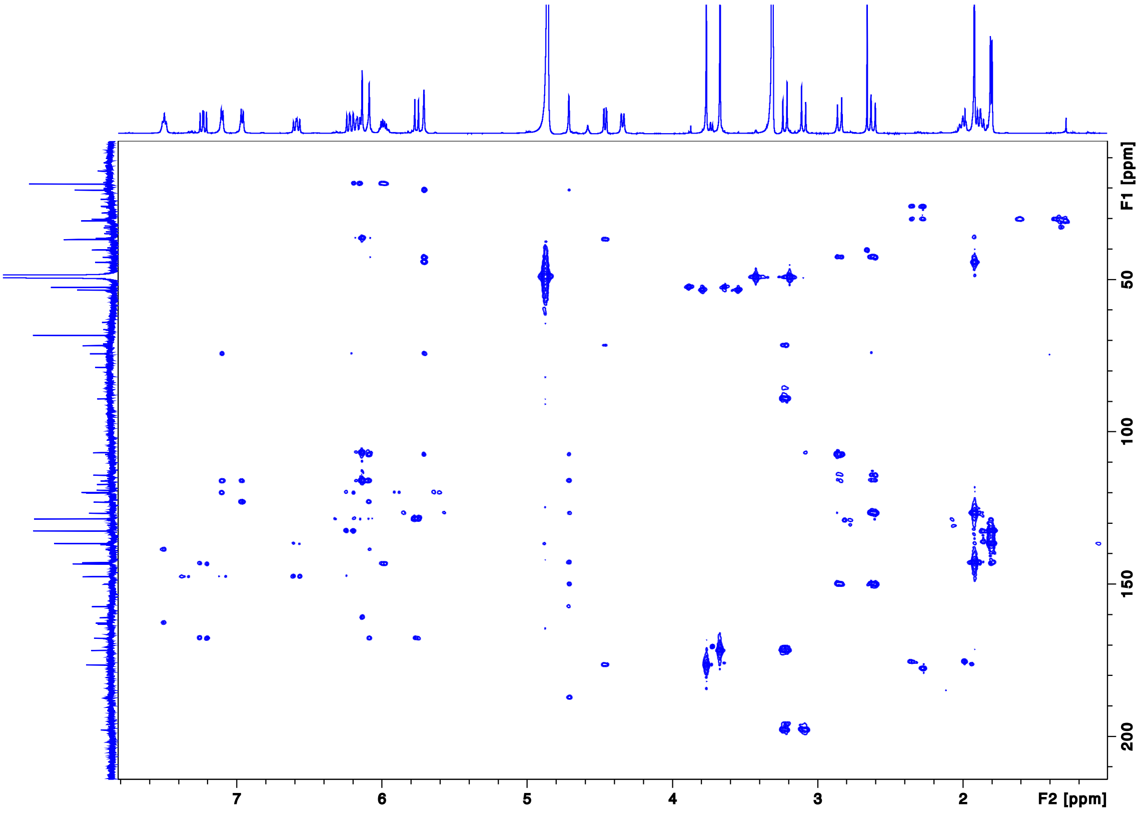

Figure S42. HMBC NMR (600 MHz, methanol-d_4) spectrum of jugione D (**4**)

Mass Spectrum Molecular Formula Report

Analysis Info

Analysis Name	D:\Data\Thulasi\STF067_4_743.d	Acquisition Date	6/15/2023 9:29:13 AM
Method	tune-medhigh_AP.m	Operator	a.salim
Sample Name	STF067_4_743	Instrument / Ser#	micrOTOF 213750.00232
Comment	sample in H2O		

Acquisition Parameter

Source Type	ESI	Ion Polarity	Positive	Set Nebulizer	0.5 Bar
Focus	Not active			Set Dry Heater	180 °C
Scan Begin	100 m/z	Set Capillary	4500 V	Set Dry Gas	5.0 l/min
Scan End	1500 m/z	Set End Plate Offset	-500 V	Set Divert Valve	Source

Generate Molecular Formula Parameter

Formula, min.
Formula, max.
Measured m/z — Tolerance — Charge
Check Valence — Minimum — Maximum
Nirogen Rule — Electron Configuration
Filter H/C Ratio — Minimum — Maximum
Estimate Carbon

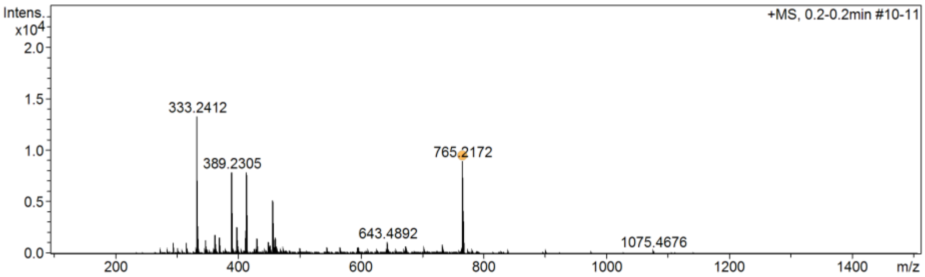

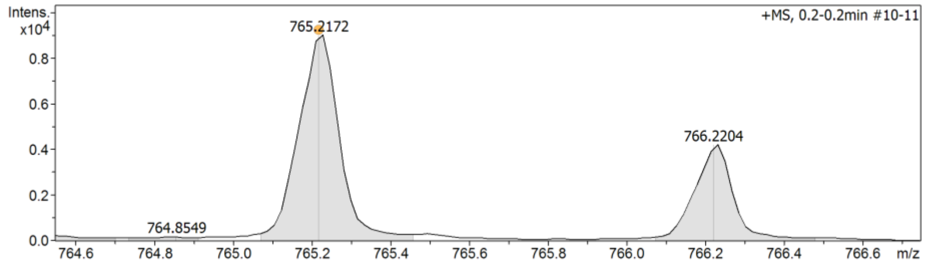

Meas. m/z	#	Ion Formula	m/z	err [ppm]	mSigma	# Sigma	Score	rdb	e⁻ Conf	N-Rule
765.2172	1	C38H26N14NaO4	765.2154	2.3	3.0	1	47.24	32.5	even	ok
	2	C41H34N4NaO10	765.2167	0.6	5.9	2	100.00	26.5	even	ok
	3	C40H38NaO14	765.2154	-2.3	11.5	3	40.89	21.5	even	ok
	4	C42H30N8NaO6	765.2181	1.2	12.7	4	69.63	31.5	even	ok
	5	C39H22N18Na	765.2167	0.6	12.9	5	87.47	37.5	even	ok
	6	C43H26N12NaO2	765.2194	2.9	25.4	6	21.50	36.5	even	ok
	7	C28H22N24NaO3	765.2199	3.6	39.7	7	9.73	29.5	even	ok
	8	C30H34N10NaO13	765.2199	3.6	49.2	8	7.35	18.5	even	ok

Figure S43. HRMS spectrum and measurement for jugione D (**4**)

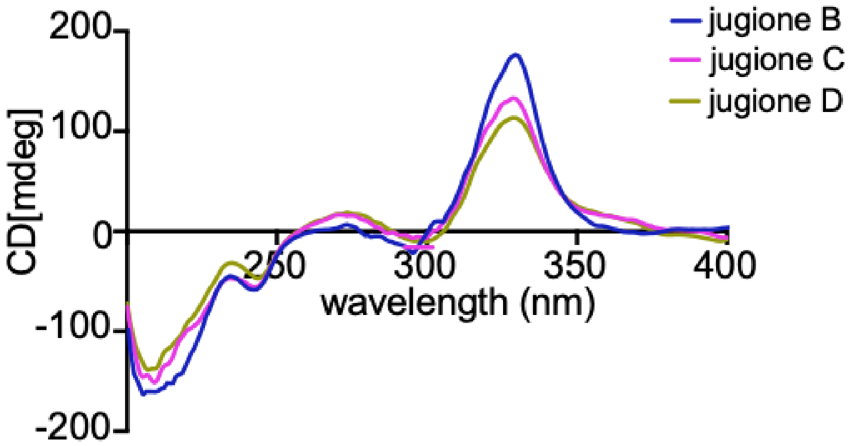

Figure S44. ECD spectra of jugiones B–C (**2**–**4**) in MeOH (0.5 mg/mL).

3. Biological assay results for jugiones A–D (1–4)

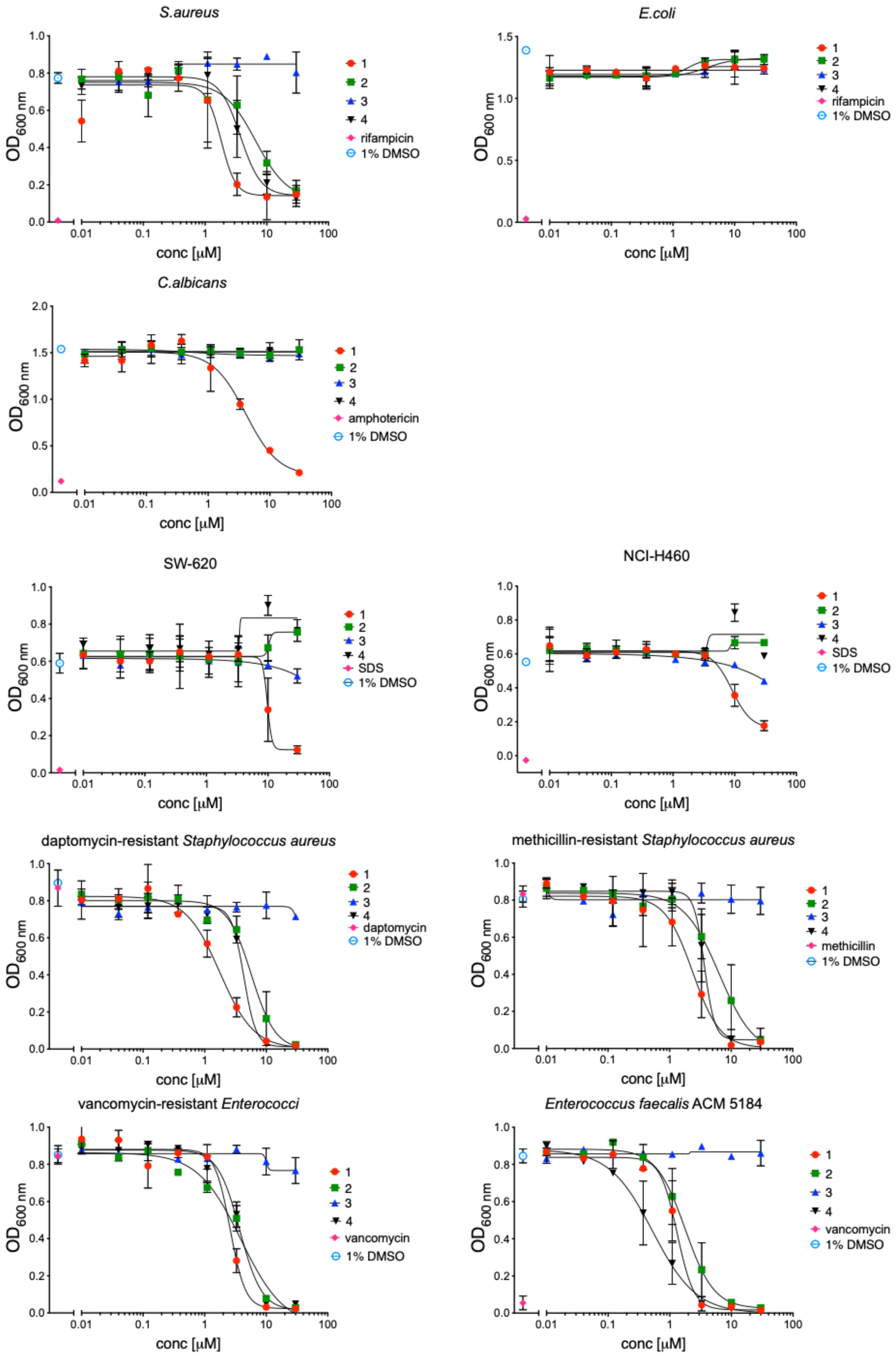

Figure S45. Antimicrobial and cytotoxicity activity of metabolites **1–4.**

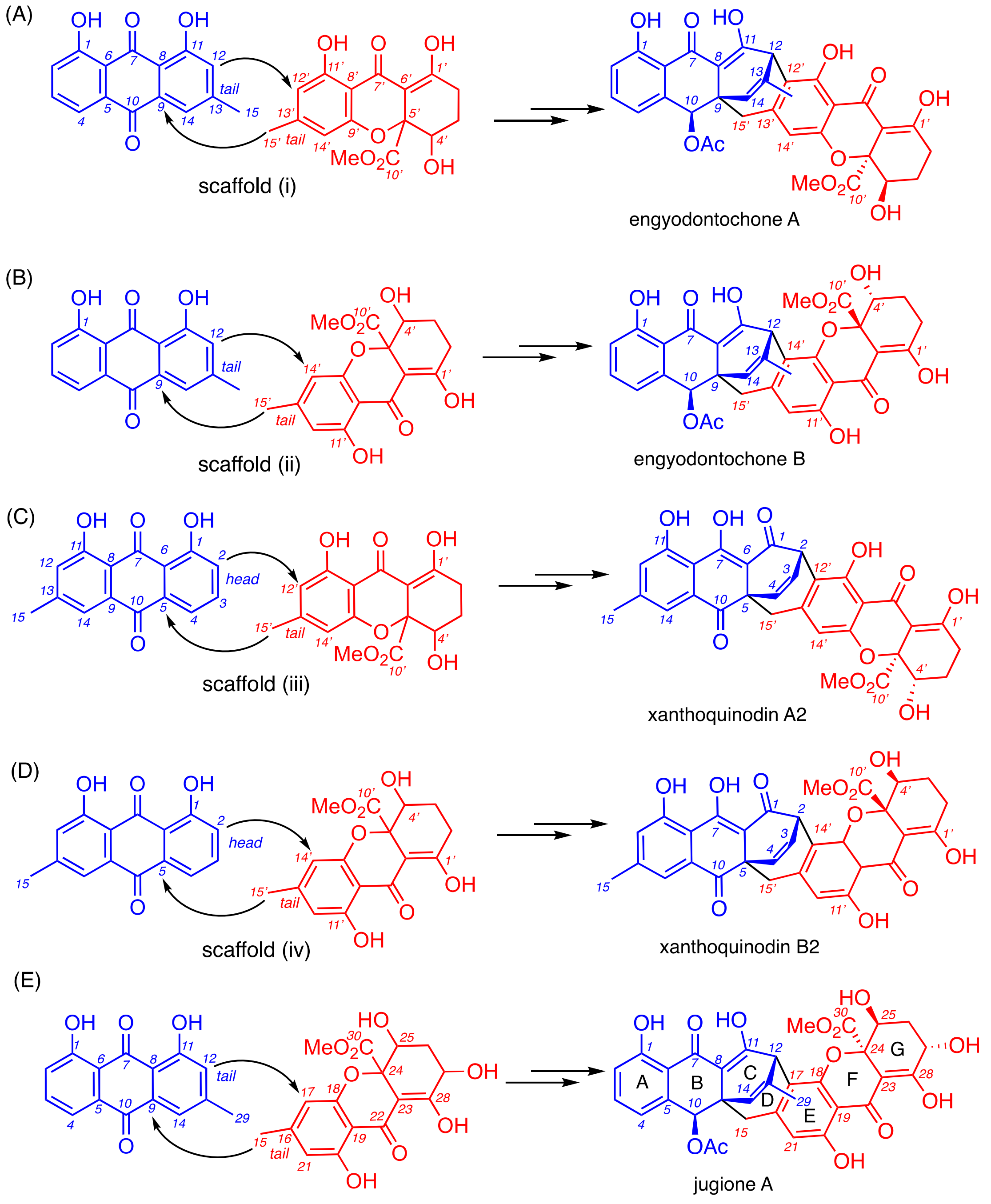

Figure S46. (A-D) Different scaffolds arising from the xanthone and anthroquinone dimerization, with examples of compounds reported from the literature. (E) Dimerisation to give jugione A (scaffold ii), with numbering of the monomeric units reflecting those of the final product.

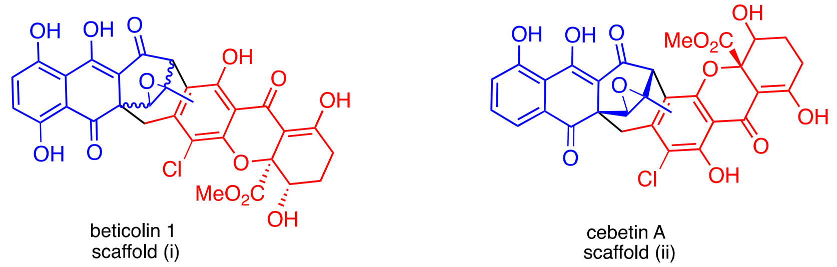

Figure S47. Structures of beticolin and cebetin.

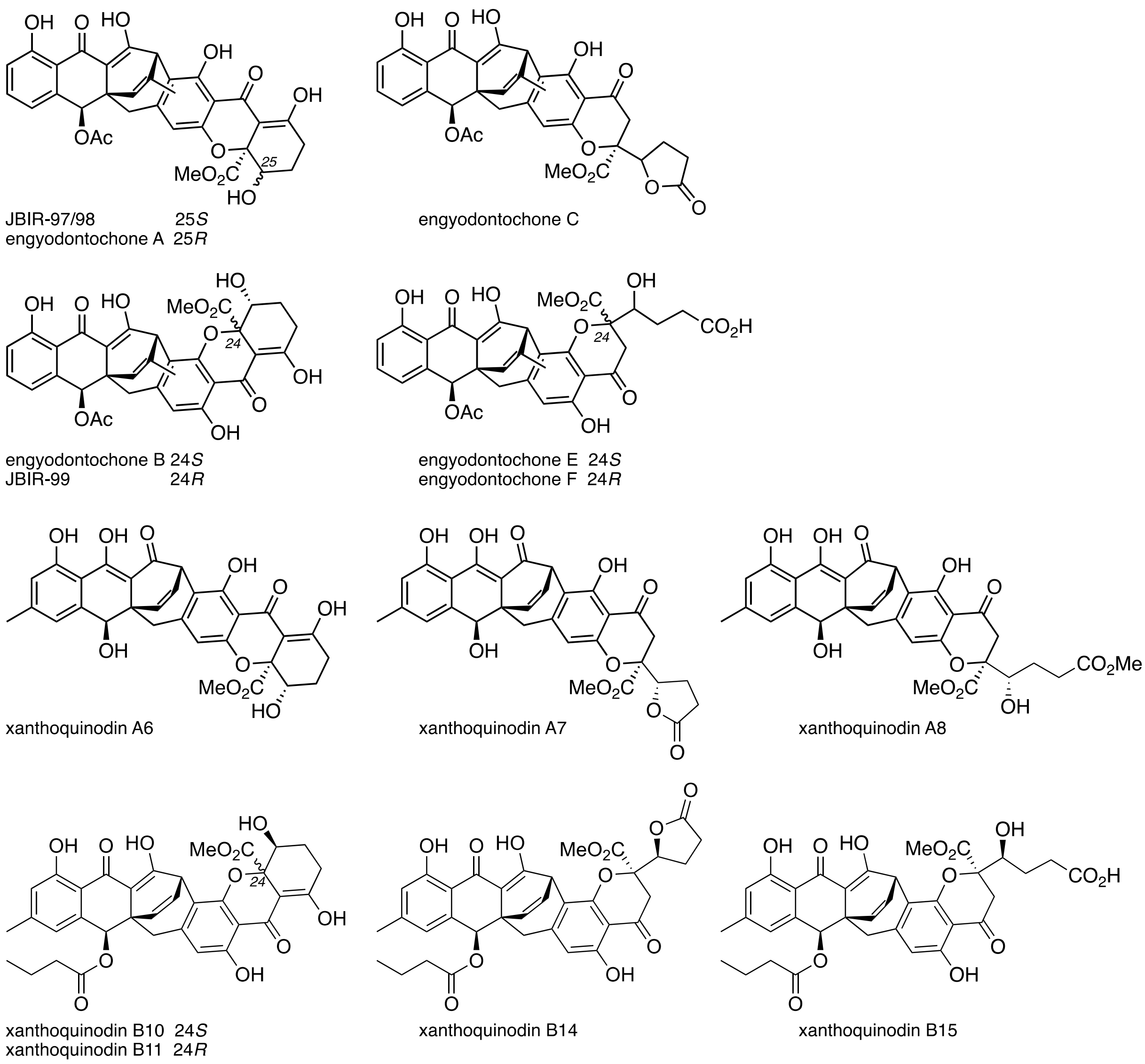

Figure S48. Structures of xanthone-anthroquinone dimers
